# Modified pea apyrase has altered nuclear functions and enhances the growth of yeast and Arabidopsis

**DOI:** 10.3389/fpls.2025.1584871

**Published:** 2025-05-26

**Authors:** Manas K. Tripathy, Huan Wang, Robert D. Slocum, Han-Wei Jiang, Ji-Chul Nam, Tanya Sabharwal, Roopadarshini Veerappa, Katherine A. Brown, Xingbo Cai, Peter Allen Faull, Greg Clark, Stanley J. Roux

**Affiliations:** ^1^ Department of Molecular Biosciences, University of Texas at Austin, Austin, TX, United States; ^2^ Department of Biological Sciences, Goucher College, Towson, MD, United States; ^3^ The Oden Institute for Computational Engineering and Sciences, University of Texas at Austin, Austin, TX, United States; ^4^ Cavendish Laboratory, University of Cambridge, Cambridge, United Kingdom

**Keywords:** apyrase, calmodulin, DNA-binding, phosphate, point mutation, seed yield

## Abstract

Apyrases (NTPDases) regulate growth and development in multiple eukaryotic organisms and function in multiple sub-cellular locales. An earlier report showed that the ectopic expression of psNTP9 (PS), a chromatin-associated pea (*Pisum sativum*) apyrase, enhanced the uptake of inorganic phosphate (Pi) and increased the growth of yeast and Arabidopsis. In this follow-up study, we generated a modified form of PS, abbreviated DM (“double mutant”), in which two-point mutations, S208L and P216R, were introduced into its DNA-binding domain. Ectopic expression of DM increased the growth of yeast and Arabidopsis, the seed yield of Arabidopsis, and the Pi content of yeast and Arabidopsis grown in Murashige-Skoog media beyond that effected by PS. Both the PS and DM proteins co-purified with nuclei and chromatin-associated proteins from yeast and Arabidopsis, and expression of their transgenes in these model organisms produced gene expression profiles that would be expected to promote increased growth and Pi uptake. Chromatin immunoprecipitation (ChIP)-seq analyses showed that PS and DM have largely different binding sites on yeast chromatin, including sites in promoters of numerous genes that are differentially-expressed in *PS* and *DM* transgenic lines. These results are consistent with the hypothesis that the effects of ectopically expressing the pea apyrase in yeast and in Arabidopsis are mediated, at least in part, by its activities in the nucleus that impact transcription.

Apyrase enzymes are nucleoside triphosphate diphosphohydrolases (NTPDases) that hydrolyze the g- and b-phosphate of adenosine triphosphate (ATP) and the b-phosphate of ADP (Plesner, 1995). Among the better characterized plant apyrases is one in pea (*Pisum sativum*), psNTP9, hereafter referred to as PS. It has been localized both in the nucleus (Chen and Roux, 1986; Chen et al., 1987; Shibata et al., 2002), and in the extracellular matrix (ECM; Thomas et al., 1999; Shibata et al., 2002), consistent with PS having both a nuclear-localization signal and a signal peptide (Hsieh et al., 1996). The activity of PS purified from the nuclei of etiolated pea seedlings is stimulated more than 3-fold by Ca^2+^-activated calmodulin (CaM) (Chen et al., 1987), a signaling protein that localizes to and functions in both the nucleus (Bouché et al., 2002) and the ECM (Cui et al., 2005).

Plant proteins can be efficiently expressed in yeast, where they localize and function similarly as they do in plants (Meskiene et al., 1998; Yang et al., 2022). Previously, Thomas et al. (1999) reported the effects of transgenically expressing the *PS* gene in Arabidopsis and in a yeast mutant (NS219) deficient in *Pho84*, a gene that helps regulate both the uptake of phosphate (Pi) and the transcription of genes that mediate the response of yeast to Pi starvation (Austin and Mayer, 2020). They found that *PS* expression enhanced growth and increased Pi uptake in both organisms (Thomas et al., 1999), although the mechanisms by which this occurred were largely unexplored.

Site-directed mutations of specific proteins can improve Pi content and growth in plants (Fontenot et al., 2015). As reviewed by Gonzalez-Perez et al. (2012), *Saccharomyces cerevisiae* is an effective choice for characterization of heterologously expressed eukaryotic proteins with altered properties. For example, Yang et al. (2019) found that point mutations in a plasma membrane H+-ATPase (AHA2) could complement plasma membrane H^+^-ATPase activity in yeast.

We tested whether directed mutations could confer upon *PS* an even greater potential to promote growth and enhance Pi content in yeast and Arabidopsis beyond that reported earlier by Thomas et al. (1999). One set of mutations that was of particular interest to us was in a PS domain that was one of two it has that have general features of many CaM-binding sites: Potential Calmodulin Binding Domain (PCBD)1 and PCBD2 (Hsieh et al., 2000). Although prior studies had shown that only the PCBD2 peptide could bind CaM in a Ca^2+^-dependent manner, the PCBD1 peptide was predicted to require only two amino acid changes to enhance its CaM binding (Hsieh et al., 2000), S208L and P216R. A helical wheel representation of PCBD1 thus altered has an amphipathic alpha-helical structure with basic residues aligned along one side and hydrophobic residues along the other side of the helix, characteristic of many CaM-binding domains (Hsieh et al., 2000), Additionally, PCBD1 has a helix-turn-helix and other structural features that give it an affinity to bind DNA (Brennan and Matthews, 1989), and Chen (1987) confirmed that PS binds with high specificity to a DNA-affinity column. Thus, by introducing two point mutations into the PCBD1 a-helix of PS (S208L, P216R), we could potentially both enhance its CaM binding and alter its affinity for DNA. Changing the binding of PS to either CaM or to DNA could be expected to modify how it functions in cells. Here we test how the DM change in PS impacts the effects of its expression on chromatin interaction, gene expression, Pi content and growth of yeast and Arabidopsis, and we investigate whether changes in these effects are likely due to differences between PS and DM in their CaM binding and/or to their altered nuclear functions.

## Materials and methods

### Construction of DM by site-directed mutagenesis of PS

PCR-based site-directed mutagenesis was used to modify the PCBD1 sequence in the PS. The mutagenic primers were designed in such a way that the PCR product contains the single nucleotide replacement at a time. The first pair primer introduced a C to T mutation (S208L). The recombinant plasmid pK7WG2:psNTP9 used as template for PCR amplification. The PCR product was transformed into bacteria as per the manufacturer’s protocol (QuikChange II XL Site-Directed Mutagenesis Kit, Agilent Technologies). Transformed bacteria were plated on LB kanamycin selection plates. The colony was confirmed by PCR using psNTP9 specific primers. Plasmid DNA was isolated from confirmed colonies and the psNTP9 mutation was confirmed by DNA sequencing using CaMV 35S promoter forward and terminator reverse primers. The single mutant plasmid DNA was used as template along with the second pair of primers, which introduced a C to G mutation (P216R). Similar to the first step, the transformed colony was confirmed by PCR, plasmid DNA was isolated and the double mutation was confirmed by DNA sequencing. The confirmed double mutant (S208L, P216R) plasmid pK7WG2:psNTP9-DM was further used for Arabidopsis transformation. The primer sequences are listed in **Supplementary Table S1**.

### Yeast PS and DM expression constructs

The yeast (*Saccharomyces cerevisiae*) strain NS219 (*pho3-1 leu2-3,112 ura3-52 pho84-1*), a Pi transport defective mutant (Bun-Ya et al., 1991) was provided by Dr. Maria J. Harrison (Cornell University). The coding region of the pea (*Pisum sativum*) apyrase cDNA (*PS;* GenBank accession # Z32743.1) was cloned into pYES2 (Invitrogen, Carlsbad, CA) downstream of the *GAL1* promoter, and transformed into yeast, as described by Thomas et al. (1999). The pYES2psNTP9-DM and pYES2-PS-NLS constructs were made by subcloning the full-length DM and PS-NLS cDNAs from the plant transformation vectors pK7WG2:psNTP9-DM and pB7FWG2:psNTP9-NLS, respectively (see below). Expression constructs for 13Myc-tagged PS and DM proteins for use in ChIP-seq assays were made as follows. A linker + 13Myc tag was fused to the C-terminal end of the pYES2-psNTP9 or pYES2-psNTP9-DM. The 5’-GGAGGTGGAGGTTCA-3’ linker was added to make the Myc tag more flexible and less likely to affect PS and DM function. The 13Myc sequence was amplified from the pFA6a-13MycTRP1 vector using primers HW1 and HW2 (**Supplementary Table S1**). The entire plasmid was amplified using PCR primers HW3 and HW4 (**Supplementary Table S1**) to remove the stop codon at the end of the PS or DM open reading frames. Following, Sal I and Sac II digestion of vector and 13Myc DNA, the sticky ends were ligated using T4 DNA ligase. DM, PS-NLS, 13Myc-PS and 13Myc-DM constructs were confirmed by colony PCR and sequencing, then transformed into the NS219 yeast strain using the Frozen-EZ Yeast transformation II kit (ZYMO Research).

### Growth of yeast

**Figure 1:**
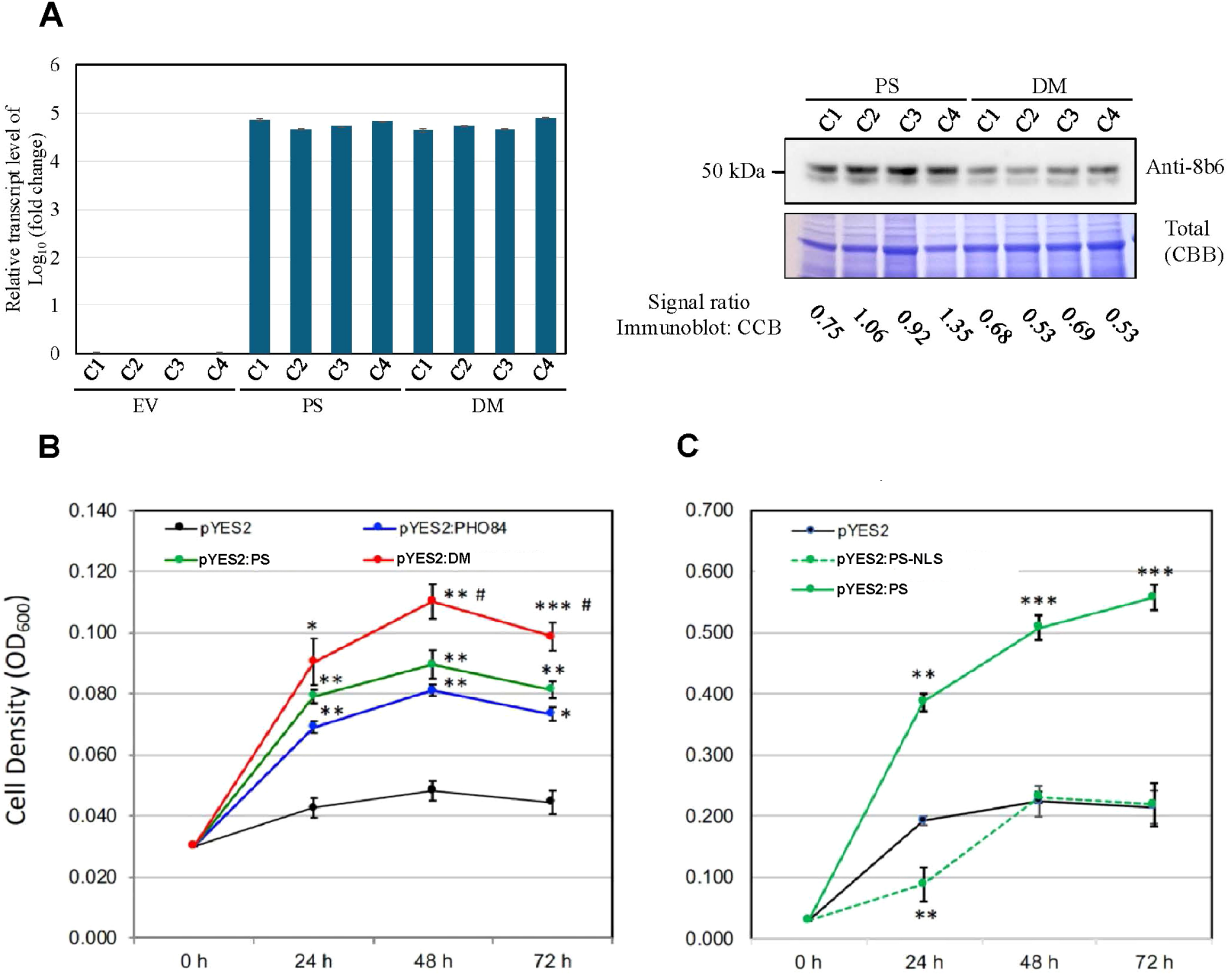
*PS* and *DM* transcript and protein levels, and growth of yeast pho84 mutant NS219 constitutively expressing *PS* and *DM*. **(A)** Left panel, relative transcript abundance in different colonies **(C)** of empty-vector (EV)-expressing pYES2, PS-expressing pYES2, and DMexpressing PYES2. The qRT-PCR samples were from 72 h induced pho84 yeast cells, under the same induction condition as in panels B and C, with four biological repeats. Relative transcript levels were normalized using the expression levels of UBC6 for qRT-PCR. P value ≤ 0.01. Right panel, top row, immunoblot indicating relative staining of PS and DM by monoclonal antibody 8B6 in extracts of the same colonies as in left panel; bottom row, relative protein staining by Coomassie Brilliant Blue (CBB) of proteins extracted from the same colonies as in left panel. **(B)** Growth curves for the mutant transformed with the pYES2 vector alone, with pYES2:PS, pYES2:DM and pYES2:PHO84 (positive control). Growth was in low-Pi medium (100 µM). **(C)** Growth curves for the mutant transformed with the pYES2 vector alone, pYES2:PS, or with pYES2:PS-NLS. Values represent the means ± SE of three independent transformants, each with three technical repeats. An asterisk indicates that the mean value was significantly different (*p < 0.05, **p < 0.01, ***p < 0.001) from that of the pYES2 vector control and # indicates mean value of pYES2:DM was significantly different (p ≤ 0.05) from that of the pYES2:PS when analyzed by one-way ANOVA (Tukey’s Multiple Comparison Test).

For time-course growth assays, yeast NS219 transformed with pYES2 vector alone (empty vector (EV) control), pYES2-PHO84 (positive control), pYES: PS or pYES2:DM were initially cultured in 4 mL liquid yeast nitrogen base (uracil) with glucose pH 5.2. The culture was grown at 30^°^C for 48h on a 250-rpm shaker. After 48h, the cultures were inoculated (starting OD_660_ = 0.03) into 3.5 mL of filter-sterilized synthetic defined induction medium containing 2% Galactose, 0.1% Glucose, 5% glycerol, and 100 µM KH_2_PO_4_, pH 6.5. The growth was monitored spectrophotometrically at OD_660_ at defined time intervals. Growth and induction conditions for pYES2:PS-NLS (**Figure 1C**) were the same as for pYES2:PS in panels A and B of **Figure 1**.

### Purification of nuclei and chromatin-associated proteins from yeast

Yeast nuclei were isolated following the method of Niepel et al. (2013), except that a polytron homogenizer was used for spheroplast lysis. Yeast chromatin was prepared following the method of Cuevas-Bermúdez et al. (2019), with the following modifications: Yeast cells grown to OD_600_ ~ 0.7–0.8 in URA- dropout media were lysed by 5 freeze-thaw cycles in liquid nitrogen then vortexed for 4 min. The resuspension buffer 2 used was 250 mM NaCl.

### Mass spectrometry analysis of the association of PS and DM with the yeast chromatinassociated proteome

Chromatin-enriched fraction pellets of three biological replicates for each of the three conditions were solubilized in 20 µL of Tris-HCl 50 mM, pH 7.5, and submitted (N = 9 samples) for mass spectrometry analysis at the Biological Mass Spectrometry Facility, UT Austin Center for Biomedical Research Support (RRID: SCR_021728). Prior to LC-MS analysis, proteins were reduced, alkylated and digested with trypsin and the resulting peptides were either manually or robotically desalted using Millipore U-C18 ZipTip pipette tips following the manufacturer’s protocol. Data were acquired using an Orbitrap Fusion Tribrid mass spectrometer equipped with an ultra-high-pressure Ultimate 3000 RSLC nanosystem (Thermo Scientific). Raw data files were analyzed using MaxQuant version 2.0.3.0 (Cox and Mann, 2008). The *Saccharomyces cerevisiae* reference proteome (UP000002311) containing 6,067 protein sequences was downloaded from UniProt in January 2022 and searched alongside the MaxQuant contaminant protein database. MaxQuant generated a decoy protein database by reversing the provided yeast proteome database. The LFQ protein quantification strategy (Cox et al., 2014), match between runs, and a 1% false discovery rate at both the peptide spectrum match and the protein level were employed. The results were imported into Perseus version 1.16.15.0 (Tyanova et al., 2016) and filtered to remove contaminant proteins, reversed sequences, and identified only by site entries. The resulting 2,379 proteins were median normalized within replicates.

### Arabidopsis growth conditions

Arabidopsis (*Arabidopsis thaliana*) ecotype Columbia (CS907) was used as wild type. Seeds were stratified at 4°C in the dark for 3 d followed by white light exposure (50 μmol m^−2^ s^−1^) for 3 d to promote germination. Seeds were planted directly on autoclaved Metro-Mix 200 soil or surface sterilized and planted on solidified Murashige and Skoog (MS) medium (4.3 g/L, MS salts [Caisson], 0.5% [w/v] MES, 1% [w/v] sucrose, and 1% [w/v] agar, raised to pH 5.7 with 5 M KOH). For Pi assays, seedlings were grown upright in continuous light. For nuclear localization experiments, seedlings were grown in the dark. All seedlings were grown at 23°C.

### Construction of transgenic Arabidopsis lines overexpressing DM

For transformation, the pK7WG2:psNTP9-DM recombinant plasmid was transformed into *Agrobacterium tumefaciens* strain GV3101 (pMP90) using the freeze-thaw method and infected to Arabidopsis (Col*-*0) using the floral dip method (Zhang et al., 2006). Several homozygous transgenic lines were developed. Arabidopsis was also transformed with pK7WG2 only to produce empty-vector control homozygous plants. Homozygous transgenic *Arabidopsis* plants from T3 generation were used for all experiments. Total genomic DNA was isolated from leaf tissue using DNeasy Plant Mini Kit (Qiagen).

### Immunoblot analyses of nuclear proteins from yeast and Arabidopsis

Arabidopsis nuclei were purified using the method of Folta and Kaufman (2007), with minor modifications, as described by Weeraratne (2019). Immunoblot analyses of nuclear proteins from yeast and Arabidopsis were carried out as previously described (Sabharwal et al., 2022).

### Construction of PS with N-terminal and NLS deletions and expression in Arabidopsis

The nucleotide sequences for N-terminal residues Met-1 to Glu-39 were deleted from the psNTP9 cDNA to construct psNTP9-N. Primers NTP9-F-N and NTP9-R (**Supplementary Table S1**) were used to amplify the psNTP9 cDNA minus N-terminal sequences, followed by subcloning into pCR8/GW/TOPO-TA (Thermo Fisher Scientific). The NLS sequence (KKTAKNAPKVADGDDPYIKKVVLK) was removed from the cDNA of psNTP9 by overlap extension PCR. Primers (**Supplementary Table S1**) targeted each end of NTP9 (NTP9-F and NTP9-R) and two flanking NLS regions complementary each other (NTP9-NLS-NR and NTP9NLS-CF). The 5’- and 3’- regions of psNTP9 were amplified using primer pairs NTP9-F + NTP9-NTP9-NLS-NR and NTP9-NLS-CF + NTP9-R, respectively, followed by a second amplification using the overlapping 5’- and 3’- fragments as templates to generate NLS-deleted sequences (psNTP9-NLS). The cDNA with deleted NLS sequences was then sub-cloned into pCR8/GW/TOPO-TA. The N-terminal deletion and NLS deletion constructs of psNTP9 containing the stop codon were transferred by LR reaction from the pCR8/GW/TOPO entry vectors into the destination vector pB7FWG2 carrying the CaMV35S promoter. The resulting expression vectors were transformed into *Arabidopsis* Col-0 plants by Agrobacterium-mediated floral dip method (Zhang et al., 2006) and selected on media with 10 µg/mL of glufosinate ammonium. Expression of psNTP9-N and psNTP9-NLS was confirmed by RT-PCR using template-specific primers (**Supplementary Table S1**).

### Hairy root transformation and growth conditions

Full-length psNTP9 and psNTP9-DM cDNAs were cloned in binary vector pK7WG2. For hairy root transformation pK7WG2 (EV), pK7WG2:psNTP9 and pK7WG2:psNTP9-DM recombinant plasmid was mobilized into *Agrobacterium rhizogenes* ARqua1 via electroporation. Overnight LB cultures of *A. rhizogenes* strains were placed into a 1-mL tuberculin syringe fitted with a 30gauge needle. The needle tip was used to make wounds of 2–3 mm in length in the hypocotyls of soybean, corn, canola seedlings grown on MS medium. During the wounding process, droplets of the *A. rhizogenes* culture were injected into the wounds. The transformed seedlings were maintained with MS medium having 3% sucrose, 1.25 mM KH_2_PO_4_ containing kanamycin. The cultures were kept at 16 h light and a temperature of 22°C (Hatlestad et al., 2012).

### Total Pi content assays for yeast, Arabidopsis seedlings, and hairy root cultures

WT, PS2 and DM4 Arabidopsis seedlings were grown on MS agar plates in a growth chamber at 23°C in continuous light for 7 d. Their total Pi contents were measured using the modified ammonium molybdate procedure as described in Lapis-Gaza et al. (2014). Whole seedlings were harvested and homogenized at a ratio of 1 mg of tissue to 10 µL of 1% (v/v) acetic acid. The homogenized sample was centrifuged at 12,000 x *g* for 15 mins at 4°C. After centrifugation, 100 µL of supernatant was combined with 200 µL of Pi Color Solution (0.35% (w/v) NH_4_MoO_4_, 1.4% (w/v) ascorbic acid in 1N H_2_SO_4_) for 60 min at 42°C in darkness. After incubation, absorbance of samples was measured at 820 nm. Sample Pi concentrations were extrapolated from a standard curve generated using dilutions of KH_2_PO_4_. Independent experiments (3-5 biological replicates; 10-20 seedlings each) were repeated twice for wild type and transgenic lines.

The Pi contents of yeast grown in synthetic induction medium containing 0.1 M Pi were assayed as follows. For assays of Pi contents after 72h phosphate limitation, cells were pelleted, resuspended in ddH_2_O, then re-pelleted. This procedure was repeated 5 times to remove external Pi from the medium. For Pi uptake studies, cells in the 72h cultures were pelleted, then resuspended in the same medium containing 1 mM Pi for 15 minutes. Cells were then pelleted and rinsed with ddH_2_O, as described above. For both assays, cells were then acid hydrolyzed overnight at 42°C in 1% (v/v) acetic acid. After centrifugation (16,000 x *g*, 10 min), total Pi was measured in hydrolysates using the method described above and normalized on the basis of OD_600_.

### Bioinformatics

Putative membrane-spanning a-helices were predicted from the PS or DM sequences using RHYTHM (Rose et al., 2009). Clustal Omega (Sievers et al., 2011) alignment of PS and related plant apyrase sequences facilitated analyses of conserved sequences in these enzymes. Statistically over-represented gene sets (≥ 2-fold enrichment; FDR p ≤ 0.05, Fisher test) in Gene Ontology (GO) Biological Process categories were identified using the PANTHER classification system v 18.0 (http://www.pantherdb.org; Mi et al. (2019). Sequence-based calmodulin bindingsite predictions were done using the CaMELS webserver (Abbasi et al., 2017).

### Structural studies of PS and DM

The Phyre2 web portal (http://www.sbg.bio.ic.ac.uk/phyre2; Kelley et al. (2015) for sequence-based protein modeling was used to visualize structures of mature PS and DM apyrases. The “one-to-one” threading tool was used to perform template-based modeling of PS and DM on the structure of the white clover (*Trifolium repens*) apyrase complexed with AMP (PDB: 5U7V; Summers et al. (2017). The relaxed PCBD1-DM structure was confirmed by direct modeling with CollabFold v.1.5.3 (Mirdita et al., 2022) using the precursor DM sequence as input.

Synthetic peptides PCBD1, PCBD2, and PCBD1-DM were modeled using Pep-Fold 3.5 (Lamiable et al., 2016). Models were viewed and manipulated using the EzMol structure viewer (Reynolds et al., 2018). Space-filling models of PS and DM structures were generated with the CLC Genomics Workbench structure modeling software.

### Assay of the binding of biotinylated CaM to PS and DM

Recombinant His_6_-tagged CaM was expressed and purified using Ni-NTA affinity chromatography as previously described (Massalski et al., 2015). Biotinylated CaM was prepared using a Biotin Protein Labeling Kit (catalog no. 11418165001, Roche) following the manufacturer’s instructions. Protein concentrations of the PS and DM crude yeast extracts were measured using the Bio-Rad Protein Assay (Bradford method). The same amount of total protein was loaded in each lane for analyses. The yeast extracts were separated by SDS-PAGE and transferred onto a PVDF membrane as previously described (Sabharwal et al., 2022). The immunoblot in panel B of **Supplementary Figure S3** used the 8B6 antibody as described by Veerappa et al. (2019), and was performed first to verify equal loading of apyrase in the PS and DM samples. After confirming comparable expression levels, the same PVDF membrane was stripped and used for the subsequent biotinylated-CaM assay shown in panel A. The membrane was blocked with 5% BSA in PBST for 1 hour at room temperature. The membrane was then incubated with biotinylated CaM at a final concentration of 200 ng/mL in binding buffer (PBST with 2 mM CaCl_2_) at 4°C for 1 hour with gentle shaking. After washing three times with PBST, the membrane was incubated with HRP-Streptavidin (catalog no. RABHRP3, Sigma) for 1 hour at room temperature. CaM protein binding was detected using chemiluminescence (ECL). A control assay was performed in the absence of Ca^2+^ by substituting CaCl_2_ with 5 mM EGTA in the binding buffer.

### Transcriptome-level gene expression analyses in yeast and Arabidopsis

For yeast RNA-seq assays cultures of yeast transformed with vectors pYES2, pYES2-psNTP9 or pYES2-psNTP9-DM were grown for 3 days, as described above. Total RNA was isolated from pelleted cells according to Shedlovskiy et al. (2017). cDNA library preparation and Illumina sequencing (NovaSeq 6000 SR100 platform; 100 bp single-end reads) using the Tag-seq method (Lohman et al., 2016) was carried out by the Genome Sequencing and Analysis Facility (GSAF) at the University of Texas at Austin. Reads mapping and sample quality control analyses were performed as described by Veerappa et al. (2019) for three biological replicates. Reads were trimmed, mapped to the S288c reference genome and annotated (R64-4-1; 8-30-2023). An average 7.45 million reads were mapped to protein-coding genes/sample. Sequence data have been deposited in the NCBI Sequence Read Archive (accession #PRJNA1071135).

For Arabidopsis RNA-seq assays, growth of plants, RNA isolation and transcriptome-level analyses of differentially-expressed (DE) genes in 7-day-old plants ectopically expressing PS (PS2 line) or DM (DM4 line) were carried out as previously described (Veerappa et al., 2019). RNA-Seq data have been deposited in the NCBI Sequence Read Archive (accession #PRJNA449074).

Quantitative RT-PCR (qRT-PCR) analyses of pYES2, pYES2:psNTP9 and pYES2:psNTP9-DM expressing yeast and for WT, PS and DM expressing Arabidopsis seedlings were carried out. For the yeast assays, total RNA was extracted from 72h induced yeast grown in synthetic medium, and four biological replicates were used for each yeast strain. A single-step method was used for the isolation of high-quality RNA from yeast cells was described by Shedlovskiy et al. (2017). Total RNA concentration and purity were measured using Thermo Scientific NanoDrop 1000 spectrophotometer. Approximately 1 µg of total RNA was reverse transcribed using RevertAid First Strand cDNA Synthesis kit (K1621) from Thermo Scientific, following the manufacturer’s protocol.

For the Arabidopsis qRT-PCR analyses, total RNA was extracted from Col-0 WT, PS2 and DM4 seedlings grown on MS agar plates in a growth chamber at 23°C in continuous light for 7 d. Total RNA concentration and purity were measured using Thermo Scientific NanoDrop 1000 spectrophotometer. Approximately 1 µg of total RNA treated with Amplification Grade DNase I was reverse transcribed using a High-Capacity cDNA Reverse Transcription kit (AB Biosystems/Thermo Fisher, Waltham, MA, USA).

qRT-PCR was performed using the Life Technologies/AB Biosystems Quant 7 Real-Time PCR System. Power SYBR Green PCR Master Mix (Life Technologies/AB Biosystems, Waltham, MA, USA) was used for RT-qPCR reactions. The qRT-PCR conditions used were hold stage: 50°C, 2 min and 95°C, 10 min; PCR stage: 40 cycles of 95°C, 15 s and 60°C, 1 min. Melting curves generated from machine dissociation conditions were used to identify primer dimers and multiple targets. The primer sequences used are listed in **Supplementary Table S1**.

For each biological replicate, 3 technical triplicates with ±0.5 CT were used for qRT-PCR analysis. The relative gene expression was calculated by 2^-ΔΔCT^ method. *ALG9* was used as the reference gene in the yeast qRT-PCR assays and *Actin* or *PP2A* was used as the reference gene in the Arabidopsis qRT-PCR. Statistical significance between treatments was calculated by Student’s *t* test, using ΔCT for biological replicates.

### ChIP-seq analyses of PS and DM binding sites in yeast chromatin

#### ChIP-seq assay

Yeast chromatin immunoprecipitation were following the method of Park et al. (2014), with the modification that we used 300 mL of the yeast culture. *PS-* and *DM*-expressing yeast were induced in galactose medium for 4h. Glucose-treated *PS-* and *DM*-expressing yeast were used as negative controls. Immunoprecipitation of Myc-tagged PS and DM protein-DNA complexes was carried out overnight at 4°C on a rotary shaker by addition of 150 µL of a 50% suspension of EZview Red Anti-c-Myc Affinity Gel (Sigma-Aldrich) to 500 µL of the whole cell extract.

#### Identification of PS and DM binding sites

DNA samples were sequenced by the Genome Sequencing and Analysis Facility (GSAF) at the University of Texas at Austin (Illumina NovaSeq SP, PE150 reads). ChIP-seq analyses were carried out using the CLC Genomics Workbench 10.0.1 platform (Qiagen). Reads were trimmed and mapped to the yeast S288c reference genome with an average of 26.3 x 10^6^ unique reads/sample and a normalized average 464x genome coverage. The non-redundant fraction of unique/total reads (0.5-0.6) confirmed an acceptable library complexity. The Transcription Factor ChIP-Seq analysis module was run to identify protein-binding sequences (peak calling) in test samples (induced, non-induced PS and DM samples), relative to non-immunoprecipitated DNA from whole cell extracts of the same samples (input controls). Peaks were further annotated with S288c genome coordinates for genes. Unique peaks in induced PS or DM samples were further identified by filtering out peaks that overlapped in annotated tracks of both induced and non-induced samples (differential binding analyses). Peaks (p ≤ 0.05) located within genes or in promoter regions (-1,000 bp, relative to TSS) were selected for further analyses. Approximately half of the peaks were replicated in two independent assays (50.2% for PS, 64.4% for DM). Sample reads for these assays have been deposited in the Sequence Read Archive (#PRJNA1071121).

#### Motif discovery and enrichment analyses

Identification of yeast binding site motifs (*cis*-elements) in PS and DM ChIP-seq peak sequences was done using the Motif Enrichment/SEA module of the MEME Suite 5.5.5 (https://memesuite.org/). The PS or DM peak sequences were analyzed using the YEASTRACT motif database and shuffled input sequences as the control. MEME and XTREME modules were further used to discover fixed and variable length motifs and their enrichment, followed by comparison of identified motifs to yeast motifs in YEASTRACT using Tomtom. Sequence logo representations of motifs were generated using WebLogo3 (https://weblogo.threeplusone.com/create.cgi).

## Results

### Structural models of PS and DM, and potential CaM binding sites

**Figure 2:**
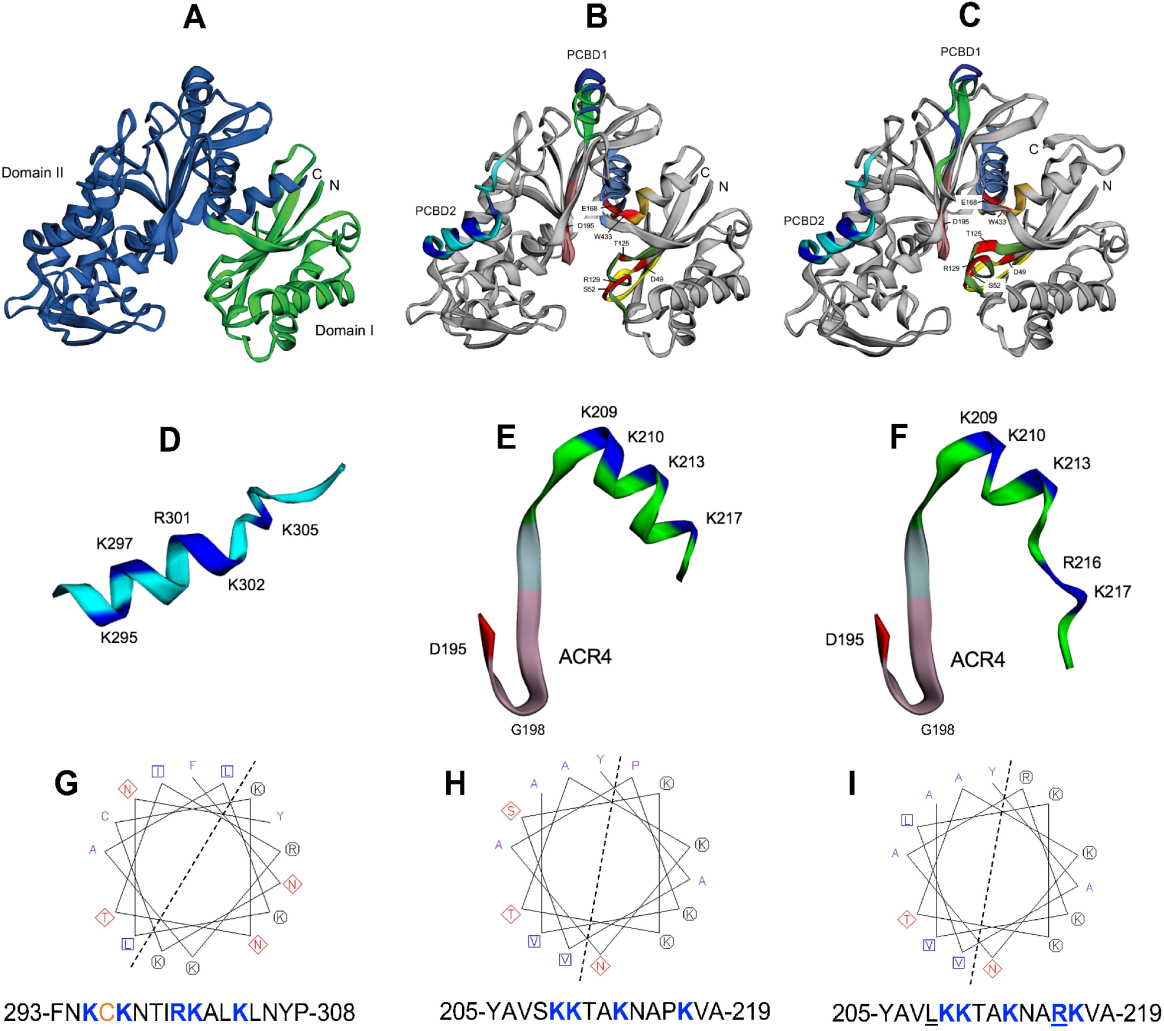
Structure models of apyrases psNTP9 and psNTP9-DM. **(A)** Hinged structure of the enzyme, connecting the N-terminal Domain I (green) and C-terminal Domain II (blue) is shown for psNTP9. **(B)** Conserved regions ACR1-5, as reported for the Trifolium apyrase, line the catalytic cleft of this enzyme (ACR1-yellow, ACR2-green, ACR3-light blue, ACR4-pink, ACR5-orange). Catalytic residues in ACR1-5 (red), identified by Summers et al. (2017) are labeled using psNTP9 numbering (see **Supplementary Figure S1**). CaM binding domain 2 (PCBD2 **(D)**; putative CaM binding domain 1 (PCBD1 **(E)**; and PCBD1-DM **(F)**, which is mutated (S208R, P216R) in psNTP9-DM **(C)** are shown, rotated 90° to the right from orientations within the enzyme structures. Communication between PCBD1, located at the hinge position in the enzyme, and N-terminal ACR4 and catalytic residue D195, which descends into the catalytic cleft, are illustrated by extended structures for PCBD1 in these enzymes. Helical wheel representation and sequence for residues in CaM-binding PCBD2 **(G)** illustrates the amphipathic character of this a-helix (dotted line). C-terminal sequences comprising the a-helix of PCBD1 in psNTP9 **(E)** and psNTP9-DM **(F)** are colored bright green; their helical wheels and corresponding sequences are shown below **(H, I)**.

The primary structure of PS is 78% identical to that of a *Trifolium repens* apyrase, whose crystal structure has been published (Summers et al., 2017). A structural model of PS is shown in **Figure 2A**, illustrating the hinged structure of the enzyme connecting the N-terminal Domain I and C-terminal Domain II. Based on alignment of residues 42-442 of the pea apo-apyrase with 397 residues of the Trifolium apyrase, apyrase conserved regions (ACR) 1-5 and catalytic residues, as reported for the *Trifolium* apyrase, line the catalytic cleft of PS (**Figures 2B, C**).

CaM binding domain 2 (PCBD2 (**Figure 2D**); putative CaM-binding domain 1 (PCBD1 (**Figure 2E**); and PCBD1-DM (**Figure 2F**), which is mutated (S208L, P216R) in psNTP9-DM are shown, rotated 90° to the right from orientations within the enzyme structures. The P216R mutation appears to result in an unwinding and extension of the PCBD1-DM a-helix and Cterminal loop within the protein structure, while maintaining overall character of this potential CaM binding site.

Functionally-characterized CaM-binding domain 2 (PCBD2) (Hsieh et al., 2000) exhibits an amphipathic a-helix character, with clustered basic residues, anchored on either side by hydrophobic anchor resides with a 1-14 spacing motif (**Figure 2G**). The a-helix in putative CaM binding domain 1 (PCBD1) (**Figure 2H**) and in PCBD1-DM (**Figure 2I**) displays a structure similar to that of PCBD2.

**Supplementary Figure S1** shows space-filling structure models that highlight the surface locations of PCBD1 and PCBD2, where clustered basic residues would be more likely to interact with the acidic sugar-phosphate backbone of DNA.

### Constitutive expression of PS and DM promotes growth in a yeast Pi-transport mutant

qRT-PCR analyses showed that the transcript level of *PS* in *PS*-expressing yeast and the level of *DM* in *DM*-expressing yeast were approximately equal. EV-expressing yeast had no *PS* or *DM* transcripts (**Figure 1A**, left panel). Immunoblot analysis showed that the protein level of PS and DM in their respective transgenic yeast are similar, but the DM level is somewhat lower (**Figure 1A**, right panel, top row). Loading control for the immunoblot indicated that the protein levels loaded from the PS and DM extracts were about equal (**Figure 1A**, right panel, bottom row). The 8B6 mAb was raised to a highly purified PS protein, and the peptide region it recognizes is near the N-terminal end of PS. In this region the primary sequences of PS and DM are identical, making it likely that the affinity of 8B6 for PS and DM would be similar.

Enhanced growth resulting from *PS* overexpression in yeast *pho84* mutant NS219, which is null for a major Pi transporter (Bun-Ya et al., 1991), was previously reported by Thomas et al. (1999). Expression of *DM* in this mutant resulted in an even greater increase of its growth (**Figure 1B**). Growth of yeast cells overexpressing a mutant PS, in which its nuclear localization signal had been deleted (PS-NLS) resulted in growth that was not different from the emptyvector control (**Figure 1C**).

### Transgenic expression of PS and DM significantly increases the phosphate content of yeast

**Figure 3:**
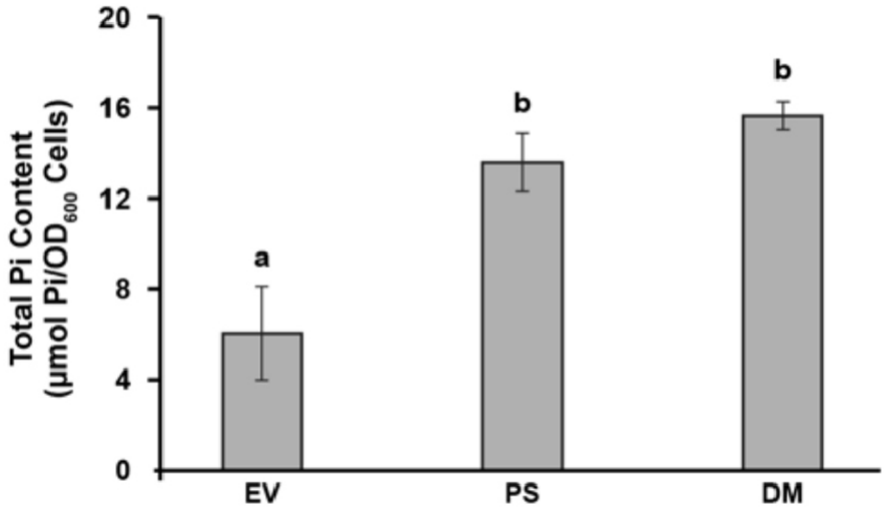
*PS* and *DM* overexpression significantly enhances the phosphate (Pi) content of yeast. Total Pi contents of yeast cells transformed with empty vector (*EV*), *PS* or *DM*. Yeast cells were grown for 72 h on 0.1 mM Pi, as in **Figure 1**, and transferred to 1 mM Pi medium for 15 min, then their Pi contents were measured. Data are means ± S.E., n = 4 biological replicates. Letters indicate significant differences (p < 0.05), as determined by one-way analysis of variance (ANOVA) with *post-hoc* Tukey honest significant difference (HSD) testing.

When the Pi content of *PS*- and *DM*-expressing yeast is assayed after their transfer to MS medium (1 mM Pi), the Pi content of both *PS*- and *DM*-expressing yeast is significantly higher than that of EV-expressing yeast (**Figure 3**). However, *PS* expression does not promote Pi uptake in yeast grown on low Pi media (Thomas et al., 1999), so when *PS*- and *DM*-expressing yeast are grown on 100 µM Pi medium, as in **Figure 1**, the Pi content of *PS*- and *DM*-expressing yeast is not significantly different from that of *EV*-expressing yeast (**Supplementary Figure S2A**).

### Biotinylated CaM binds to the PS and DM, but DM does not bind more CaM than PS, nor is it activated by CaM

When highly purified from pea nuclei, PS is activated more than 3-fold by CaM (Chen et al., 1987). As evaluated by a CaM-binding assay using ^35^S-labeled CaM, a bacterially-expressed and highly purified PS bound to CaM in the presence of Ca^2+^, under conditions in which the negative control did not bind the probe (Hsieh et al., 2000). Similarly, Steinebrunner et al. (2000), used biotinylated CaM to show that the Arabidopsis apyrase most similar to PS, AtAPY1, also bound CaM, and that the binding was Ca^2+^-dependent. We used the biotinylated CaM assay mainly to investigate whether the level of CaM bound to DM was different from that bound to PS when similar levels of the two apyrases were assayed. To confirm that similar levels of PS and DM were being compared, we used an immunoblot stained with monoclonal antibody 8B6, previously shown to recognize a peptide region that was identical in PS and DM (see Materials and Methods).

Results showed that PS and DM bind similar levels of Ca^2+^-activated CaM in crude extracts of *PS*- and *DM*-expressing yeast (**Supplementary Figure S3A**). Multiple other proteins found in yeast crude extracts can bind Ca^2+^-activated CaM, a result previously reported (Ohya and Anraku, 1992; Cyert, 2001). The 53 kDa band, which is detected both in the presence of Ca^2+^ and its absence (**Supplementary Figure S3A**, right panel), is likely a previously identified endogenous yeast biotinylated protein (van Werven and Timmers, 2006). In these transgenic yeast extracts, the presence of PS and DM in a band just above the 55 kDa marker was confirmed by the highly specific monoclonal antibody 8B6 (**Supplementary Figure S3B**). Even though the extracts had somewhat more immunoreactive DM than PS (**Supplementary Figure S3B**), the intensity of biotinylated CaM bound to DM was not significantly different from that bound to PS (**Supplementary Figure S3B**). Although the combined results of **Supplementary Figures S3A** and **B** make it clear that PS and DM are among the proteins in transgenic yeast that bind CaM, there could be another yeast protein near 56 kDa that contributes to the binding observed.

Because the two amino acid changes to the PCBD1domain of DM gave it an amphipathic helical wheel structure common to many CaM binding domains, a prediction was made that DM might bind more CaM than PS, but this prediction was not verified by the biotinylated-CaM binding assay (**Supplementary Figures S3A, B**). An *in silico* structural analysis of CaM-binding site predictions using CaMELS (Abbasi et al., 2017) helped resolve this discrepancy. The CaMELS analysis gave a high score of 1.76 for the potential of the PCBD2 CaM-binding sequence of PS to interact with CaM, but a very low score of 0.97 for the potential of the PCBD1 sequence of PS to interact with CaM (**Table 1**). The amino acid substitutions made in DM did increase the amphipathicity of the modified PCBD1, however, when incorporated into the intact DM protein, its CaM-interaction potential score was raised only to a still low value 1.23. indicating it would have a low probability of binding CaM. Even though the increased amphipathicity of the synthetic PCBD1-DM peptide did slightly increase its potential to bind CaM, the loop region of this modified domain is more buried than that of PCBD2 in the full structure of the protein, and it is disordered and not exposed enough to bind CaM.

**Table 1:** CaMELS prediction scores for PCBD1 and PCBD2 sites in PS and DM structure.

Protein	PCBD2 site	PCBD1 site
PS	1.76	0.97
DM	1.76	1.23

Multiple independent assays of partially purified extracts indicated that both PS and DM could hydrolyze ATP and ADP substrates, but not AMP. The specific activity of DM was about half that of PS. CaM stimulated PS activity only slightly, and did not stimulate DM activity (**Supplementary Table S2**).

### Transgenic expression of PS and DM significantly increases the phosphate content of hairy root cultures of soybean, corn and canola

In corn, soybean and canola hairy roots grown on MS medium, transgenic lines that express *PS* and *DM* have significantly higher Pi contents than hairy roots transformed with empty vector, and lines that express *DM* have significantly higher Pi content than hairy roots expressing *PS* (**Supplementary Figure S4A**). For each of the three different species of cultured hairy roots the comparisons of Pi content used were normalized per unit weight of the culture. PS and DM expression in transformed lines was confirmed by RT-PCR analyses (**Supplementary Figure S4B**), however the relative levels of PS and DM transcript and protein expressed in these cultures were not measured.

### Arabidopsis plants ectopically expressing DM have higher seed yield than those expressing PS

**Figure 4:**
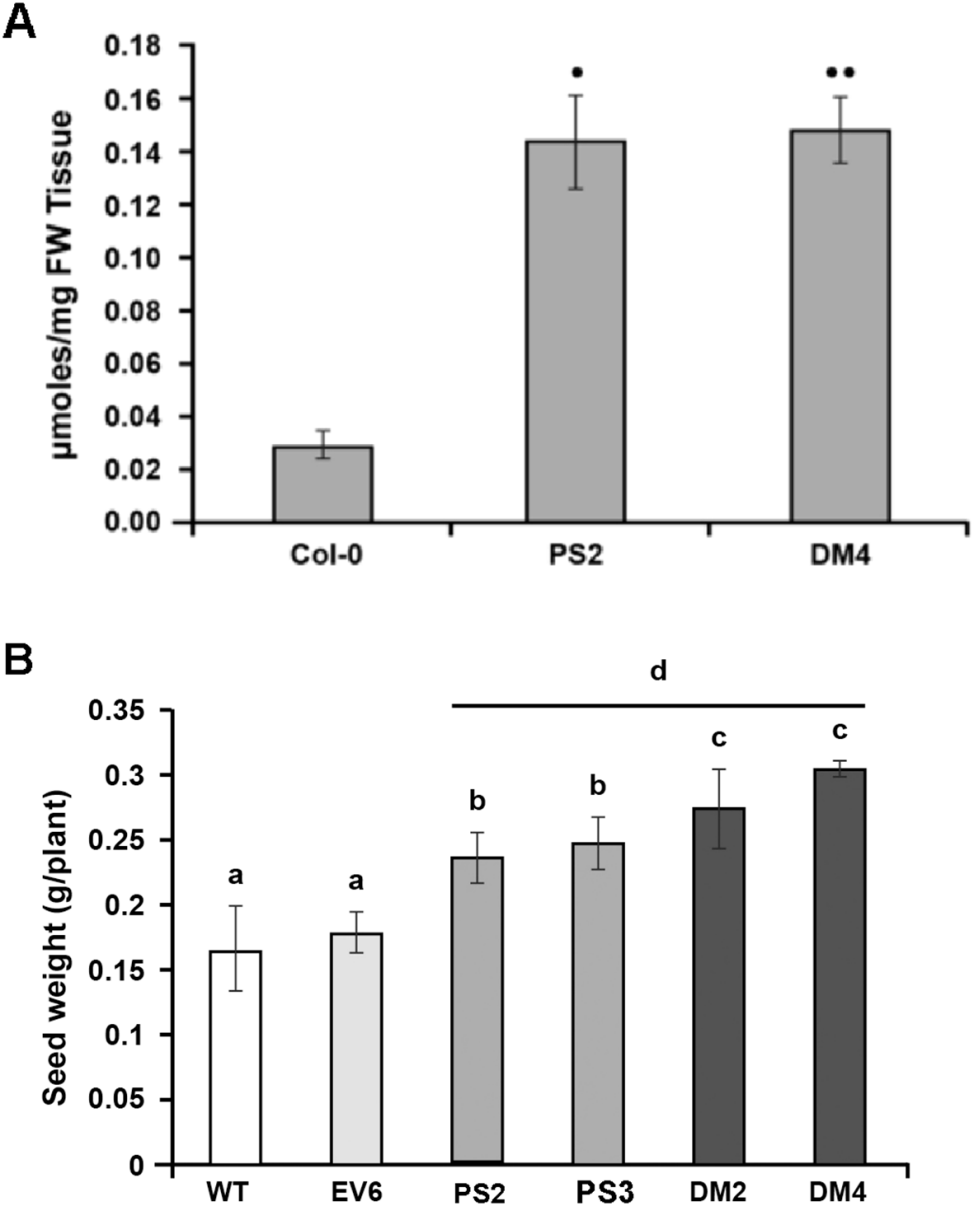
*PS* and *DM* overexpression significantly enhances the phosphate (Pi) content and seed yield of Arabidopsis seedlings. **(A)** Total Pi contents of 7-d-old Arabidopsis *PS2* and *DM4* transgenic lines, compared with WT Col-0 seedlings. Data are for means ± SE for two independent experiments, each with 3-5 biological replicates (n=10-20 seedlings). Significant differences (*p = 0.09, **p<0.05) were calculated using the Student’s *t*-test. **(B)** Comparison of seed weight (g) per plant of wild type (WT), empty vector (*EV*), *PS-* and *DM*-overexpressing Arabidopsis plants grown in greenhouses. Different letters above the bars indicate that the mean value was significantly different (p ¾ 0.05) from that of other samples when analyzed by twoway ANOVA (Bonferroni post-tests; n ≥ 16).

Measurement of total Pi in 7-d-old whole seedlings of Arabidopsis grown on MS medium showed that Pi content was higher in transgenic lines than in WT. Total Pi contents measured in *DM*-expressing (*DM4*) and in *PS-*expressing (*PS2*) (**Figure 4A**) were approximately 5-fold higher than in WT seedlings. The PS results are consistent with those of Thomas et al. (1999), who assayed a different *PS*-expressing line of Arabidopsis and reported that it took up more phosphate than WT.

If the Pi content of the medium is only half-strength MS, and it is allowed to deplete during growth of Arabidopsis through 12 days, then differences in seedling Pi content between Col-0 and the *PS*- and *DM*-expressing plants is eliminated (**Supplementary Figure S2B**), as was previously noted for PS and DM yeast growing in low Pi medium (**Supplementary Figure S2A**).

**Figure 5:**
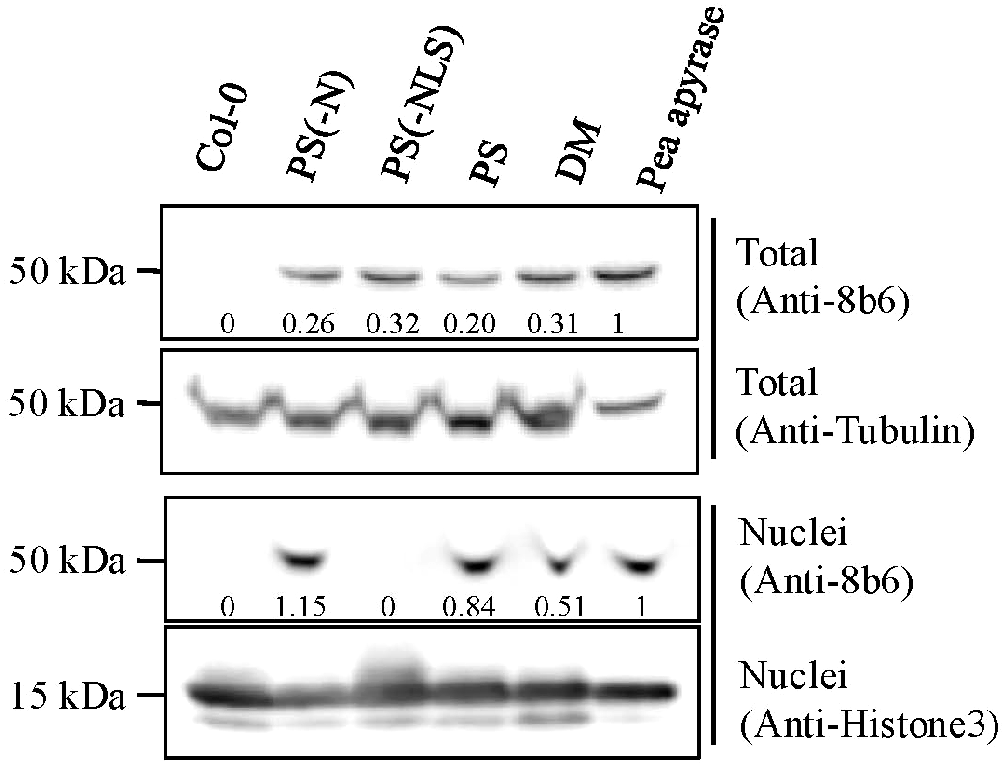
Immunoblot analysis of total extracts and of nuclei purified from etiolated Col-0 (WT) seedlings, and from etiolated Col-0 seedlings expressing PS (PS2), DM (DM4), or modified versions of PS missing either their N-terminal signal peptide (PS-N), or their nuclear localization sequence (PS-NLS). The positive control was highly purified pea apyrase. The immunostaining used apyrase-specific antibody (Anti-8B6) or Anti-histone3 antibody. Coomassie-Brilliant Blue (CBB) staining was used for the loading control. The 8b6 signal relative intensities, with Pea apyrase set as 1.0, are given beneath the stained bands.

Veerappa et al. (2019) described three independent Arabidopsis transgenic lines ectopically expressing *PS*, and the OE-2 and OE-3 lines from this study, were renamed *PS2* and *PS3* in this study. Expression of *DM* in putative lines was confirmed by RT-PCR analysis of *DM* and *nptII* transcripts (**Supplementary Figure S5**), using specific primers (**Supplementary Table S1**), and the *DM2* and *DM4* lines were used for this study. The *PS2* and *DM4* Arabidopsis transgenic lines were tested for PS and DM protein levels by immunoblots and showed similar levels in total protein extracts (**Figure 5**, DM and PS lanes). qRT-PCR analyses showed that the transcript level of *PS* in *PS2* is comparable but higher than the transcript level of *DM* in *DM4* (**Supplementary Figure S6**). *PS2* and *DM4* and two additional expression lines all had a significantly higher seed yield as compared to their respective EV and WT control plants (**Figure 4B**). When compared with *PS*-expressing plants, both lines expressing *DM* also had a small, but significantly higher total seed weight (**Figure 4B**).

### Both PS and DM co-purify with nuclei and with chromatin-associated proteins from yeast

**Figure 6:**
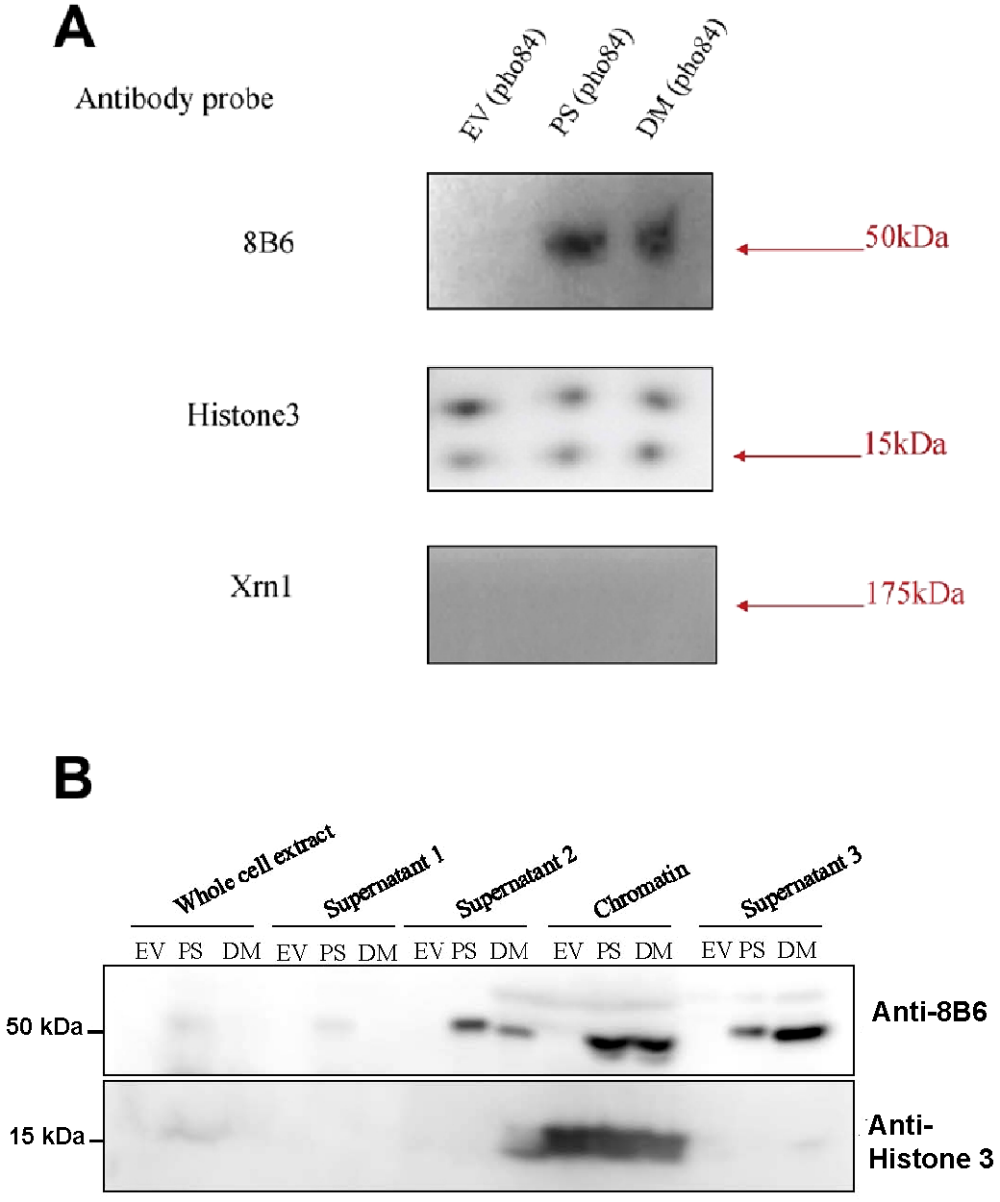
Immunoblots showing the presence of PS and DM in yeast nuclei and in yeast extracts. **(A)** nuclei purified from the NS219 mutant transformed with either an empty vector (EV), with *PS*, or with *DM*. **(B)** yeast extracts at different stages of the purification of chromatin-associated proteins from yeast transformed with either an empty vector, with *PS*, or with *DM.* These stages included whole cell extract, chromatin supernatant 1, chromatin supernatant 2, the final chromatin pellet, and the supernatant of this pellet (Supernatant 3). Histone 3 antibody was used as a positive control for nuclear expression, and exoribonuclease Xrn1 antibody was used as a negative control for cytoplasmic expression.

In immunoblot analyses of the proteins extracted both from highly purified preparations of yeast nuclei, and from preparations of nuclear proteins that are chromatin-associated, both PS and DM were immunostained by the highly specific monoclonal antibody 8B6 (Sabharwal et al., 2022) (**Figures 6A, B**). Purified pea apyrase was used as the positive control, and proteins extracted from yeast transfected with an empty vector were used as the negative control.

As judged by histone immunostaining, the quantity of nuclear proteins loaded in the empty vector (EV), and PS and DM lanes were about equal. There was no pea apyrase in the lane loaded with extracts of nuclei purified from yeast transformed with an EV (**Figure 6A**). As judged by immunostaining with an antibody raised to exoribonuclease Xrn1, a 175 kDa protein known to be cytoplasmic in yeast (Medina et al., 2014), there was little or no contamination of the nuclear protein preparation by cytoplasmic proteins (**Figure 6A**). Although at earlier steps in the purification, supernatant 2 and 3 levels of PS and DM were different, their levels in the final chromatin fraction were about equal (**Figure 6B**).

The chromatin-associated proteins in the preparation were identified by mass spectroscopy (MS). After applying the two MS filters described in the yChEF method (FDR of 1%, and for each protein the identification of at least two peptides in at least two out of three replicates), 2,379 different proteins were identified, including many well-characterized chromatin proteins in yeast (**Supplementary S3A**). The mass spectra data analysis also revealed that multiple different specific PS peptides were identified in chromatin samples isolated from both PS- and DMexpressing yeast (because PS and DM share > 99% sequence identity), but no PS peptides were identified in chromatin samples isolated from EV-expressing yeast (**Supplementary Table S3B**). These results confirmed immunoblot data.

We used the GENEONTOLOGY (http://geneontology.org/) tool and the *Saccharomyces* Genome Data base (https://www.yeastgenome.org) resources to classify the subcellular locations of proteins identified by MS in the preparation of chromatin-associated proteins. Over 64% were proteins known to be in the chromatin proteome of *S. cerevisiae* or known to function in nuclei. (e.g., **Supplementary Table S3**). About 16% were identified as primarily mitochondrial, which are typically the major contaminants in preparations of chromatin-associated yeast proteins (Cuevas-Bermúdez et al., 2019). The remaining 20% were cytoplasmic, cytoskeletal, membrane-associated, had unknown subcellular locales, or were broadly distributed among diverse membrane locales. Calmodulin, was included in this last category (data not shown), but it can functionally associate with chromatin-binding proteins, such as transcription factors (Abdel-Hameed et al., 2024). PS and DM were present in chromatin proteins isolated from cells expressing them, but not in chromatin proteins isolated from EV-expressing cells. These results indicated that PS and DM are not only expressed in the yeast nucleus but are also present in preparations enriched for chromatin proteins.

### PS and DM co-purify with nuclei in transgenic Arabidopsis seedlings

Confirmation of *PS* expression in transgenic lines of Arabidopsis was previously published (Veerappa et al., 2019). Several independent Arabidopsis lines (T3, homozygous) expressing the *DM* transgene were generated in parallel, and DM expression was confirmed by RT-PCR analysis (**Supplementary Figure S5**) using DM-specific primers. As assayed by immunoblots with the monoclonal antibody 8B6 (Sabharwal et al., 2022), both PS and DM co-purified with nuclei isolated from Arabidopsis Col-0 lines expressing full-length versions of these enzymes (**Figure 5**, DM and PS lanes).

Hsieh et al. (1996) identified a potential nuclear localization signal (NLS) in the primary structure of PS. After the removal of this bi-partite NLS, the modified enzyme was no longer detected in purified preparations of nuclei from transgenic seedlings that expressed it (**Figure 5**, PS-NLS lane), even though it was still present and intact in the crude extracts of these seedlings, from which the nuclei were purified (**Figure 5**). Removal of the enzyme’s N-terminal signal peptide (Hsieh et al., 1996), did not block its co-purification with nuclei of transgenic seedlings expressing this modification (**Figure 5**, PS-N lane).

### In transgenic yeast and Arabidopsis, PS and DM overexpression produce different transcriptome-level gene expression profiles

In transgenic yeast, RNA-seq analyses revealed significant differences between PS-expressing and DM-expressing lines when their gene expression profiles were compared to those of EVexpressing lines (**Supplementary Table S4A**). In *DM*-expressing, but not in *PS-*expressing yeast, a number of genes important for iron acquisition (*SIT1, FIT1, FET3*) were significantly induced (**Supplementary Table S4A**). For multiple yeast genes, RNA-seq data were verified by qRT-PCR to be differentially expressed (**Supplementary Table S5**).

In PS yeast, a large number of genes involved in the regulation of P homeostasis, including genes involved in the synthesis of InsPP signaling molecules that regulate SPX-domain proteins (INPHORS pathway; Austin and Mayer, 2020), are coordinately up-regulated (**Supplementary Table S4B**). Most are not DE in DM yeast. This gene expression profile, relative to the EV control, or DM cells, is typical of P-starved cells, although all cells were P-limited (0.1 mM Pi medium, *pho84* background) and Pi contents of EV, PS and DM lines grown for 48 hr in this medium are similar (**Supplementary Figure S2A**).

In 7-day-old Arabidopsis seedlings, significant differences in gene expression profiles were observed in *PS* and *DM* lines, compared with expression in WT seedlings (**Supplementary Table S6**). For multiple Arabidopsis genes, RNA-seq data were verified by qRT-PCR to be differentially expressed (**Supplementary Table S7**).

Genes responsive to Pi limitation were statistically overrepresented in *DM* but not PS seedlings (**Supplementary Figure S7**), but genes involved in Pi acquisition, transport, regulation of cellular Pi homeostasis and phospholipid remodeling, a well-known Pi starvation response (Misson et al., 2005), were DE in both *PS* and *DM* seedlings (**Supplementary Table S6**). This response was unexpected since the seedlings did not experience Pi limitation during growth. Repression of high-affinity Pi transporters PHT1;2 and PHT1;9 in PS seedlings might result from the elevated Pi contents of these seedlings (**Figure 4A**). In DM seedlings, *PHO1*, which is responsible for most root-to-shoot Pi translocation and is repressed by high shoot Pi contents (Gu et al., 2016) may also be a response to higher Pi contents in these plants. However, repression of *PHO2*, which would result in decreased proteolytic turnover of PHT1 and PHO1 transporter proteins and increased Pi uptake (Liu et al., 2012) may help explain enhanced Pi uptake by *DM* seedlings.

Venn diagram representation of differentially-expressed genes (DEG) in yeast (**Supplementary Figure S7A**) and Arabidopsis (**Supplementary Figure S7B**) reveal relatively little overlap between sets of induced or repressed genes in *PS* and *DM* lines. An analysis of GO BioProcess categories that are enriched among sets of genes that are uniquely DE in *PS* or *DM*, or among genes that are DE in both, identified processes and genes that are regulated in response to transgene expression.

### ChIP-seq identification of PS and DM binding sites in yeast chromatin

**Figure 7:**
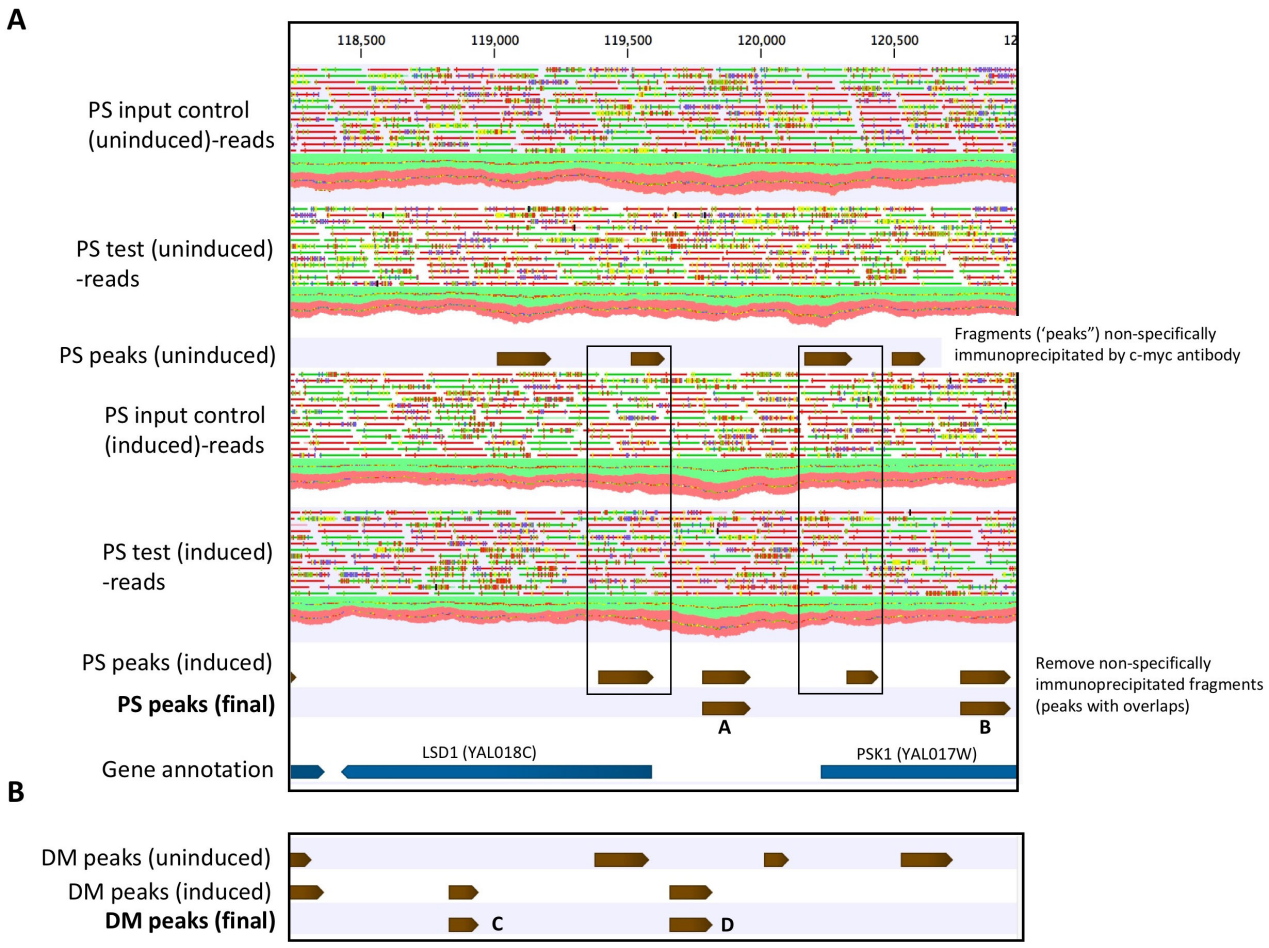
ChIP-seq identification of PS and DM binding sites in a region of yeast chromosome I. Mapped single-end reads (green,+ strand; red, - strand) coverage along the reference genome is shown. **(A)** Peak calling in the uninduced and induced PS samples, based on the distribution of unique reads mapped to the reference genome (normalized genome coverage) in test and input control samples. Peaks in the uninduced test samples represent non-specifically immunoprecipitated DNA fragments. Overlapping peaks in uninduced and induced test samples are removed and putative PS binding sites are retained. Gene annotation shows the locations of PS binding sites, relative to genes. PS peak “A” is located in a region of divergent promoters for upstream gene LSD1 (antisense strand) and downstream gene PSK1 (sense strand). PS peak “B” is located with the PSK1 gene and is > 1,000 bp upstream from the TSS for LSD1. It would not be classified as a potential regulatory site for LSD1. **(B)** Identification of DM peaks in the same region of chromosome I, using the approach described in part A. Mapped reads tracks are not shown. DM peak “C” is located within gene LSD1, > 1,000 bo from the TSS of downstream gene PSK1. In contrast, DM peak “D”, like PS peak “A”, lies within divergent promoters for LSD1 and PSK1 and partially overlaps with the putative PS “A” binding site. High-confidence (p ≤ 0.05) final peak assignments for PS and DM are shown in tracks. Peaks of varying lengths are represented by brown arrows. Mapped single-end reads are green (+ strand) or red (- strand).

The general approach used to identify PS and DM binding sites (peaks) in a region of yeast chromosome I is illustrated in **Figure 7**. Overlap analyses further identified peaks that were PS- or DM-specific, or which occurred in both PS and DM samples. The ChIP-seq assay identified 4,706 and 3,433 unique binding sites in PS and DM yeast genomic DNA, respectively. The sites occurred nearly equally in genes (43.2%) and intergenic regions (56.8%). Peak overlap analyses identified 3,903 sites that occurred only in the induced, PS-expressing yeast samples. Similarly, 2,779 DM-specific sites, and 803 sites that overlapped in PS and DM tracks, were identified.

**Figure 8:**
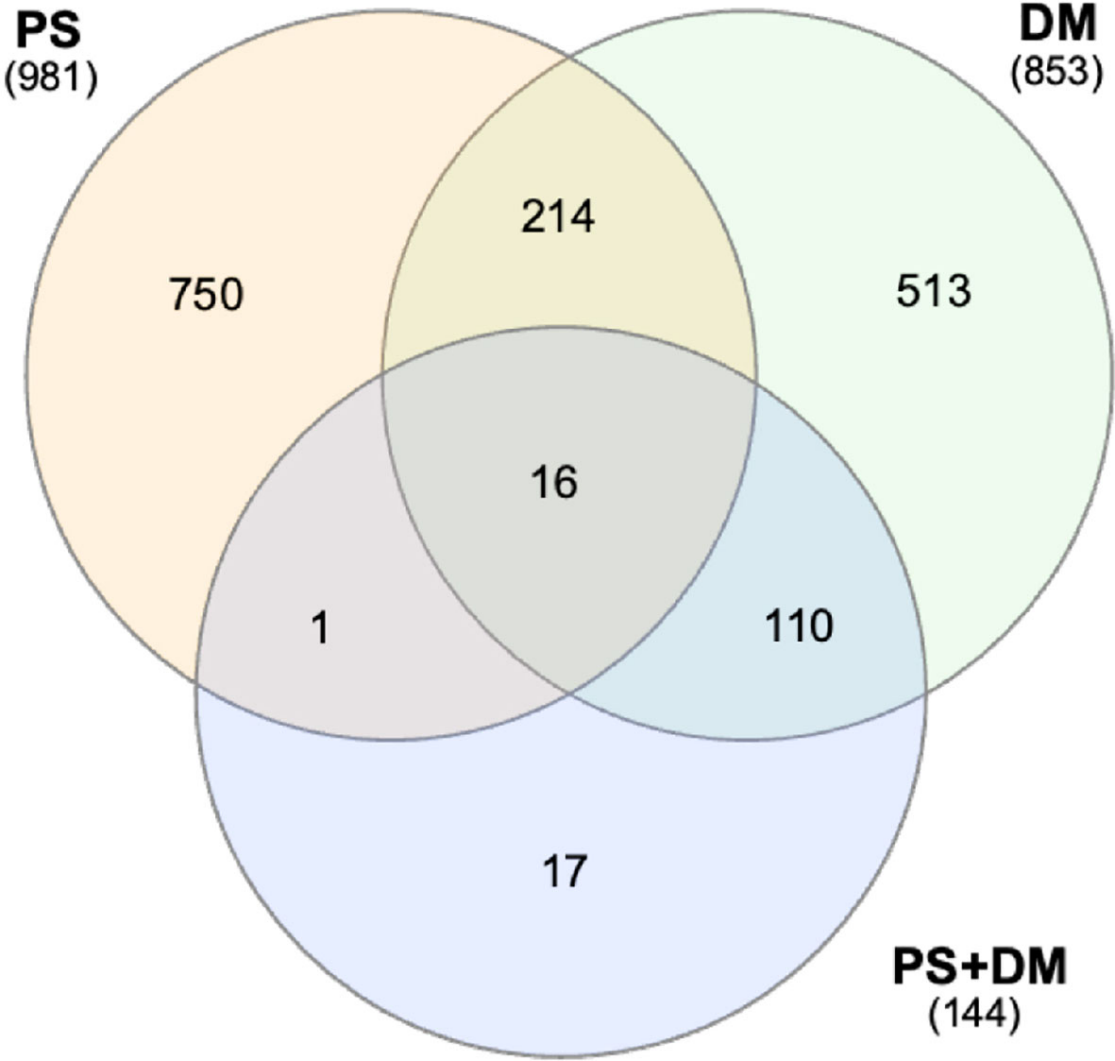
Venn analysis of potentially-regulated target genes with PS-specific, DM-specific, or PS+DM shared binding sites in their promoters. The large number of genes in the PS+DM overlap group with DM is due to the selection of DM as the reference (see **Figure 7C**). Numbers of DEG included in PS-specific or DM-specific target cohorts. Total numbers of DEG are indicated in parentheses.

We explored potential functional binding events by integrating ChIP-seq and gene expression data for the PS and DM lines. Some examples of this integration are shown in **Supplementary Table S8**. Further analyses were limited to putative promoter binding sites (-1000 bp from transcription start sites, TSS) of potential target genes (shortest upstream antisense and downstream sense strand, relative to the binding site) that were also DE in PS or DM yeast. Thus, 545 PS-specific sites were located in promoters of 981 DEG, 468 DM-specific sites (853 DEG), and 74 overlapping PS+DM sites (144 DEG) were further characterized. Between 1935% of the binding sites were located within divergent promoters, with the potential to regulate both upstream and downstream targets. There was relatively little overlap between potential PS and DM target gene sets (**Figure 8**).

The narrow footprint for PS and DM binding sites (mean = 205 bp and 202 bp, respectively) is typical of TF binding sites (Hughes and de Boer, 2013). Although PS and DM sites were distributed throughout the 1000 bp putative promoter regions, a higher percentage of DM sites were located closer to the TSS (**Supplementary Figure S8**).

### Identification of enriched motifs in PS and DM binding site sequences

Motifs for yeast transcription factors were identified in PS and DM binding sites in promoters of DEG. The frequencies with which each motif sequence was represented in these sites is shown in **Supplementary Table S9**. In general, motif sequences in PS binding sites were different from those in DM binding sites. Only the Abf1p motif was shared among PS and DM sites and its frequency was approximately 5-fold higher in PS sites. There was relatively little enrichment of most motifs.

A more comprehensive motif enrichment analysis, including discovery of new motifs, was carried out using XSTREME (https://meme-suite.org/meme/tools/xstreme). The Tomtom motif comparison tool was used to identify yeast TF motifs that matched the consensus sequences for any discovered motifs. The results are summarized in **Supplementary Table S10**. Similar 15 bp Azf1p (zinc-finger) motifs were identified in PS-specific and DM-specific sites, as well as sites bound by both PS and DM, although it occurred with lower frequency in PS sites. An unusual Rfx1p-like motif with repeating GCA elements and two enriched Abf1p motifs were also enriched.

**Figure 9:**
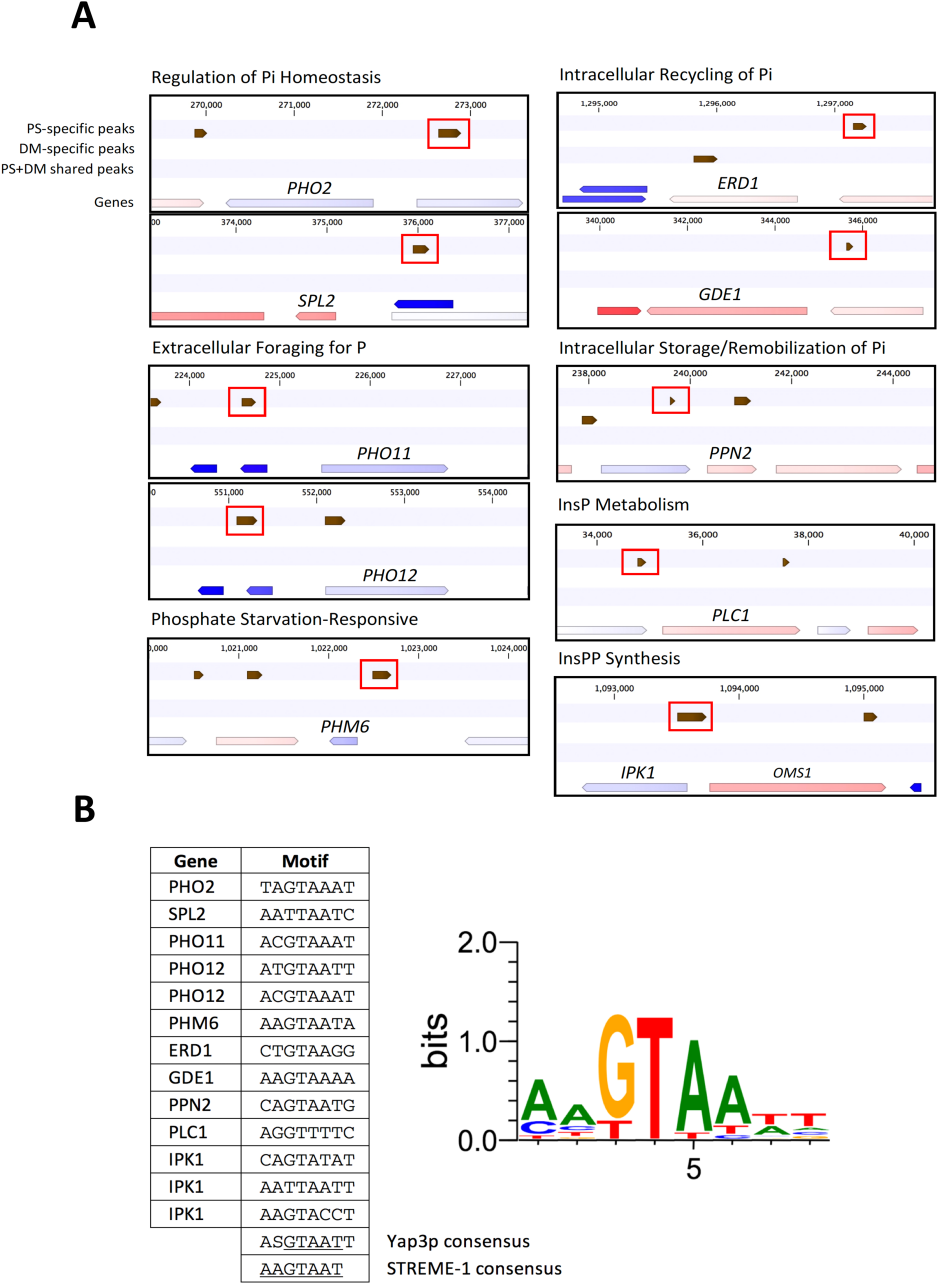
Discovery of a Yap3p-like motif in PS-specific binding sites in promoters of phosphate metabolism genes induced in PS yeast. **(A)** Locations of PS and DM binding sites and potentially-regulated target genes. PS-specific binding sites in promoters are enclosed in red boxes. **(B)** Yap3p-like motifs within PS binding sites and Logo representation of their sequences, compared with the discovered STREME-1 motif and Yap3p motif consensus sequences.

Ten of the induced P metabolism genes in PS yeast (**Supplementary Table S4B**) also had PSspecific binding sites in their promoters. Gene models and the locations of their PS binding sites is shown in **Figure 9A**. Genes in the *PHO* regulon are transcriptionally activated by Pho4p, and a second TF Pho2p, which cooperatively binds Pho4p at CACGTK motifs (K = G/T; Zhou and O’Shea, 2011). The CACGTK motif was not enriched in PS binding sites and occurs only in sites located in SPL2 and PHM6 promoters. This suggests that direct PS interaction with the CACGTK motif, or bound Pho4p, is not likely to explain the induction of these Pi metabolism genes. Although the exact relationship between PS binding and induction of PHM6 expression will require further functional characterization, enhanced PHM6 expression may contribute to the increased Pi contents of PS yeast cells, because a recent report suggests PHM6 functions in concert with Pi transporters to provide increased Pi uptake under phosphate surplus conditions in yeast (Kulakovskaya et al., 2023). Thus, the increased PHM6 expression in PS yeast might help explain the increased Pi content of PS yeast grown in 1 mM phosphate (**Figure 3**), in contrast to no increase in Pi content observed when PS yeast is grown in 100 µM Pi (**Supplementary Figure S2A**). Further analyses of the PS-specific binding site sequences for the ten P metabolism genes identified one to three Yap3p-like motifs in each site (**Figure 9B**), suggesting functional interactions between PS and Yap3p or its binding sites.

## Discussion

Changing just two amino acids in the primary structure of PS generated a modified version, DM, whose transgenic expression in yeast and several different plants induced largely different transcriptome-level gene expression responses. In parallel, DM expression also increased the Pi uptake and growth of these organisms, relative to PS lines. These directed structural changes had the potential to result in differences in how the DM protein interacted with both CaM and DNA.

The S208L and P216R mutations that were introduced into the PCBD1 sequence of PS produced a more amphipathic PCBD1-DM peptide structure similar to that of PCBD2 and provided a second site that could potentially bind CaM in DM. The presence of more than one CaM binding site on a protein is not unusual (Tidow and Nissen, 2013; Andrews et al., 2021). However, CaMELS structural analysis predicted that in the intact DM protein PCBD1 was unlikely to bind CaM, and, in accord with this prediction, a biotinylated-CaM assay revealed that DM did not bind more CaM than PS. These results made it likely that the positive effects of DM on growth and Pi content were more likely due to changes in its nuclear activity rather than to changes in its responsiveness to CaM.

Partially purified PS and DM from yeast were both only minimally stimulated by CaM, in contrast to highly-purified PS isolated from pea nuclei, which is stimulated 3-fold by CaM (Chen et al., 1987). The cause of this decreased response of the yeast PS and DM is unclear, but could plausibly be due to differences in post-translational modifications to PS and DM proteins expressed in yeast and pea.

Expression of *PS* and *DM* genes increases the Pi contents in yeast, hairy roots, and Arabidopsis, but only at relatively high (≥ 1 mM) [Pi]. In yeast, the *pho84* mutant has a K_m_ = 204 µM, compared with a K_m_ = 58 µM for WT cells (Wykoff and O’Shea, 2001), thus all transgenic *pho84* lines would be P-limited in Pi depleted medium, and Pi uptake was enhanced in PS and DM yeast only at relatively high [Pi]. Gene expression data for PS and DM yeast grown in 0.1 mM Pi medium show an induction of a large number of phosphate starvation response genes in PS cells and, to a lesser extent, in DM cells, consistent with P-limitation in these cultures. Perhaps, this Pi-starvation phenotype, relative to the EV control, results in enhanced Pi uptake when the Pi content of the medium is increased.

Similarly, in Arabidopsis PS seedlings have an increased K_m_ for Pi uptake, and at [Pi] ≥ 2 mM, seedling Pi contents and growth were significantly increased, relative to WT seedlings (Thomas et al., 1999). In the present study, PS and DM seedlings grown in Pi-depleted medium had similar growth and Pi contents, but both PS and DM seedlings grown in Pi-sufficient medium had ~5-fold higher Pi contents than controls. Similar results were obtained for several independent lines of hairy root cultures of corn, canola and soybean grown in 1 mM Pi medium.

Structural analyses predicted a likely NLS sequence in PS separate from its DNA-binding domain, and the removal of this sequence from PS blocked its functional potential to increase the growth of the yeast NS219 mutant. These results made it likely that the effects of PS expression on the growth of this yeast were due to its activity in the nucleus. As a more rigorous test of this conclusion, follow-up experiments could investigate whether restricted point mutations in the NLS of PS also suppressed its ability to enhance the growth of NS219 yeast when expressed in this mutant. Although parallel experiments with DM-NLS were not carried out, it seems likely that nuclear targeting of this protein is also required for the enhanced growth and Pi accumulation phenotype in DM yeast and plants.

The expression of PS and DM in nuclei has different effects on gene expression. These differences could be due in part to the higher specific activity of PS over that of DM, because the resultant differences in the concentration of ATP in the nucleus could affect nuclear RNA splicing (Ali and Reddy, 2006). In Arabidopsis, these differences could also be influenced by a dosage effect, since Arabidopsis nuclei accumulate relatively more PS than DM (**Figure 5**) (Li et al., 2019). A third possible explanation for the different gene expression profiles in *PS*- and *DM*expressing yeast and Arabidopsis, relative to controls, is that these proteins function as transcription factors. PS and DM may recognize different DNA binding sites, or may associate with different chromatin proteins to regulate transcription. At these different binding sites, different NTPDase activities of PS and DM could also impact gene expression, just as the ATPase activity of the AfsR transcription factor helps regulate gene expression in *Streptomyces coelicolor* (Lee et al., 2002).

To test whether PS and DM have different chromatin binding sites in yeast nuclei, ChIP-seq analyses were carried out. These analyses confirmed distinct subsets of PS- and DM-specific binding sites in yeast chromatin. Within promoter binding sites, DM binding occurs closer to the transcription start sites than for PS binding sites, and enrichment of different TF motifs within PS and DM sites further support different types of interactions with yeast chromatin. For most differentially expressed yeast genes, PS- and DM-specific gene expression was associated with PS- and DM-specific binding sites, and there was little overlap between potentially-regulated target gene sets. These results suggest that at least a subset of binding events is functional and are consistent with the hypothesis that the differential effects of *PS* and *DM* expression in yeast are mediated, at least in part, by their differential binding to and activity on chromatin.

A subset of DEG have overlapping PS and DM binding sites, and many genes are DE in both PS and DM yeasts, even where only a single PS or DM binding site has been identified in ChIP-seq assays. This is not surprising, given the overall structural homology of the PS and DM proteins. Both PS and DM binding sites are enriched for the Azf1p motif. Azf1p is a glucose-dependent activator of many genes involved in growth, C-metabolism and cell wall maintenance in yeast (Newcomb et al., 2002), and the enhanced growth of both PS and DM yeast may result, in part, from interactions with this transcription factor. Electrophoretic mobility shift assays (EMSA) will be required to further characterize PS and DM binding to specific DNA sequences, such as Azf1p or Yap3p motifs. Regarding the Yap3p motifs, since they were the only ones conserved in all 10 of the PS binding sites, we speculate that PS could interact directly with the Yap3p motifs, or, indirectly, with the Yap3p transcription factor or other proteins occupying those sites. In this way the binding of PS to PHO promoters could influence gene expression. EMSA assays would provide a key test this hypothesis.

Another important follow-up experiment would be an Arabidopsis ChIP-seq assay. This assay would reveal whether differences in gene expression observed in *PS*- and *DM*-expressing seedlings are correlated with different PS and DM chromatin binding sites in this plant. They would also resolve whether there are any common features in the chromatin sites of yeast and Arabidopsis to which PS and DM bind.

In Arabidopsis, expression of only a few phosphate-starvation responsive genes was changed in response to elevated Pi contents in 7-d-old *PS*- and *DM*-expressing seedlings. In both PS and DM seedlings, however, genes that regulate root system architecture (RSA) in response to altered Pi availability were induced, consistent with the observed expanded RSA for these seedlings growing on Pi-sufficient media, as was previously reported by Veerappa et al. (2019) for PS-expressing Arabidopsis. This developmental response may contribute to the marked increase in Pi contents of PS and DM plants, since roots are the site of Pi uptake into the plant.

It is clear that the growth enhancement of PS and DM yeast grown in 0.1 mM Pi is not related to enhanced Pi uptake, since Pi contents of these lines did not differ from control cells. Observed changes in gene expression in these cells could impact many other processes related to growth. For example, the strong induction of *FIT2, FET3* and *SIT1* genes that promote iron uptake critical for iron nutrition (Haurie et al., 2003; Ramos-Alonso et al., 2020) could help explain the enhanced growth of the *DM-*expressing over that of *PS*-expressing yeast.

Similarly, the phenotypic differences between WT Arabidopsis plants and those expressing *DM* and *PS* likely resulted from differences in gene expression in these lines. Enhanced growth and seed yields may result from induction of a cytostatin (Balbinott and Margis, 2022), the GAresponsive GAST1 protein (Li et al., 2022), the UPBEAT transcription factor (Wells et al., 2010), the ethylene-responsive factor RAP2-3 (León et al., 2020), and the CCT-domain protein, also referred to as “FITNESS” (Osella et al., 2018). This FITNESS protein attenuates the JA signaling cascade (Krishnamurthy et al., 2021), shifting allocation of resources from defense to growth and promotes reproductive fitness (Guo et al., 2018), consistent with observed increased seed yields in PS and DM seedlings.

Our observation that 7-d-old *PS-* or *DM*-expressing Col-0 seedlings that are grown continuously in MS media at 1.25 mM Pi have a 5-fold higher Pi content compared to wild-type plants is very similar to that of Thomas et al. (1999), who observed increased Pi content in 15-d-old Arabidopsis plants expressing *PS* compared to Ws wild-type plants. This indicates that when seedlings are grown at high Pi, the increased Pi accumulation promoted by the transgenic expression of *PS* is likely to persist past one week and is not dependent on the specific ecotype in which it is expressed.

The PS enzyme was originally purified from a chromatin sub-fraction of purified nuclei from peas, and it was shown to selectively bind to a DNA-affinity column (Chen, 1987). Mutations introduced into DM did not change the nuclear localization signal (NLS) of PS that could direct it to the nucleus. Thus, DM would be as likely as PS to co-purify with nuclei and with chromatin proteins isolated from transgenic yeast and Arabidopsis. Our results confirmed this prediction and indicated that the NLS and affinity for chromatin of DM and PS are operative in radically different cellular environments.

Although the preceding discussion emphasizes the nuclear roles of PS and DM, both also have a signal peptide, and their role in the ECM could also contribute importantly to the Pi content changes observed in hairy roots and Arabidopsis. Like GS52 in soybeans (Tanaka et al., 2011), PS can function as an ectoapyrase in the ECM (Thomas et al., 1999; Clark et al., 2021), potentially regulating eATP levels and purinergic signaling (Clark et al., 2014; Matthus et al., 2022), or participating in salvaging of apoplastic NTP/NDP pools (Riewe et al., 2008; Slocum et al., 2023). Such salvaging could increase Pi content in both hairy roots and Arabidopsis, although we have not tested whether PS and DM differ in their ability to harvest Pi from extracellular nucleotides.

In Arabidopsis, differences in the ECM function of AtAPY1, AtAPY2, and AtAPY7 could impact levels of eATP (Clark et al., 2021; Gupta et al., 2024), which, in turn, would impact gene expression changes (Lim et al., 2014; Zhu et al., 2020). Whether DM and PS need to function in both nuclear and ECM domains to promote the physiological and growth responses observed in yeast, hairy roots and Arabidopsis remains to be determined.

## Data Availability

The datasets presented in this study can be found in online repositories. The names of the repository/repositories and accession number(s) can be found in the article/**Supplementary Material**.
