# Supplementary material for: Modified pea apyrase has altered nuclear functions and enhances the growth of yeast and Arabidopsis
Source: Front Plant Sci. 2025 May 26;16:1584871. doi: 10.3389/fpls.2025.1584871 (PMC12146327; doi:10.3389/fpls.2025.1584871)
Supplement: Supplementary file 1 [file DataSheet1.pdf]

**Supplementary Table S1.** PCR primers used in this study.	
**PCBD1 Mutagenic Primers Sequence (5'-->3')**	
Forward (S208L); NTP9-lst Mut-F	CAAATGGCGTATGCAGTATTAAAGAAAACTGC
Reverse (S208L); NTP9-lst Mut-R	CATTTTTAGCAGTTTTCTTTAATACTGCATACGC
Forward (P216R); NTP9-2nd Mut-F	GAAAACTGCTAAAAATGCTCGAAAAGTTGCAG
Reverse (P216R); NTP9-2nd Mut-R	CTCCATCTGCAACTTTTCGAGCATTTTTAGC
**psNTP9 N-Terminal Deletion Primers**	
NTP9-F-N	GAAATTTCCTCTTACGCTGTAGTATTC
NTP9-R	TTAAACAAAATACATCAATC
**psNTP9 NLS Deletion Primers**	
NTP9-F	ATGGAGCTCCTTATTAAACTTATCAC
NTP9-R	TTAAACAAAATACATCAATC
NTP9-NLS-NR	GATCATATGGTATTCCTGATACTGCATACGCC
NTP9-NLS-CF	GGCGTATGCAGTATCAGGAATACCATATGATC
**13Myc-tagged PS and DM Expression Constructs**	
HW1	ATCGATCGACCGCGGGGAGGTGGAGGTTCACCCGGGTTA ATTAACGGT
HW2	AATCACTAGTGAATTCGCGCGTCGACTCGATCGAT
HW3	ATCGATCGAGTCGACTCATGTAATTAGTTATGTCACGCT
HW4	ATTTGAGCGATTGATGTATTTTGTTCCGCGGTCGATCGAT
**psNTP9/spNTP9-DM Specific Primers**	
PS, DM-F	GCTCCAAAAGTTGCAGATGG
PS, DM-R	CCGAAGTGTAAGTA ACTGTGA
**npt/1 Specific Primers**	
nptll-F	ATGATTGAACAAGATGGATTGC
nptll-R	TCAGAAGAACTCGTCAAGAAG
**psNTP9/psNTP9-DM Specific qRT-PCR Primers**	
NTP9/DM-F	ATGGAGCTCCTTATTAAACTTATCAC
NTP9/DM-R	TTAAACAAAATACATCAATCGCTCAA
Actin-F	CCCGCTATGTATGTCGC
Actin-R	AAGGTCAAGACGGAGGAT
PP2A-F	GTTGTGTGAGCACGCAAAGA
PP2A-R	GTTGTGTGAGCACGCAAAGA
DTX3 V2-F	CCCATTCAGAACTGCTGTGAAAC
DTX3 V2-R	CACTTCTCTTCACATGCCGA
UPB1-F	AAGACAAAGGGCTCGAAGGG
UPB1-R	TGTATTGCGCGACAAGGTCT
UGT74E2-F	GTGGAAGGTTGGGGTAAGGG
UGT74E2-R	TGGACTTGCAAAAGACGATGT
PHO2-F	GCTCAGTCCCAGGTCACAAA
PHO2-R	TCGTGATATGGCGTTCCAGG

AT1G75945-F	TCGTTTTTGGCTCTCTGCCT
AT1G75945-R	TGCCCTACGAGACCCTAAGA
DAL5-F	AAGACTGGCTGGTGCTTACC
DAL5-R	AAGGTTTGTGGTCCTGCCAA
ALG9-F	CACGGATAGTGGCTTTGGTGAACAATTAC
ALG9-R	TATGATTATCTGGCAGCAGGAAAGAACTTGGG
FIT2-F	TGGTCACGGACTTGACGAAG
FIT2-R	ACGGTCATTTCGTGGTCCTC
AGP1-F	TGGCGCTGGTAATGATGGTT
AGP1-R	ATCATTTGTTTGGCCGCACC
ASP3-1-F	GTTCCTCCAGATGATGCCCC
ASP3-1-R	CCGTAGCCGGAGTACAAACA
VTC3-F	TGTTTTGCGGAGTATGGGCT
VTC3-R	AACGAAGGCTGGGAGGAAAC
GIT1-F	GCGTCGAATGTTTCCAACCC
GIT1-R	CCCATCTTACCAGTCCAGCC
GDE1-F	ACGGTTGGTGCAAGTGGTTA
GDE1-R	CACTATCGACGATCACGGCA

Supplementary Table S2. Enzymatic characterization of partially-purified PS and DM proteins extracted from yeast. A) Specific activities with different adenylate substrates. B) Effects of Ca^{2+} and calmodulin (CaM) on activities. Activities were assayed in 60 mM Hepes buffer, pH 7.5, 3 mM $MgCl_2$, 3 mM substrate, incubated at 28°C for 16 min. Total protein: PS sample, 3.2 µg; DM sample 3.0. Data are means of two replicate assays in which the purities of PS and DM were >23%<29%, as estimated by specific activities, assuming >95% pure PS has a specific activity of 138 µmol Pi mg $protein^{-1}$ min^{-1} (Chen et al., 1987).

A

APY Assayed	Substrate	Specific Activity (µmole Pi/mg protein/min)
PS	ATP	75
	ADP	58
	AMP	<2.0
DM	ATP	47
	ADP	25.3
	AMP	<2.0

B

Treatment	Specific Activity (µmole Pi/mg protein/min)
-CaM	37.6
+CaM	41.2
+CaM+EGTA	36.5
-CaM	30.1
+CaM	32.4
+CaM+EGTA	29.3

Supplementary Table S3. Summary of PS peptides identified by MS in the chromatin-enriched samples of EV-, PS-, and DM-expressing NS219 yeast. (**A**) Peptides of known chromatin-associated proteins identified in chromatin-enriched fractions isolated from yeast expressing either EV, PS, or DM. (**B**) Identification of PS peptides in the chromatin fractions of PS- and DM-expressing yeast, but not in the chromatin-enriched fraction of EV-expressing yeast. Each of the peptides in the chromatin-enriched fractions isolated from PS- and DM-expressing yeast were identified multiple times by MS.

A

Complex	Proteins whose peptides were detected by MS
Histones	Histone H1, Histone H2AZ, HistoneH3, Histone H4
RNA polymerases	Rpa190, Rpa135, Rpa49, Rpa43, Rpa34, Rpb1, Rpb2, Rpc40, Rpc34, Rpc31, Rpc25, Rpc19, Rpc17
Replication factors	Rfa1, Rfc1, Rfc2, Rfc3, Clf4
Chromatin remodelers (RSC, SWI/SNF, SWR1, INO80)	Sth1, Rsc3, Rsc4, Rsc6, Rsc7, Rsc8, Rsc9, Rsc14, Rsc30, Sfh1, Npl6, Arp7, lsw1, Isw2, loc2, loc3, Swr1, Rvb1, Rvb2, Nhp10, les1, les3
Transcription Factors	TFB1, TAF4, TAF5, TAF8, TAF12, TAF14, TFC4, TFC6, TFC7, Spt6, Spn1

B

Identified PS peptides in EV sample	none
Identified PS peptides found in PS sample	QEEISSYAVVFDAGSTGSRIHVYHFNQNLDLLHIGK; ITPGLSSYANNPEQAAKSLIPLLEQAEDVVPDDLQPK; TVGVIDLGGGSVQMAYAVSK; GIPYDLYVHSYLHFGR; SPNPCLLAGFNGIYTYSGEEFKATAYTSGANFNK; NLFASSSFFYLPEDTGMVDASTPNFILRPVDIETKAKEACALNFEDAK; EIEYQDAIVEAAWPLGNAVEAISALPK
Identified PS peptides found in DM sample	ITPGLSSYANNPEQAAK; SPNPCLLAGFNGIYTYSGEEFKATAYTSGANFNK
Identified PS peptides in EV sample	None

Supplemental Table S3C. Mass-spectrometry identification of yeast chromatin proteins. Mitochondrial and "other" proteins are also listed.

COLOR CODE: Yellow, chromatin-associated proteins (1,291); orange, mitochondrial proteins (399); non-colored, other protein (689)

PROTEIN NAMES
ADP,ATP carrier protein 1
ARS-binding factor 1
Actin-binding protein
Acetyl-CoA carboxylase;Biotin carboxylase
Metallothionein expression activator
Acetyl-coenzyme A synthetase 2
Transcriptional adapter 2
Bifunctional purine biosynthesis protein ADE16;Phosphoribosylaminoimidazolecarboxamide formyltransferase;IMP cyclohydrolase
Bifunctional purine biosynthesis protein ADE17;Phosphoribosylaminoimidazolecarboxamide formyltransferase;IMP cyclohydrolase
Amidophosphoribosyltransferase
Phosphoribosylformylglycinamidine synthase
Adenylate kinase
Probable ATP-dependent permease
Regulatory protein ADR1
Accumulation of dyads protein 2
Iron-regulated transcriptional activator AFT1
ADP-ribosylation factor GTPase-activating protein effector protein 1
Hsp90 co-chaperone AHA1
Intron-encoded DNA endonuclease aI4;Truncated non-functional cytochrome oxidase 1;DNA endonuclease aI4
Probable oxidoreductase AIM17
Serine/threonine-protein kinase AKL1
Lysophospholipid acyltransferase
Dol-P-Man:Man(7)GlcNAc(2)-PP-Dol alpha-1,6-mannosyltransferase
UDP-N-acetylglucosamine transferase subunit ALG13
Dolichyl-phosphate beta-glucosyltransferase
Alpha-1,2-mannosyltransferase ALG9
Serine/threonine-protein kinase Haspin homolog ALK1
Serine/threonine-protein kinase Haspin homolog ALK2
Alpha-mannosidase
Protein APA1;5,5-P-1,P-4-tetraphosphate phosphorylase;ADP-sulfurylase
Vacuolar aminopeptidase 1
Aspartyl aminopeptidase 4
AP-1 complex subunit beta-1
AP-2 complex subunit alpha
AP-1 complex subunit gamma-1
AP-1 complex subunit mu-1-I
Adenine phosphoribosyltransferase 1
tRNA-aminoacylation cofactor ARC1
Actin-related protein 2/3 complex subunit 5
Actin-related protein 2/3 complex subunit 1
N-terminal acetyltransferase A complex catalytic subunit ARD1
Sterol O-acyltransferase 2
Pentafunctional AROM polypeptide;3-dehydroquinate synthase;3-phosphoshikimate 1-carboxyvinyltransferase;Shikimate kinase;3-d[illegible]
Aromatic amino acid aminotransferase 2
Actin-like protein ARP6
Actin-related protein 7
Actin-like protein ARP9
Probable metalloprotease ARX1
Guanine nucleotide-binding protein subunit beta-like protein

Activator of stress genes 1
Activator of SKN7 protein 10
Serine/threonine-protein kinase ATG1
Autophagy-related protein 19
Ammonia transport outward protein 3
NADPH-dependent 1-acyldihydroxyacetone phosphate reductase
Asparagine-rich zinc finger protein AZF1
Leu/Val/Ile amino-acid permease
Branched-chain-amino-acid aminotransferase, cytosolic
Myosin tail region-interacting protein MTI1
Spindle pole component BBP1
Serine/threonine-protein kinase BCK1/SLK1/SSP31
cAMP-dependent protein kinase regulatory subunit
Bromodomain-containing factor 1
Bromodomain-containing factor 2
Transcription factor TFIIIB component B
GTPase-activating protein BEM2/IPL2
Nuclear segregation protein BFR1
Protein BFR2
Nuclear fusion protein BIK1
Adenosylmethionine-8-amino-7-oxononanoate aminotransferase
Protein BMH1
Ribosome biogenesis protein BMS1
25S rRNA (uridine(2634)-N(3))-methyltransferase
Protein BOB1
Protein BOI2
UBP3-associated protein BRE5
Transcription factor IIIB 70 kDa subunit
Condensin complex subunit 2
Vacuolar-sorting protein BRO1
Pre-mRNA-splicing factor BRR1
Pre-mRNA-splicing helicase BRR2
Ribosome biogenesis protein BRX1
Protein BSP1
Bud site selection protein 21
Bud site selection protein 22
18S rRNA (guanine(1575)-N(7))-methyltransferase
Pre-mRNA-splicing factor BUD31
Ubiquitin ligase-binding protein BUL1
Negative cofactor 2 complex subunit alpha
Chromatin assembly factor 1 subunit p60
CCR4-NOT transcriptional complex subunit CAF120
Elongation factor 1-gamma 1
Centromere DNA-binding protein complex CBF3 subunit A
H/ACA ribonucleoprotein complex subunit 4
Serine/threonine-protein kinase CBK1
NADH-cytochrome b5 reductase 1
Cytochrome B translational activator protein CBS2
Glucose-repressible alcohol dehydrogenase transcriptional effector
T-complex protein 1 subunit beta
T-complex protein 1 subunit gamma
T-complex protein 1 subunit delta
T-complex protein 1 subunit zeta
Cell division control protein 1
Cell division control protein 12
Tyrosine-protein phosphatase CDC14

Cell division control protein 24
Cell division control protein 3
Eukaryotic translation initiation factor 4E
Hsp90 co-chaperone Cdc37
General negative regulator of transcription subunit 1
Cell division control protein 48
Cell cycle serine/threonine-protein kinase CDC5/MSD2
Leucine--tRNA ligase, cytoplasmic
Cell division control protein 73
DNA ligase 1
Phosphatidate cytidylyltransferase
Protein CFT1
Activatory protein CHA4
Clathrin heavy chain
Chromo domain-containing protein 1
ATP-dependent RNA helicase CHL1
CDP-diacylglycerol--serine O-phosphatidyltransferase
Phosphatidylethanolamine N-methyltransferase
Chitin biosynthesis protein CHS5
Proteasome-interacting protein CIC1
Casein kinase II subunit alpha
Casein kinase II subunit alpha
Casein kinase II subunit beta
Casein kinase II subunit beta
Serine/threonine-protein kinase CLA4
Serine/threonine-protein phosphatase 2B catalytic subunit A2
DNA damage-binding protein CMR1
Protein CMS1
Chaotic nuclear migration protein 67
Coatomer subunit alpha
Cobalt uptake protein COT1
Cytochrome c oxidase subunit 7A
Peptidyl-prolyl cis-trans isomerase
Peptidyl-prolyl cis-trans isomerase D
Peptidyl-prolyl cis-trans isomerase CPR6
Carboxypeptidase S
Probable S-adenosylmethionine-dependent methyltransferase CRG1
Cruciform DNA-recognizing protein 1;CRP1 short N-terminal subpeptide;CRP1 short C-terminal subpeptide
Transcriptional regulator CRZ1
Histone H3-like centromeric protein CSE4
Monopolin complex subunit CSM1
Cop9 signalosome complex subunit 12
Transcription factor CSR2
ATF/CREB activator 2
Chromosome stability protein 9
Chromosome transmission fidelity protein 18
DNA polymerase alpha-binding protein
RNA polymerase-associated protein CTR9
Pre-mRNA-splicing factor CWC22
Cystathionine beta-synthase
Serine/threonine-protein kinase DBF20
ATP-dependent RNA helicase DBP1
ATP-dependent RNA helicase DBP10
ATP-dependent RNA helicase DBP2
ATP-dependent RNA helicase DBP3
ATP-dependent RNA helicase DBP5

ATP-dependent RNA helicase DBP6
ATP-dependent RNA helicase DBP7
ATP-dependent RNA helicase DBP8
ATP-dependent RNA helicase DBP9
mRNA-decapping enzyme subunit 1
mRNA-decapping enzyme subunit 2
ATP-dependent RNA helicase DED1
Asparagine--tRNA ligase, cytoplasmic
Transcriptional regulatory protein DEP1
Broad-range acid phosphatase DET1
ATP-dependent RNA helicase DHH1
Probable ATP-dependent RNA helicase DHR2
Down-regulator of invasive growth 1
Dimethyladenosine transferase
U3 small nucleolar RNA-associated protein 12
Histone-lysine N-methyltransferase, H3 lysine-79 specific
Y element ATP-dependent helicase protein 1 copy 6;Y element ATP-dependent helicase
DNA polymerase epsilon subunit B
DNA polymerase epsilon subunit D
Dolichol-phosphate mannosyltransferase
ATP-dependent RNA helicase DRS1
Chromatin modification-related protein EAF1
Chromatin modification-related protein EAF3
Chromatin modification-related protein EAF5
rRNA-processing protein EBP2
Probable ATP-dependent RNA helicase DHR1
Proteasome component ECM29
3-methyl-2-oxobutanoate hydroxymethyltransferase
Putative ATP-dependent RNA helicase ECM32
Cell wall protein ECM33
Glutathione S-transferase omega-like 2
EH domain-containing and endocytosis protein 1
Elongation factor 1-beta
rRNA-processing protein EFG1
Protein-lysine N-methyltransferase EFM1
Elongation factor 2
Eisosome protein 1
Serine/threonine-protein kinase ELM1
Elongator complex protein 2
ER membrane protein complex subunit 1
ER membrane protein complex subunit 2
Ribosomal RNA small subunit methyltransferase NEP1
Enolase 2
Essential nuclear protein 1
Ribosome biogenesis protein ENP2
Late endosome and vacuole interface protein 11
Probable oxidoreductase ENV9
Enhancer of polycomb-like protein 1
Ribosome biogenesis protein ERB1
Acetyl-CoA acetyltransferase
Lanosterol 14-alpha demethylase
Delta(14)-sterol reductase
3-keto-steroid reductase
Delta(24(24(1)))-sterol reductase
Sterol 24-C-methyltransferase
ER-localized J domain-containing protein 5

Endoplasmic reticulum vesicle protein 25
ER-derived vesicles protein ERV29
ER-derived vesicles protein ERV46
Histone acetyltransferase ESA1
Silent chromatin protein ESC1
Pre-rRNA-processing protein ESF1
Enhancer of translation termination 1
Long-chain-fatty-acid--CoA ligase 3
Long-chain-fatty-acid--CoA ligase 4
Adenylate kinase isoenzyme 6 homolog FAP7
Fatty acid synthase subunit beta;3-hydroxyacyl-[acyl-carrier-protein] dehydratase;Enoyl-[acyl-carrier-protein] reductase [NADH];[Acyl-
Fatty acid synthase subunit alpha;Acyl carrier;3-oxoacyl-[acyl-carrier-protein] reductase;3-oxoacyl-[acyl-carrier-protein] synthase
Hsp70 nucleotide exchange factor FES1
Pre-rRNA-processing protein FHL1
1,3-beta-glucan synthase component FKS1
SUR7 family protein FMP45
GTP cyclohydrolase 1
FK506-binding nuclear protein
FK506-binding protein 4
Glycerol uptake/efflux facilitator protein
Fumarate reductase 1
Fatty acyl-CoA synthetase and RNA processing-associated kinase 1
Phenylalanine--tRNA ligase beta subunit
Phenylalanine--tRNA ligase alpha subunit
Eukaryotic translation initiation factor 5B
Mitogen-activated protein kinase FUS3
Mediator of RNA polymerase II transcription subunit 15
H/ACA ribonucleoprotein complex subunit 1
1,3-beta-glucanosyltransferase GAS1
1,3-beta-glucanosyltransferase GAS5
Transcriptional regulatory protein GAT1
Single-strand telomeric DNA-binding protein GBP2
Translation initiation factor eIF-2B subunit gamma
Eukaryotic translation initiation factor 2 subunit gamma
Translation initiation factor eIF-2B subunit delta
Translation initiation factor eIF-2B subunit epsilon
Translation initiation factor eIF-2B subunit beta
Translational activator GCN1
Serine/threonine-protein kinase GCN2
Protein GCN20
Translation initiation factor eIF-2B subunit alpha
Histone acetyltransferase GCN5
Glycolytic genes transcriptional activator GCR1
Glycolytic genes transcriptional activator GCR2
ADP-ribosylation factor GTPase-activating protein GCS1
ARF guanine-nucleotide exchange factor 1
ADP-ribosylation factor-binding protein GGA2
GTPase-interacting component 2
Serine/threonine-protein kinase GIN4
Zinc finger protein GIS2
Serine/threonine-protein phosphatase PP1-2
Glutamine synthetase
Nitrogen regulatory protein GLN3
Glutamine--tRNA ligase
ADP-ribosylation factor GTPase-activating protein GLO3
Glutathione reductase

Glyoxylate reductase 1
Guanine nucleotide-binding protein alpha-1 subunit
Guanine nucleotide-binding protein alpha-2 subunit
Guanine nucleotide-binding protein subunit beta 1
Guanine nucleotide-binding protein subunit beta 2
Heterotrimeric G protein gamma subunit GPG1
GPI transamidase component GPI16
Phosphoglycerate mutase 1
GPN-loop GTPase 3
NADPH-dependent methylglyoxal reductase GRE2
NADPH-dependent aldose reductase GRE3
GTP-binding nuclear protein GSP2/CNR2
Glycogen [starch] synthase isoform 2
GTP-binding protein GTR2
Glutathione S-transferase 1
GMP synthase [glutamine-hydrolyzing]
Guanylate kinase
Glutamate--tRNA ligase, cytoplasmic
GTPase-activating protein GYP1
GTPase-activating protein GYP5
GTPase-activating protein GYP6
GTPase-activating protein GYP7
GTPase-activating protein GYP8
Transcriptional activator HAA1
Serine/threonine-protein kinase HAL5
Halotolerance protein 9
Heme-responsive zinc finger transcription factor HAP1
Transcriptional activator HAP2
ATP-dependent RNA helicase HAS1
Histone acetyltransferase type B catalytic subunit
Putative nitroreductase HBN1
ATP-dependent RNA helicase HCA4
Hsp90 co-chaperone HCH1
Eukaryotic translation initiation factor 3 subunit J
Inner nuclear membrane protein HEH2
Heterogeneous nuclear rnp K-like protein 2
Transcriptional coactivator HFI1/ADA1
Histone H4
Histone H1
Histone H3
Protein HIR1
Histone transcription regulator 3
Histidinol-phosphate aminotransferase
Imidazole glycerol phosphate synthase hisHF;Glutamine amidotransferase;Cyclase
Protein HLJ1
3-hydroxy-3-methylglutaryl-coenzyme A reductase 1
Putative glycosyltransferase HOC1
Probable histone deacetylase HOS2
Histone deacetylase HOS3
High-osmolarity-induced transcription protein 1
Histone promoter control protein 2
Histidine protein methyltransferase 1
Protein HRB1
Serine/threonine-protein kinase HRK1
Nuclear polyadenylated RNA-binding protein 4
Casein kinase I homolog HRR25

ATP-dependent molecular chaperone HSC82
U2 snRNP component HSH155
Probable serine/threonine-protein kinase HSL1
Protein arginine N-methyltransferase HSL7
Heat shock protein 104
30 kDa heat shock protein
ATP-dependent molecular chaperone HSP82
NAD-dependent protein deacetylase HST1
NAD-dependent protein deacetylase HST2
Histone H2A.2;Histone H2A.1
Histone H2B.2
High temperature lethal protein 1
Histone H2A.Z
Protein HUA2
Eukaryotic translation initiation factor 5A-1
Ino eighty subunit 1
Ino eighty subunit 3
Ino eighty subunit 4
Ino eighty subunit 5
Very-long-chain 3-oxoacyl-CoA reductase
Elongator complex protein 1
Isoleucine--tRNA ligase, cytoplasmic
Vacuolar membrane-associated protein IML1
U3 small nucleolar ribonucleoprotein protein IMP3
U3 small nucleolar ribonucleoprotein protein IMP4
Putative DNA helicase INO80
ISWI one complex protein 2
ISWI one complex protein 3
ISWI one complex protein 4
Pre-rRNA-processing protein IPI3
Uncharacterized ATP-dependent helicase IRC20
Putative ATP-dependent helicase IRC3
Cohesin subunit SCC3
Inositol phosphosphingolipids phospholipase C
Vacuolar protein sorting-associated protein IST1
Increased sodium tolerance protein 2
ISWI chromatin-remodeling complex ATPase ISW1
ISWI chromatin-remodeling complex ATPase ISW2
Imitation switch two complex protein 1
Myo-inositol transporter 1
DnaJ-like chaperone JEM1
WD repeat-containing protein JIP5
Importin subunit beta-4
78 kDa glucose-regulated protein homolog
Kelch repeat-containing protein 1
Kelch repeat-containing protein 3
Serine/threonine protein kinase KIN1
Probable serine/threonine-protein kinase KKQ8
Glycolipid 2-alpha-mannosyltransferase
UPF0202 protein KRE33
Protein KRI1
KRR1 small subunit processome component
Lysine--tRNA ligase, cytoplasmic
Serine/threonine-protein kinase KSP1
Mitogen-activated protein kinase KSS1
Alpha-1,2 mannosyltransferase KTR1

Probable mannosyltransferase KTR3
Sphingosine N-acyltransferase LAC1
Serine palmitoyltransferase 1
Serine palmitoyltransferase 2
U3 small nucleolar ribonucleoprotein protein LCP5
Chromatin structure-remodeling complex protein RSC14
RNA polymerase-associated protein LEO1
3-isopropylmalate dehydratase
Regulatory protein LEU3
La protein homolog
Lysophosphatidic acid:oleoyl-CoA acyltransferase 1
60S ribosomal subunit assembly/export protein LOC1
Peroxisomal membrane protein LPX1
Phospholipid:diacylglycerol acyltransferase
Large subunit GTPase 1
Sm-like protein LSm1
Protein LSM12
U6 snRNA-associated Sm-like protein LSm2
U6 snRNA-associated Sm-like protein LSm4
U6 snRNA-associated Sm-like protein LSm5
U6 snRNA-associated Sm-like protein LSm6
Sphingolipid long chain base-responsive protein LSP1
Target of rapamycin complex subunit LST8
RING-finger protein MAG2
Protein MAK11
Protein MAK16
Ribosome biogenesis protein MAK21
ATP-dependent RNA helicase MAK5
Multiprotein-bridging factor 1
Transcription factor MBP1
Sister chromatid cohesion protein 1
GPI ethanolamine phosphate transferase 1
Pheromone receptor transcription factor
DNA replication licensing factor MCM2
DNA replication licensing factor MCM3
DNA replication licensing factor MCM4
Minichromosome maintenance protein 5
DNA replication licensing factor MCM6
DNA replication licensing factor MCM7
NADH-cytochrome b5 reductase 2;NADH-cytochrome b5 reductase p34 form;NADH-cytochrome b5 reductase p32 form
GTPase-activating protein GYP2
Negative regulator of sporulation MDS3
Serine/threonine-protein kinase MEC1
Mediator of RNA polymerase II transcription subunit 1
Mediator of RNA polymerase II transcription subunit 2
Methionine--tRNA ligase, cytoplasmic
Sulfite reductase [NADPH] flavoprotein component
DNA repair/transcription protein MET18/MMS19
5-methyltetrahydropteroyltriglutamate--homocysteine methyltransferase
mRNA export factor MEX67
Protein MGA2
MICOS complex subunit MIC10
MICOS complex subunit MIC12
MICOS subunit MIC26
MICOS complex subunit MIC27
MICOS complex subunit MIC60

Regulatory protein MIG1
DNA polymerase gamma
Myosin light chain 1
DNA mismatch repair protein MLH1
Probable alpha-1,6-mannosyltransferase MNN10
Probable alpha-1,6-mannosyltransferase MNN11
TATA-binding protein-associated factor MOT1
General negative regulator of transcription subunit 4
Transcriptional activator/repressor MOT3
Protein disulfide isomerase MPD2
ATP-dependent DNA helicase MPH1
U3 small nucleolar RNA-associated protein MPP10
Spindle pole body assembly component MPS3
Multiple RNA-binding domain-containing protein 1
Serine/threonine-protein kinase MRK1
RNA-binding protein MRN1
Ribosome assembly factor MRT4
Morphogenesis-related protein MSB1
Meiotic sister-chromatid recombination protein 3
DNA mismatch repair protein MSH2
Chromatin assembly factor 1 subunit p50
Protein MSN1
Zinc finger protein MSN2
Zinc finger protein MSN4
Maintenance of telomere capping protein 5
U1 small nuclear ribonucleoprotein A
Splicing factor MUD2
Crossover junction endonuclease MUS81
Peptide methionine sulfoxide reductase
Myosin-2
Myosin-3
Myosin-5
Nuclear polyadenylated RNA-binding protein NAB2
Nuclear polyadenylated RNA-binding protein 3
RNA-binding protein NAB6
NET1-associated nuclear protein 1
Nucleosome assembly protein
Probable 26S proteasome regulatory subunit p28
N-terminal acetyltransferase A complex subunit NAT1
N-terminal acetyltransferase A complex subunit NAT5
Nuclear control of ATPase protein 2
Negative cofactor 2 complex subunit beta
Non-classical export protein 2
Multisite-specific tRNA:(cytosine-C(5))-methyltransferase
NADPH--cytochrome P450 reductase
Nucleoporin NDC1
Kinetochore protein NDC80
Nucleolar protein NET1
Chromatin-remodeling complexes subunit NGG1
RNA exonuclease NGL1
Non-histone protein 10
H/ACA ribonucleoprotein complex subunit 2
Non-histone chromosomal protein 6A
Non-histone chromosomal protein 6B
Nucleoporin NIC96
Eukaryotic translation initiation factor 3 subunit C

60S ribosome subunit biogenesis protein NIP7
Nicotinamide/nicotinic acid mononucleotide adenylyltransferase 1
Nicotinamide/nicotinic acid mononucleotide adenylyltransferase 2
60S ribosomal export protein NMD3
20S-pre-rRNA D-site endonuclease NOB1
Nucleolar complex protein 2
Nucleolar complex-associated protein 3
Nucleolar complex protein 4
Nucleolar GTP-binding protein 1
Nucleolar GTP-binding protein 2
rRNA 2-O-methyltransferase fibrillarin
H/ACA ribonucleoprotein complex subunit 3
Nucleolar protein 12
Nucleolar protein 13
Nucleolar complex protein 14
Ribosome biogenesis protein 15
Nucleolar protein 16
25S rRNA (cytosine(2870)-C(5))-methyltransferase
Nucleolar protein 4
Ribosome biogenesis protein NOP53
Nucleolar protein 56
Nucleolar protein 58
Nucleolar protein 6
Pescadillo homolog
60S ribosome subunit biogenesis protein NOP8
Nucleolar protein 9
General negative regulator of transcription subunit 3
General negative regulator of transcription subunit 5
GPN-loop GTPase 1
Nucleolar protein 3
Chromatin structure-remodeling complex subunit RSC7
Nitrogen permease reactivator protein
Nitrogen permease regulator 2
Nitrogen permease regulator 3
Nicotinate phosphoribosyltransferase
Protein NRD1
Ribosome biogenesis protein NSA1
Ribosome biogenesis protein NSA2
Non-structural maintenance of chromosome element 3
Non-structural maintenance of chromosome element 4
RNA polymerase I termination factor
Nucleoporin NSP1
Nuclear localization sequence-binding protein
NuA3 HAT complex component NTO1
Protein NUD1
Kinetochore protein NUF2
Nuclear GTP-binding protein NUG1
Nuclear migration protein NUM1
Nucleoporin NUP116/NSP116
Nucleoporin NUP120
Nucleoporin NUP133
Nucleoporin NUP145;Nucleoporin NUP145N;Nucleoporin NUP145C
Nucleoporin NUP157
Nucleoporin NUP159
Nucleoporin NUP170
Nucleoporin NUP188

Nucleoporin NUP192
Nucleoporin NUP49/NSP49
Nucleoporin NUP57
Nucleoporin NUP82
Nucleoporin NUP84
Nucleoporin NUP85
Nuclear rim protein 1
Mediator of RNA polymerase II transcription subunit 5
Vacuolar v-SNARE NYV1
Oleate-activated transcription factor 1
Oleate activated transcription factor 3
Putative tyrosine-protein phosphatase OCA1
Initiation-specific alpha-1,6-mannosyltransferase
Central kinetochore subunit OKP1
Obg-like ATPase 1
Acyl-CoA desaturase 1
Phosphatidyl-N-methylethanolamine N-methyltransferase
Origin recognition complex subunit 1
Origin recognition complex subunit 2
Origin recognition complex subunit 3
Origin recognition complex subunit 4
Origin recognition complex subunit 5
Origin recognition complex subunit 6
Oxysterol-binding protein homolog 3
Fumarate reductase 2
Dolichyl-diphosphooligosaccharide--protein glycosyltransferase subunit 1
Dolichyl-diphosphooligosaccharide--protein glycosyltransferase subunit 3
Polyamine N-acetyltransferase 1
Polyadenylate-binding protein, cytoplasmic and nuclear
RNA polymerase II-associated protein 1
Protein PAL1
2-dehydropantoate 2-reductase
Poly(A) RNA polymerase protein 2
DNA topoisomerase 2-associated protein PAT1
PAB1-binding protein 1
PAB1-binding protein 2
Protein PBP4
Pyruvate decarboxylase isozyme 1
Protein disulfide-isomerase
Transcription factor PDR1
ATP-dependent permease PDR12
Pleiotropic ABC efflux transporter of multiple drugs
Sister chromatid cohesion protein PDS5
Vacuolar protein sorting/targeting protein VPS10
Vacuolar membrane protein PEP3
Protein PET54
ADP,ATP carrier protein 2
Peroxisomal membrane protein PMP27
Peroxisomal membrane protein import receptor PEX19
ATP-dependent 6-phosphofructokinase subunit alpha
ATP-dependent 6-phosphofructokinase subunit beta
Plasma membrane-associated coenzyme Q6 reductase PGA3
Glucose-6-phosphate isomerase
Phosphoglycerate kinase
Prohibitin-1
Prohibitin-2

Inorganic phosphate transport protein PHO88
Protein interacting with Hsp90 1
Sphingolipid long chain base-responsive protein PIL1
Peroxisome proliferation transcriptional regulator
CDP-diacylglycerol--inositol 3-phosphatidyltransferase
Protein kinase C-like 1
Serine/threonine-protein kinase PKH1
Serine/threonine-protein kinase PKH2
Serine/threonine-protein kinase PKH3
Plasma membrane ATPase 1
Pre-mRNA leakage protein 39
Dolichyl-phosphate-mannose--protein mannosyltransferase 1
Dolichyl-phosphate-mannose--protein mannosyltransferase 2
Dolichyl-phosphate-mannose--protein mannosyltransferase 4
Pre-rRNA-processing protein PNO1
Purine nucleoside phosphorylase
FACT complex subunit POB3
DNA polymerase epsilon catalytic subunit A
DNA polymerase delta catalytic subunit
Proliferating cell nuclear antigen
DNA polymerase delta small subunit
DNA polymerase V
Nucleoporin POM152
Pore membrane protein of 33 kDa
Ribonucleases P/MRP protein subunit POP1
Poly(A) ribonuclease POP2
Acyl-coenzyme A oxidase
Serine/threonine-protein phosphatase PP2A-1 catalytic subunit
Serine/threonine-protein phosphatase PP2A-2 catalytic subunit
Serine/threonine-protein phosphatase PP-Z1
Serine/threonine-protein phosphatase PP-Z2
Proteasome subunit beta type-4
Probable proteasome subunit alpha type-7
Proteasome subunit beta type-5
Proteasome subunit beta type-1
Proteasome subunit beta type-7
Proteasome subunit alpha type-6
Proteasome subunit alpha type-4
Proteasome subunit beta type-6
Proteasome subunit alpha type-2
Proteasome subunit alpha type-3
DNA primase small subunit
Actin-regulating kinase PRK1
Glutamate 5-kinase
Gamma-glutamyl phosphate reductase
Pyrroline-5-carboxylate reductase
Pre-mRNA-processing factor 39
U4/U6 small nuclear ribonucleoprotein PRP4
U1 small nuclear ribonucleoprotein component PRP42
Pre-mRNA-splicing factor ATP-dependent RNA helicase PRP43
Pre-mRNA-splicing factor 8
Serine/threonine-protein kinase PRR1
Eukaryotic translation initiation factor 3 subunit B
Mannose-1-phosphate guanyltransferase
RING finger protein PSH1
Serine/threonine-protein kinase PSK1

Protein PSP2
Phosphatase PSR1
Protein phosphatase 2C homolog 1
Protein phosphatase 2C homolog 3
Serine/threonine-protein kinase PTK2/STK2
Membrane protein PTM1
Nuclear and cytoplasmic polyadenylated RNA-binding protein PUB1
mRNA-binding protein PUF2
mRNA-binding protein PUF3
Pumilio homology domain family member 6
Proteasome subunit beta type-2
Proteasome subunit alpha type-5
Periodic tryptophan protein 2
Protein PXR1
Pyruvate carboxylase 1
Pyruvate carboxylase 2
DNA repair protein RAD16
Flap endonuclease 1
DNA repair protein RAD33
DNA repair protein RAD4
DNA repair protein RAD51
DNA repair and recombination protein RAD52
Trans-acting factor D
Protein farnesyltransferase/geranylgeranyltransferase type-1 subunit alpha
DNA-binding protein RAP1
Ras-like protein 2
Ribosome-interacting GTPase 1
Ribosome-interacting GTPase 2
Serine/threonine-protein kinase RCK2
25S rRNA (cytosine(2278)-C(5))-methyltransferase
Transcriptional regulatory protein RCO1
DNA-binding protein REB1
Partitioning protein REP1
Partitioning protein REP2
DNA-directed RNA polymerase III subunit RPC2
Coatomer subunit delta
Coatomer subunit zeta
RNA exonuclease 4
Replication factor A protein 1
Replication factor A protein 2
Replication factor A protein 3
Replication factor C subunit 1
Replication factor C subunit 2
Replication factor C subunit 3
Replication factor C subunit 4
Replication factor C subunit 5
Protein RFS1
Rho-type GTPase-activating protein 2
RHO GTPase-activating protein RGD1
Guanine nucleotide exchange factor subunit RGP1
Mediator of RNA polymerase II transcription subunit 14
GTP-binding protein RHO1
Telomere length regulator protein RIF1
Serine/threonine-protein kinase RIM11/MSD1
Serine/threonine-protein kinase RIO1
Serine/threonine-protein kinase RIO2

Ribosome biogenesis ATPase RIX7
Ribosomal lysine N-methyltransferase 2
Chromatin assembly factor 1 subunit p90
Translation initiation factor RLI1
Transcription factor RLM1
Ribosome biogenesis protein RLP24
Ribosome biogenesis protein RLP7
Protein arginine N-methyltransferase 2
Ran GTPase-activating protein 1
Ribonucleoside-diphosphate reductase large chain 1
Ribonucleoside-diphosphate reductase small chain 1
Ribonucleoside-diphosphate reductase small chain 2
Ribonuclease T2-like
ATP-dependent RNA helicase ROK1
RHO1 GDP-GTP exchange protein 2
DNA-directed RNA polymerase I subunit RPA12
DNA-directed RNA polymerase I subunit RPA135
DNA-directed RNA polymerase I subunit RPA14
DNA-directed RNA polymerase I subunit RPA190
DNA-directed RNA polymerase I subunit RPA34
DNA-directed RNA polymerase I subunit RPA43
DNA-directed RNA polymerase I subunit RPA49
DNA-directed RNA polymerases I, II, and III subunit RPABC5
DNA-directed RNA polymerase II subunit RPB11
DNA-directed RNA polymerase II subunit RPB2
DNA-directed RNA polymerase II subunit RPB3
DNA-directed RNA polymerase II subunit RPB4
DNA-directed RNA polymerases I, II, and III subunit RPABC1
DNA-directed RNA polymerases I, II, and III subunit RPABC3
DNA-directed RNA polymerases I, II, and III subunit RPABC4
DNA-directed RNA polymerase III subunit RPC9
DNA-directed RNA polymerases I and III subunit RPAC2
DNA-directed RNA polymerase III subunit RPC8
DNA-directed RNA polymerase III subunit RPC7
DNA-directed RNA polymerase III subunit RPC6
DNA-directed RNA polymerase III subunit RPC5
DNA-directed RNA polymerases I and III subunit RPAC1
DNA-directed RNA polymerase III subunit RPC4
DNA-directed RNA polymerase III subunit RPC3
Histone deacetylase RPD3
Ribosome production factor 1
Ribosome biogenesis protein RPF2
Eukaryotic translation initiation factor 3 subunit A
DNA damage-responsive transcriptional repressor RPH1
60S ribosomal protein L10
60S ribosomal protein L11-B;60S ribosomal protein L11-A
60S ribosomal protein L12-B;60S ribosomal protein L12-A
60S ribosomal protein L13-B;60S ribosomal protein L13-A
60S ribosomal protein L14-A
60S ribosomal protein L15-A
60S ribosomal protein L16-A
60S ribosomal protein L16-B
60S ribosomal protein L17-A
60S ribosomal protein L18-B;60S ribosomal protein L18-A
60S ribosomal protein L19-B;60S ribosomal protein L19-A
60S ribosomal protein L1-B;60S ribosomal protein L1-A

60S ribosomal protein L20-B;60S ribosomal protein L20-A
60S ribosomal protein L21-A
60S ribosomal protein L21-B
60S ribosomal protein L22-A
60S ribosomal protein L23-B;60S ribosomal protein L23-A
60S ribosomal protein L24-A
60S ribosomal protein L25
60S ribosomal protein L26-A
60S ribosomal protein L26-B
60S ribosomal protein L27-B;60S ribosomal protein L27-A
60S ribosomal protein L28
60S ribosomal protein L2-B;60S ribosomal protein L2-A
60S ribosomal protein L3
60S ribosomal protein L30
60S ribosomal protein L31-A
60S ribosomal protein L32
60S ribosomal protein L33-A
60S ribosomal protein L33-B
60S ribosomal protein L34-A;60S ribosomal protein L34-B
60S ribosomal protein L35-B;60S ribosomal protein L35-A
60S ribosomal protein L36-B;60S ribosomal protein L36-A
60S ribosomal protein L37-A
60S ribosomal protein L38
60S ribosomal protein L39
60S ribosomal protein L42-B;60S ribosomal protein L42-A
60S ribosomal protein L43-B;60S ribosomal protein L43-A
60S ribosomal protein L4-A
60S ribosomal protein L4-B
60S ribosomal protein L5
60S ribosomal protein L6-A
60S ribosomal protein L6-B
60S ribosomal protein L7-A
60S ribosomal protein L8-A
60S ribosomal protein L8-B
60S ribosomal protein L9-A
60S ribosomal protein L9-B
26S proteasome regulatory subunit RPN1
26S proteasome regulatory subunit RPN10
26S proteasome regulatory subunit RPN12
26S proteasome regulatory subunit RPN13
26S proteasome regulatory subunit RPN2
26S proteasome regulatory subunit RPN3
26S proteasome regulatory subunit RPN5
26S proteasome regulatory subunit RPN6
26S proteasome regulatory subunit RPN7
26S proteasome regulatory subunit RPN8
26S proteasome regulatory subunit RPN9
DNA-directed RNA polymerase II subunit RPB1
DNA-directed RNA polymerases I, II, and III subunit RPABC2
DNA-directed RNA polymerase III subunit RPC1
60S acidic ribosomal protein P0
Ribonuclease P/MRP protein subunit RPP1
60S acidic ribosomal protein P1-alpha
60S acidic ribosomal protein P2-alpha
60S acidic ribosomal protein P2-beta
40S ribosomal protein S0-B;40S ribosomal protein S0-A

40S ribosomal protein S10-A;40S ribosomal protein S10-B
40S ribosomal protein S11-B;40S ribosomal protein S11-A
40S ribosomal protein S12
40S ribosomal protein S13
40S ribosomal protein S14-A
40S ribosomal protein S15
40S ribosomal protein S16-B;40S ribosomal protein S16-A
40S ribosomal protein S17-B;40S ribosomal protein S17-A
40S ribosomal protein S18-B;40S ribosomal protein S18-A
40S ribosomal protein S19-B;40S ribosomal protein S19-A
40S ribosomal protein S1-A
40S ribosomal protein S1-B
40S ribosomal protein S2
40S ribosomal protein S20
40S ribosomal protein S21-B
40S ribosomal protein S22-B;40S ribosomal protein S22-A
40S ribosomal protein S23-B;40S ribosomal protein S23-A
40S ribosomal protein S24-B;40S ribosomal protein S24-A
40S ribosomal protein S25-A;40S ribosomal protein S25-B
40S ribosomal protein S26-B;40S ribosomal protein S26-A
40S ribosomal protein S27-B;40S ribosomal protein S27-A
40S ribosomal protein S28-A;40S ribosomal protein S28-B
40S ribosomal protein S29-A
40S ribosomal protein S29-B
40S ribosomal protein S3
40S ribosomal protein S30-B;40S ribosomal protein S30-A
Ubiquitin-40S ribosomal protein S31;Ubiquitin;40S ribosomal protein S31;Polyubiquitin;Ubiquitin
40S ribosomal protein S4-B;40S ribosomal protein S4-A
40S ribosomal protein S5
40S ribosomal protein S6-B;40S ribosomal protein S6-A
40S ribosomal protein S7-A
40S ribosomal protein S7-B
40S ribosomal protein S8-B;40S ribosomal protein S8-A
40S ribosomal protein S9-B
26S protease regulatory subunit 7 homolog
26S protease subunit RPT4
Ribosome quality control complex subunit 2
Ribosome assembly protein RRB1
ATP-dependent DNA helicase RRM3
RNA polymerase I-specific transcription initiation factor RRN10
RNA polymerase I-specific transcription initiation factor RRN5
RNA polymerase I-specific transcription initiation factor RRN9
Ribosomal RNA-processing protein 1
Ribosomal RNA-processing protein 12
Ribosomal RNA-processing protein 15
Ribosomal RNA-processing protein 17
ATP-dependent rRNA helicase RRP3
Exosome complex component RRP42
rRNA biogenesis protein RRP5
Exosome complex exonuclease RRP6
Ribosomal RNA-processing protein 7
25S rRNA (adenine(645)-N(1))-methyltransferase
Ribosomal RNA-processing protein 9
Regulator of ribosome biosynthesis
Regulator of rDNA transcription protein 14
Ribosome assembly protein 3

Chromatin structure-remodeling complex subunit RSC1
Chromatin structure-remodeling complex subunit RSC2
Chromatin structure-remodeling complex protein RSC3
Chromatin structure-remodeling complex protein RSC30
Chromatin structure-remodeling complex subunit RSC4
Chromatin structure-remodeling complex protein RSC58
Chromatin structure-remodeling complex protein RSC6
Chromatin structure-remodeling complex protein RSC8
Chromatin structure-remodeling complex subunit RSC9
Pre-mRNA-splicing factor RSE1
E3 ubiquitin-protein ligase RSP5
RNA polymerase-associated protein RTF1
Retrograde regulation protein 3
Probable serine/threonine-protein kinase RTK1
Reticulon-like protein 1
RNA polymerase II assembly factor RTP1
Serine/threonine-protein phosphatase 2A 56 kDa regulatory subunit delta isoform
Regulator of Ty1 transposition protein 102
Regulator of Ty1 transposition protein 103
Histone chaperone RTT106
Regulator of Ty1 transposition protein 107
RuvB-like protein 1
RuvB-like protein 2
Transcriptional regulatory protein RXT2
Phosphoinositide phosphatase SAC1
GTPase-activating protein SAC7
Small COPII coat GTPase SAR1
Something about silencing protein 10
Histone acetyltransferase SAS3
Something about silencing protein 4
Something about silencing protein 5
Co-chaperone protein SBA1
Single-stranded nucleic acid-binding protein
Sister chromatid cohesion protein 2
Protein SCD6
Intron-encoded endonuclease I-SceI
Serine/threonine-protein kinase SCH9
Proteasome subunit alpha type-1
Protein SCP160
Vesicle-associated membrane protein-associated protein SCS2
Glycerol-3-phosphate O-acyltransferase 1
Protein phosphatase 1 regulatory subunit SDS22
Protein transport protein SEC1
Protein transport protein SEC13
COPII coat assembly protein SEC16
Rab guanine nucleotide exchange factor SEC2
Coatomer subunit gamma
Protein transport protein SEC23
Coatomer subunit beta
Coatomer subunit beta
Coatomer subunit epsilon
Protein transport protein SEC31
Phosphomannomutase
Protein translocation protein SEC63
Putative guanine nucleotide-exchange factor SED4
Eisosome protein SEG1

Nucleoporin SEH1
Helicase SEN1
Phosphoserine aminotransferase
Serine--tRNA ligase, cytoplasmic
Histone-lysine N-methyltransferase, H3 lysine-36 specific
SED5-binding protein 3
Chromatin structure-remodeling complex subunit SFH1
Protein arginine N-methyltransferase SFM1
RNA-binding protein SGN1
ATP-dependent helicase SGS1
Serine/threonine-protein kinase BUR1
SWI5-dependent HO expression protein 3
Serine hydroxymethyltransferase, cytosolic
SIR4-interacting protein SIF2
Transcriptional regulatory protein SIN3
Protein SIP5
NAD-dependent histone deacetylase SIR2
Regulatory protein SIR3
Protein SIS1
Serine/threonine-protein phosphatase PP1-1
Superkiller protein 3
Transcription factor SKN7
CRE-binding bZIP protein SKO1
Suppressor of kinetochore protein 1
Serine/threonine-protein kinase SKY1
Actin cytoskeleton-regulatory complex protein SLA1
Probable 1-acyl-sn-glycerol-3-phosphate acyltransferase
DNA replication regulator SLD3
Small nuclear ribonucleoprotein-associated protein B
Structural maintenance of chromosomes protein 1
Structural maintenance of chromosomes protein 2
Structural maintenance of chromosomes protein 3
Structural maintenance of chromosomes protein 4
Structural maintenance of chromosomes protein 5
Structural maintenance of chromosomes protein 6
Small nuclear ribonucleoprotein Sm D1
Small nuclear ribonucleoprotein Sm D2
Small nuclear ribonucleoprotein Sm D3
Transcription factor SMP1
Small nuclear ribonucleoprotein F
Carbon catabolite-derepressing protein kinase
Transcription regulatory protein SNF12
Transcription regulatory protein SNF2
SWI/SNF chromatin-remodeling complex subunit SNF5
Transcription regulatory protein SNF6
Vacuolar-sorting protein SNF7
HSP70 co-chaperone SNL1
U1 small nuclear ribonucleoprotein 70 kDa homolog
Protein SNQ2
Probable DNA-binding protein SNT1
Pre-mRNA-splicing factor SNU114
13 kDa ribonucleoprotein-associated protein
56 kDa U1 small nuclear ribonucleoprotein component
U1 small nuclear ribonucleoprotein component SNU71
Protein SOF1
27S pre-rRNA (guanosine(2922)-2-O)-methyltransferase

ATP-dependent rRNA helicase SPB4
Spindle pole body component SPC105
Spindle pole body component 110
Kinetochore protein SPC25
Spindle pole component 29
Spindle pole body component SPC42
Spindle pole component SPC72
Spindle pole body component SPC97
Spindle pole body component SPC98
Manganese-transporting ATPase 1
Transcription factor SPN1
COMPASS component SPP1
Peroxisomal 2,4-dienoyl-CoA reductase SPS19
TATA-box-binding protein
FACT complex subunit SPT16
Transcription factor SPT20
Protein SPT23
Protein SPT3
Transcription elongation factor SPT4
Transcription elongation factor SPT5
Transcription elongation factor SPT6
Transcriptional activator SPT7
Transcription factor SPT8
Mediator of RNA polymerase II transcription subunit 20
Mediator of RNA polymerase II transcription subunit 17
Guanine nucleotide exchange factor SRM1
RNA-binding protein SRO9
Importin subunit alpha
Signal recognition particle receptor subunit alpha homolog
Signal recognition particle receptor subunit beta
Signal recognition particle subunit SRP21
Suppressor protein SRP40
Signal recognition particle subunit SRP54
Signal recognition particle subunit SRP68
Signal recognition particle subunit SRP72
ATP-dependent DNA helicase SRS2
Adenylyl cyclase-associated protein
Protein SSD1
Heat shock protein homolog SSE1
Ribosome biogenesis protein SSF1
RNA polymerase II subunit A C-terminal domain phosphatase SSU72
Ribosome-associated complex subunit SSZ1
Probable transcriptional regulatory protein STB4
Serine/threonine-protein kinase STE11
Protein STE12
Serine/threonine-protein kinase STE20
CAAX prenyl protease 1
Guanine nucleotide-binding protein subunit beta
Serine/threonine-protein kinase STE7
Nuclear protein STH1/NPS1
Heat shock protein STI1
Suppressor protein STM1
Nuclear cap-binding protein complex subunit 1
Suppressor protein STP22 of temperature-sensitive alpha-factor receptor and arginine permease
Zinc finger protein STP3
Zinc finger protein STP4

Dolichyl-diphosphooligosaccharide--protein glycosyltransferase subunit STT3
Transcription initiation factor IIB
RNA polymerase II transcriptional coactivator SUB1
ATP-dependent RNA helicase SUB2
Eukaryotic translation initiation factor 2 subunit alpha
Eukaryotic translation initiation factor 2 subunit beta
Suppressor of mar1-1 protein
Eukaryotic peptide chain release factor GTP-binding subunit
Protein SUR7
Survival factor 1
Styryl dye vacuolar localization protein 3
SWR1-complex protein 4
COMPASS component SWD2
SWI/SNF chromatin-remodeling complex subunit SWI1
SWI/SNF complex subunit SWI3
Regulatory protein SWI4
Transcriptional factor SWI5
Nucleolar protein SWM2
Dolichyl-diphosphooligosaccharide--protein glycosyltransferase subunit SWP1
SWI/SNF global transcription activator complex subunit SWP82
Helicase SWR1
SMY2 homolog 2
Suppressor of yeast profilin deletion
Transcription initiation factor TFIID subunit 1
Transcription initiation factor TFIID subunit 10
Transcription initiation factor TFIID subunit 11
Transcription initiation factor TFIID subunit 12
Transcription initiation factor TFIID subunit 14
Transcription initiation factor TFIID subunit 2
Transcription initiation factor TFIID subunit 3
Transcription initiation factor TFIID subunit 4
Transcription initiation factor TFIID subunit 5
Transcription initiation factor TFIID subunit 6
Transcription initiation factor TFIID subunit 7
Transcription initiation factor TFIID subunit 8
Transcription initiation factor TFIID subunit 9
tRNA acetyltransferase TAN1
Cell morphogenesis protein PAG1
Protein TBF1
Tricalbin-1
Tricalbin-2
Tricalbin-3
tRNA threonylcarbamoyladenosine dehydratase 2
Target of rapamycin complex 1 subunit TCO89
Probable transcription factor TDA9
Ty transcription activator TEC1
Elongation factor 1-alpha
Elongation factor 1-gamma 2
Telomere length regulation protein TEL2
Peroxisomal acyl-coenzyme A thioester hydrolase 1
Transcription factor tau 95 kDa subunit
Transcription factor tau 138 kDa subunit
Transcription factor tau 131 kDa subunit
Transcription factor tau 91 kDa subunit
Transcription factor tau 55 kDa subunit
Transcription factor tau 60 kDa subunit

Trimethylguanosine synthase
tRNA(His) guanylyltransferase
ATP-dependent RNA helicase eIF4A
Eukaryotic translation initiation factor 1A
Eukaryotic translation initiation factor 4B
Eukaryotic translation initiation factor 3 subunit I
Eukaryotic translation initiation factor 3 subunit G
Eukaryotic initiation factor 4F subunit p150
Eukaryotic translation initiation factor 5
Eukaryotic translation initiation factor 6
Transketolase 1
Transketolase 2
Translation machinery-associated protein 16
Translationally-controlled tumor protein homolog
Protein TMA23
Translation machinery-associated protein 46
Translation machinery-associated protein 64
Transcription initiation factor IIA subunit 2
Transcriptional regulatory protein TOD6
Topoisomerase 1-associated factor 2
E3 ubiquitin-protein ligase TOM1
Protein TOM71
DNA topoisomerase 1
DNA topoisomerase 2
Serine/threonine-protein kinase TOR1
Serine/threonine-protein kinase TOR2
Protein phosphatase PP2A regulatory subunit A
Triosephosphate isomerase
tRNA 2-phosphotransferase
Transcription-associated protein 1
tRNA (guanosine(18)-2-O)-methyltransferase
tRNA (cytidine(32)-2-O)-methyltransferase non-catalytic subunit TRM732
Tryptophan synthase
Thioredoxin reductase 1
Trafficking protein particle complex subunit 33
Peroxiredoxin TSA1
Target of rapamycin complex 2 subunit TSC11
Very-long-chain enoyl-CoA reductase
Ribosome biogenesis protein TSR1
Ribosome biogenesis protein TSR3
Thiosulfate sulfurtransferase TUM1
General transcriptional corepressor TUP1
Golgi apparatus membrane protein TVP18
Transposon Ty1-GR1 Gag polyprotein;Capsid protein
Transposon Ty1-JR2 Gag polyprotein;Capsid protein;Gag-p4
Transposon Ty1-LR3 Gag-Pol polyprotein;Capsid protein;Ty1 protease;Integrase;Reverse transcriptase/ribonuclease H
Transposon Ty1-ML2 Gag-Pol polyprotein;Capsid protein;Ty1 protease;Integrase;Reverse transcriptase/ribonuclease H
Transposon Ty1-PL Gag-Pol polyprotein;Capsid protein;Ty1 protease;Integrase;Reverse transcriptase/ribonuclease H;Transposon Ty1-[illegible]
Transposon Ty1-PR1 Gag-Pol polyprotein;Capsid protein;Ty1 protease;Integrase;Reverse transcriptase/ribonuclease H
Transposon Ty1-PR2 Gag-Pol polyprotein;Capsid protein;Ty1 protease;Integrase;Reverse transcriptase/ribonuclease H
Upstream activation factor subunit UAF30
Adenylyltransferase and sulfurtransferase UBA4;Adenylyltransferase UBA4;Sulfurtransferase UBA4
Ubiquitin carboxyl-terminal hydrolase 10
Ubiquitin carboxyl-terminal hydrolase 15
4-aminobutyrate aminotransferase
Transcriptional activator protein UGA3

ATP-dependent helicase ULS1
Transcriptional regulatory protein UME1
Transcriptional regulatory protein UME6
Protein URA2;Glutamine-dependent carbamoyl-phosphate synthase;Aspartate carbamoyltransferase
Orotate phosphoribosyltransferase 1
CTP synthase 1
CTP synthase 2
Nucleolar pre-ribosomal-associated protein 1
Nucleolar pre-ribosomal-associated protein 2
Uracil catabolism protein 2
Transcriptional regulator URE2
Nutrient and stress factor 1
U3 small nucleolar RNA-associated protein 10
U3 small nucleolar RNA-associated protein 11
U3 small nucleolar RNA-associated protein 13
U3 small nucleolar RNA-associated protein 14
U3 small nucleolar RNA-associated protein 15
U3 small nucleolar RNA-associated protein 18
U3 small nucleolar RNA-associated protein 20
U3 small nucleolar RNA-associated protein 21
U3 small nucleolar RNA-associated protein 22
rRNA-processing protein UTP23
U3 small nucleolar RNA-associated protein 25
Ribosome biogenesis protein UTP30
U3 small nucleolar RNA-associated protein 4
U3 small nucleolar RNA-associated protein 5
U3 small nucleolar RNA-associated protein 6
U3 small nucleolar RNA-associated protein 7
U3 small nucleolar RNA-associated protein 8
U3 small nucleolar RNA-associated protein 9
Vacuolar protein 8
Vacuolar morphogenesis protein 6
Vacuolar calcium ion transporter
Transcription factor VHR2
Vacuolar import and degradation protein 22
Vacuolar import and degradation protein 27
Vacuolar import and degradation protein 30
Inositol hexakisphosphate and diphosphoinositol-pentakisphosphate kinase
V-type proton ATPase catalytic subunit A;Endonuclease PI-SceI
V-type proton ATPase subunit H
V-type proton ATPase subunit B
V-type proton ATPase subunit E
V-type proton ATPase subunit C
V-type proton ATPase subunit d
V-type proton ATPase subunit a, vacuolar isoform
Vacuolar ATPase assembly integral membrane protein VPH2
Vacuolar protein sorting-associated protein 1
Vacuolar protein sorting-associated protein 13
Vacuolar protein sorting-associated protein 16
Vacuolar protein sorting-associated protein 17
Vacuolar protein sorting-associated protein 21
Vacuolar protein sorting-associated protein 30
Vacuolar protein sorting-associated protein 33
Vacuolar protein sorting-associated protein 4
Vacuolar protein sorting-associated protein 41
Vacuolar protein sorting-associated protein 64

Vacuolar protein sorting-associated protein 71
Vacuolar protein sorting-associated protein 72
Vacuolar protein sorting-associated protein 74
Vacuolar transporter chaperone 2
Vacuolar transporter chaperone 4
Protein VTS1
Dolichyl-diphosphooligosaccharide--protein glycosyltransferase subunit WBP1
Transcriptional modulator WTM1
Transcriptional repressor XBP1
DnaJ protein homolog XDJ1
Xanthine phosphoribosyltransferase 1
5-3 exoribonuclease 1
Protein AF-9 homolog
AP-1-like transcription factor YAP1
AP-1-like transcription factor YAP3
Carnitine O-acetyltransferase YAT2
Metal resistance protein YCF1
Condensin complex subunit 3
Casein kinase I homolog 1
Casein kinase I homolog 2
Casein kinase I homolog 3
Condensin complex subunit 1
Uncharacterized membrane protein YDL218W
Elongation factor 3A
Endoplasmic reticulum transmembrane protein 1
Endoplasmic reticulum transmembrane protein 3
Uncharacterized transcriptional regulatory protein YFL052W
Uncharacterized membrane protein YGL140C
Uncharacterized membrane protein YGR026W
Eukaryotic translation initiation factor 2A
Uncharacterized RNA-binding protein YGR250C
Flavohemoprotein
U1 small nuclear ribonucleoprotein C
ATP-dependent DNA helicase II subunit 1
ATP-dependent DNA helicase II subunit 2
Uncharacterized transcriptional regulatory protein YLR278C
Uncharacterized membrane protein YLR312C
Putative ATP-dependent RNA helicase YLR419W
DOCK-like protein YLR422W
PWWP domain-containing protein YLR455W
Uncharacterized membrane protein YML131W
Uncharacterized oxidoreductase YMR226C
Protein YNG1
Nucleoside diphosphate kinase
Uncharacterized vacuolar membrane protein YNL115C
UPF0674 endoplasmic reticulum membrane protein YNR021W
Serine/threonine-protein kinase YPK1
Serine/threonine-protein kinase YPK2/YKR2
Putative serine/threonine-protein kinase YPL150W
Cargo-transport protein YPP1
Putative reductase 1
Putative pyridoxal reductase
RNA annealing protein YRA1
Ran-specific GTPase-activating protein 1
Zinc finger transcription factor YRR1
Protein YSP1

Tat-binding homolog 7
Ribosome biogenesis protein YTM1
Calcium channel YVC1
Zinc finger protein ZPR1
Zinc/cadmium resistance protein
Zinc-regulated transporter 1
Probable quinone oxidoreductase
Zuotin
NADPH-dependent alpha-keto amide reductase
Protein-glutamate O-methyltransferase

Mitochondrial intermediate peptidase
Aspartate aminotransferase, mitochondrial
ARS-binding factor 2, mitochondrial
Acetyl-CoA hydrolase
Aconitate hydratase, mitochondrial
Homocitrate dehydratase, mitochondrial
Acetyl-coenzyme A synthetase 1
Alcohol dehydrogenase 3, mitochondrial
Alcohol dehydrogenase 4
GTP:AMP phosphotransferase, mitochondrial
ATPase expression protein 1, mitochondrial
ATPase expression protein 2, mitochondrial
ATPase expression protein 3
Protein AFG1
Mitochondrial respiratory chain complexes assembly protein AFG3
Alanine--glyoxylate aminotransferase 1
Altered inheritance of mitochondria protein 19, mitochondrial
Altered inheritance of mitochondria protein 24, mitochondrial
Altered inheritance rate of mitochondria protein 25
Altered inheritance of mitochondria protein 36, mitochondrial
Altered inheritance of mitochondria protein 41, mitochondrial
Probable electron transfer flavoprotein subunit alpha, mitochondrial
Altered inheritance of mitochondria protein 46, mitochondrial
Altered inheritance of mitochondria protein 9, mitochondrial
Alanine--tRNA ligase, mitochondrial
Potassium-activated aldehyde dehydrogenase, mitochondrial
Aldehyde dehydrogenase 5, mitochondrial
D-arabinono-1,4-lactone oxidase
Probable alanine aminotransferase, mitochondrial
Aminopeptidase 2, mitochondrial
Amino-acid acetyltransferase, mitochondrial
Protein ARG5,6, mitochondrial;N-acetyl-gamma-glutamyl-phosphate reductase;Acetylglutamate kinase
Arginine biosynthesis bifunctional protein ArgJ, mitochondrial;Glutamate N-acetyltransferase;Amino-acid acetyltransferase;Arginine
ATP synthase subunit alpha, mitochondrial
Mitochondrial ATPase complex subunit ATP10
Protein ATP11, mitochondrial
ATP synthase subunit epsilon, mitochondrial
ATP synthase subunit delta, mitochondrial
ATP synthase subunit f, mitochondrial
ATP synthase subunit beta, mitochondrial
ATP synthase subunit g, mitochondrial
Mitochondrial inner membrane protease ATP23
ATPase synthesis protein 25, mitochondrial
ATP synthase subunit gamma, mitochondrial
ATP synthase subunit 4, mitochondrial

ATP synthase subunit 5, mitochondrial
ATP synthase subunit d, mitochondrial
Branched-chain-amino-acid aminotransferase, mitochondrial
Biotin synthase, mitochondrial
Carnitine O-acetyltransferase, mitochondrial
5-demethoxyubiquinone hydroxylase, mitochondrial
Centromere-binding protein 1
Cytochrome B pre-mRNA-processing protein 1
Cytochrome B pre-mRNA-processing protein 2
Protein CBP3, mitochondrial
Assembly factor CBP4
Cytochrome B pre-mRNA-processing protein 6
Cytochrome b translational activator protein CBS1, mitochondrial
Cruciform cutting endonuclease 1, mitochondrial
Mitochondrial group I intron splicing factor CCM1
Cytochrome c peroxidase, mitochondrial
Probable electron transfer flavoprotein-ubiquinone oxidoreductase, mitochondrial
Citrate synthase, mitochondrial
Citrate synthase 3
Cytochrome c oxidase assembly factor 1
Hexaprenyl pyrophosphate synthase, mitochondrial
Coenzyme Q-binding protein COQ10, mitochondrial
Ubiquinone biosynthesis O-methyltransferase, mitochondrial
Ubiquinone biosynthesis protein COQ4, mitochondrial
2-methoxy-6-polyprenyl-1,4-benzoquinol methylase, mitochondrial
Ubiquinone biosynthesis monooxygenase COQ6, mitochondrial
Atypical kinase COQ8, mitochondrial
Ubiquinone biosynthesis protein COQ9, mitochondrial
Cytochrome b-c1 complex subunit 1, mitochondrial
Cytochrome c oxidase subunit 6B
Cytochrome c oxidase subunit 6A, mitochondrial
Cytochrome c oxidase assembly protein COX15
Cytochrome c oxidase subunit 2
Cytochrome c oxidase subunit 4, mitochondrial
Cytochrome c oxidase polypeptide 5A, mitochondrial
Cytochrome c oxidase subunit 6, mitochondrial
Peptidyl-prolyl cis-trans isomerase C, mitochondrial
Mitochondrial carnitine carrier
Peroxisomal catalase A
Tricarboxylate transport protein
Cytochrome b2, mitochondrial
Cytochrome c iso-1
Cytochrome c mitochondrial import factor CYC2
Mitochondrial presequence protease
Cytochrome c1, heme protein, mitochondrial
Mitochondrial dicarboxylate transporter
D-lactate dehydrogenase [cytochrome] 1, mitochondrial
D-lactate dehydrogenase [cytochrome] 2, mitochondrial
Exoribonuclease II, mitochondrial
Cys-Gly metallodipeptidase DUG1
Protein EFR3
3-hydroxyisobutyryl-CoA hydrolase, mitochondrial
Medium-chain fatty acid ethyl ester synthase/esterase 2
Enolase 1
Squalene synthase
Protein ERP1

Enoyl-[acyl-carrier protein] reductase [NADPH, B-specific], mitochondrial
Long-chain-fatty-acid--CoA ligase 1
Long-chain-fatty-acid--CoA ligase 2
5-formyltetrahydrofolate cyclo-ligase
Fructose-bisphosphate aldolase
Mitochondria fission 1 protein
ATP synthase assembly factor FMC1, mitochondrial
Uncharacterized mitochondrial membrane protein FMP10
Protein FMP27, mitochondrial
Uncharacterized mitochondrial hydrolase FMP41
Putative redox protein FMP46, mitochondrial
Protein FMP52, mitochondrial
Probable mitochondrial transport protein FSF1
Fumarate hydratase, mitochondrial
Aminomethyltransferase, mitochondrial
Glycine dehydrogenase (decarboxylating), mitochondrial
Glycine cleavage system H protein, mitochondrial
Glycogen debranching enzyme;4-alpha-glucanotransferase;Amylo-alpha-1,6-glucosidase
NAD-specific glutamate dehydrogenase
NADP-specific glutamate dehydrogenase 2
Genetic interactor of prohibitin 5, mitochondrial
Genetic interactor of prohibitin 7, mitochondrial
Mitochondrial GTP/GDP carrier protein 1
Glutamate synthase [NADH]
6-phosphogluconate dehydrogenase, decarboxylating 1
Glycerol-3-phosphate dehydrogenase [NAD(+)] 2, mitochondrial
Glycine--tRNA ligase 1, mitochondrial
Glutaredoxin-2, mitochondrial
Monothiol glutaredoxin-5, mitochondrial
Glycogen [starch] synthase isoform 1
Translation factor GUF1, mitochondrial
Glycerol kinase
Glycerol-3-phosphate dehydrogenase, mitochondrial
5-aminolevulinate synthase, mitochondrial
Protoporphyrinogen oxidase
Ferrochelatase, mitochondrial
Glutamyl-tRNA(Gln) amidotransferase subunit A, mitochondrial
Fatty aldehyde dehydrogenase HFD1
10 kDa heat shock protein, mitochondrial
Heat shock protein 60, mitochondrial
Heat shock protein 78, mitochondrial
Hydroxyacyl-thioester dehydratase type 2, mitochondrial
Histidine--tRNA ligase, mitochondrial
Mitochondrial 2-methylisocitrate lyase
Isocitrate dehydrogenase [NAD] subunit 1, mitochondrial
Isocitrate dehydrogenase [NAD] subunit 2, mitochondrial
Isocitrate dehydrogenase [NADP], mitochondrial
Translation initiation factor IF-2, mitochondrial
Threonine dehydratase, mitochondrial
Acetolactate synthase catalytic subunit, mitochondrial
Dihydroxy-acid dehydratase, mitochondrial
Ketol-acid reductoisomerase, mitochondrial
Acetolactate synthase small subunit, mitochondrial
54S ribosomal protein IMG1, mitochondrial
54S ribosomal protein IMG2, mitochondrial
Mitochondrial outer membrane protein IML2

Abhydrolase domain-containing protein IMO32
Inhibitory regulator protein IRA1
Inhibitory regulator protein IRA2
Protein ISD11
Protein JSN1
2-oxoglutarate dehydrogenase, mitochondrial
Dihydrolipoyllysine-residue succinyltransferase component of 2-oxoglutarate dehydrogenase complex, mitochondrial
Target of rapamycin complex 1 subunit KOG1
Cysteine proteinase 1, mitochondrial
Dihydrolipoyllysine-residue acetyltransferase component of pyruvate dehydrogenase complex, mitochondrial
2-isopropylmalate synthase
Lipoyl synthase, mitochondrial
Dihydrolipoyl dehydrogenase, mitochondrial
Succinyl-CoA ligase [ADP-forming] subunit alpha, mitochondrial
Succinyl-CoA ligase [ADP-forming] subunit beta, mitochondrial
Homoisocitrate dehydrogenase, mitochondrial
Homocitrate synthase, mitochondrial;Homocitrate synthase, cytosolic isozyme
Homoaconitase, mitochondrial
NAD-dependent malic enzyme, mitochondrial
Mitochondrial acidic protein MAM33
Mitochondrial-processing peptidase subunit beta
Mitochondrial-processing peptidase subunit alpha
Inner membrane mitoribosome receptor MBA1, mitochondrial
Malonyl CoA-acyl carrier protein transacylase, mitochondrial
Mitochondrial clpX-like chaperone MCX1
Malate dehydrogenase, mitochondrial
DnaJ homolog 1, mitochondrial
Mitochondrial distribution and morphology protein 38
Elongation factor G, mitochondrial
Probable proline--tRNA ligase, mitochondrial
Sulfate adenylyltransferase
Dynamin-like GTPase MGM1, mitochondrial;Dynamin-like GTPase MGM1 large isoform;Dynamin-like GTPase MGM1 small isoform
Mitochondrial genome maintenance protein MGM101
Mitochondrial inner membrane i-AAA protease supercomplex subunit MGR1
Mitochondrial homologous recombination protein 1
Mitochondrial phosphate carrier protein;Mitochondrial phosphate carrier protein, N-terminally processed
C-1-tetrahydrofolate synthase, mitochondrial;Methylenetetrahydrofolate dehydrogenase;Methenyltetrahydrofolate cyclohydrolase;[illegible]
Protein MMF1, mitochondrial
Protein MNE1
54S ribosomal protein L12, mitochondrial
tRNA dimethylallyltransferase, mitochondrial
Mitochondrial peculiar membrane protein 1
Protein MRH1
ATP-dependent RNA helicase MRH4, mitochondrial
rRNA methyltransferase 1, mitochondrial
37S ribosomal protein MRP1, mitochondrial
37S ribosomal protein MRP13, mitochondrial
37S ribosomal protein MRP17, mitochondrial
37S ribosomal protein MRP2, mitochondrial
54S ribosomal protein L41, mitochondrial
37S ribosomal protein MRP4, mitochondrial
54S ribosomal protein MRP49, mitochondrial
37S ribosomal protein MRP51, mitochondrial
54S ribosomal protein L2, mitochondrial
Uncharacterized protein MRP8
54S ribosomal protein L1, mitochondrial

54S ribosomal protein L10, mitochondrial
54S ribosomal protein L11, mitochondrial
54S ribosomal protein L13, mitochondrial
54S ribosomal protein L15, mitochondrial
54S ribosomal protein L16, mitochondrial
54S ribosomal protein L17, mitochondrial
54S ribosomal protein L19, mitochondrial
54S ribosomal protein L20, mitochondrial
54S ribosomal protein L22, mitochondrial
54S ribosomal protein L23, mitochondrial
54S ribosomal protein L24, mitochondrial
54S ribosomal protein L25, mitochondrial
54S ribosomal protein L28, mitochondrial
54S ribosomal protein L3, mitochondrial
54S ribosomal protein L31, mitochondrial
54S ribosomal protein L35, mitochondrial
54S ribosomal protein L36, mitochondrial
54S ribosomal protein L38, mitochondrial;54S ribosomal protein L34, mitochondrial
54S ribosomal protein L4, mitochondrial
54S ribosomal protein L40, mitochondrial
54S ribosomal protein L49, mitochondrial
54S ribosomal protein L51, mitochondrial
54S ribosomal protein L6, mitochondrial
54S ribosomal protein L7, mitochondrial
54S ribosomal protein L8, mitochondrial
54S ribosomal protein L9, mitochondrial
37S ribosomal protein S16, mitochondrial
37S ribosomal protein S17, mitochondrial
37S ribosomal protein S18, mitochondrial
37S ribosomal protein S28, mitochondrial
37S ribosomal protein S35, mitochondrial
37S ribosomal protein S5, mitochondrial
37S ribosomal protein S8, mitochondrial
37S ribosomal protein S9, mitochondrial
Mitochondrial RNA-splicing protein MRS1
Meiotic sister-chromatid recombination protein 6, mitochondrial
Aspartate--tRNA ligase, mitochondrial
DNA mismatch repair protein MSH1, mitochondrial
Protein MSP1
ATP-dependent RNA helicase MSS116, mitochondrial
Protein MSS18
Protein MSS2, mitochondrial
Protein MSS51, mitochondrial
Threonine--tRNA ligase, mitochondrial
Tryptophan--tRNA ligase, mitochondrial
Mitochondrial transcription factor 1
Mitochondrial transcription factor 2
Mitochondrial GTPase 1
Mitochondrial translation optimization protein 1
Myosin-4
Leucine--tRNA ligase, mitochondrial
37S ribosomal protein NAM9, mitochondrial
External NADH-ubiquinone oxidoreductase 1, mitochondrial
External NADH-ubiquinone oxidoreductase 2, mitochondrial
Rotenone-insensitive NADH-ubiquinone oxidoreductase, mitochondrial
Cysteine desulfurase, mitochondrial

NifU-like protein, mitochondrial
NGG1-interacting factor 3
Probable hydrolase NIT3
Mitochondrial 2-oxodicarboxylate carrier 1
Mitochondrial 2-oxodicarboxylate carrier 2
Mitochondrial outer membrane protein OM14
Mitochondrial outer membrane protein OM45
Mitochondrial metalloendopeptidase OMA1
Methyltransferase OMS1, mitochondrial
Mitochondrial ornithine transporter 1
Mitochondrial inner membrane protein OXA1
NADPH dehydrogenase 2
Phenylacrylic acid decarboxylase 1, mitochondrial
Presequence translocated-associated motor subunit PAM17, mitochondrial
Pyruvate dehydrogenase E1 component subunit alpha, mitochondrial
Pyruvate dehydrogenase E1 component subunit beta, mitochondrial
Pyruvate dehydrogenase complex protein X component, mitochondrial
Protein PET122, mitochondrial
37S ribosomal protein PET123, mitochondrial
Putative mitochondrial translation system component PET127
Protein PET130
Pentatricopeptide repeat-containing protein PET309, mitochondrial
Phosphatidylglycerol phospholipase C
Phosphatidylinositol 3-phosphate-binding protein 2
Mitochondrial phosphate carrier protein 2
Lon protease homolog, mitochondrial
[Pyruvate dehydrogenase (acetyl-transferring)] kinase 1, mitochondrial
[Pyruvate dehydrogenase (acetyl-transferring)] kinase 2, mitochondrial
DNA polymerase alpha catalytic subunit A
Mitochondrial outer membrane protein porin 1
NADH kinase POS5, mitochondrial
Inorganic pyrophosphatase, mitochondrial
Mitochondrial holo-[acyl-carrier-protein] synthase
Exopolyphosphatase
Saccharolysin
Mitochondrial peroxiredoxin PRX1
Phosphatidylserine decarboxylase proenzyme 1, mitochondrial;Phosphatidylserine decarboxylase 1 beta chain;Phosphatidylserine de
Protoplast secreted protein 2
Peptidyl-tRNA hydrolase
tRNA pseudouridine(27/28) synthase
21S rRNA pseudouridine(2819) synthase
tRNA pseudouridine(31) synthase
Delta-1-pyrroline-5-carboxylate dehydrogenase, mitochondrial
Pyruvate kinase 2
Uncharacterized mitochondrial protein RF1
Cytochrome b-c1 complex subunit 10
Cytochrome b-c1 complex subunit 2, mitochondrial
Cytochrome b-c1 complex subunit 6
Cytochrome b-c1 complex subunit 7
Cytochrome b-c1 complex subunit 8
UV excision repair protein RAD23
Respiratory supercomplex factor 2, mitochondrial
Thiosulfate sulfurtransferase RDL1, mitochondrial
Oligoribonuclease, mitochondrial
Single-stranded DNA-binding protein RIM1, mitochondrial
Cytochrome b-c1 complex subunit Rieske, mitochondrial

Protein RMD9, mitochondrial
54S ribosomal protein RML2, mitochondrial
Ribonuclease P protein component, mitochondrial
DNA-directed RNA polymerase, mitochondrial
Required for respiratory growth protein 1, mitochondrial
37S ribosomal protein S10, mitochondrial
37S ribosomal protein RSM18, mitochondrial
37S ribosomal protein S19, mitochondrial
37S ribosomal protein S22, mitochondrial
37S ribosomal protein S23, mitochondrial
37S ribosomal protein S24, mitochondrial
37S ribosomal protein S25, mitochondrial
37S ribosomal protein S26, mitochondrial
37S ribosomal protein RSM28, mitochondrial
37S ribosomal protein S7, mitochondrial
Retrograde regulation protein 2
Sorting assembly machinery 37 kDa subunit
Succinate dehydrogenase [ubiquinone] flavoprotein subunit, mitochondrial
Succinate dehydrogenase [ubiquinone] iron-sulfur subunit, mitochondrial
Succinate dehydrogenase [ubiquinone] cytochrome b small subunit, mitochondrial
Succinate/fumarate mitochondrial transporter
Serine hydroxymethyltransferase, mitochondrial
Mitochondrial tRNA-specific 2-thiouridylase 1
Sigma-like sequence protein 1, mitochondrial
Superoxide dismutase [Mn], mitochondrial
Protein SOV1, mitochondrial
Heat shock protein SSA2
Heat shock protein SSC1, mitochondrial
Heat shock protein SSQ1, mitochondrial
Protein STB1
Phosphatidylinositol 4-kinase STT4
ATP-dependent RNA helicase SUV3, mitochondrial
Mitochondrial chaperone TCM62
Glyceraldehyde-3-phosphate dehydrogenase 2
ATP synthase subunit e, mitochondrial
Mitochondrial import inner membrane translocase subunit TIM23
Mitochondrial import inner membrane translocase subunit TIM44
Mitochondrial import inner membrane translocase subunit TIM50
Mitochondrial import receptor subunit TOM20
Mitochondrial import receptor subunit TOM22
Mitochondrial import receptor subunit TOM40
Mitochondrial import receptor subunit TOM70
Trehalose-phosphatase
tRNA (guanine(26)-N(2))-dimethyltransferase, mitochondrial
Thioredoxin-3, mitochondrial
Elongation factor Tu, mitochondrial
Ribosomal protein VAR1, mitochondrial
Valine--tRNA ligase, mitochondrial
Putative mitochondrial carnitine O-acetyltransferase
Uncharacterized protein YBL095W, mitochondrial
Flavoprotein-like protein YCP4
Mitochondrial protein import protein MAS5
Putative carboxymethylenebutenolidase
Uncharacterized protein YER077C
Putative cysteine synthase
Putative pterin-4-alpha-carbinolamine dehydratase

Protein YIM1
Mitochondrial outer membrane protein YKR018C
Uncharacterized protein YKR023W
Uncharacterized protein YKR070W
LETM1 domain-containing protein YLH47, mitochondrial
Uncharacterized protein YLR290C, mitochondrial
Carrier protein YMC1, mitochondrial
Mitochondrial inner membrane i-AAA protease supercomplex subunit YME1
Mitochondrial escape protein 2
54S ribosomal protein YmL6, mitochondrial
37S ribosomal protein YMR-31, mitochondrial
Uncharacterized protein YNL208W
ABC1 family protein YPL109C, mitochondrial
Uncharacterized protein YPL168W
Uncharacterized mitochondrial carrier YPR011C
PX domain-containing protein YPR097W
GRAM domain-containing protein YSP2
Mitochondrial respiratory chain complexes assembly protein YTA12

E3 ubiquitin-protein ligase SNT2
Alanine/arginine aminopeptidase
Aspartate aminotransferase, cytoplasmic
Actin
Phosphoribosylaminoimidazole-succinocarboxamide synthase
Adenylosuccinate synthetase
Adenylosuccinate lyase
Phosphoribosylaminoimidazole carboxylase
C-1-tetrahydrofolate synthase, cytoplasmic;Methylenetetrahydrofolate dehydrogenase;Methenyltetrahydrofolate cyclohydrolase;For
Bifunctional purine biosynthetic protein ADE5,7;Phosphoribosylamine--glycine ligase;Phosphoribosylformylglycinamidine cyclo-liga
Alcohol dehydrogenase 1
Alcohol dehydrogenase 2
NADP-dependent alcohol dehydrogenase 6
Adenosine kinase
ATPase family gene 2 protein
Peroxiredoxin type-2
Putative COX1/OXI3 intron 1 protein
Putative COX1/OXI3 intron 2 protein
Protein AIM2
Altered inheritance of mitochondria protein 6
Actin-interacting protein 1
Protein AIR2
Aldehyde dehydrogenase [NAD(P)+] 1
Aldehyde dehydrogenase [NAD(P)+] 2
Magnesium-activated aldehyde dehydrogenase, cytosolic
Arrestin-related trafficking adapter 3
Mannan polymerase II complex ANP1 subunit
J domain-containing protein APJ1
AP-2 complex subunit beta
AP-3 complex subunit delta
AP-3 complex subunit beta
Adaptin medium chain homolog APM2
AP-3 complex subunit mu
AP-2 complex subunit mu
DNA-(apurinic or apyrimidinic site) lyase 1
Phosphatidate phosphatase APP1

AP-3 complex subunit sigma
D-arabinose dehydrogenase [NAD(P)+] heavy chain
ABC transporter ATP-binding protein ARB1
Actin-related protein 2/3 complex subunit 3
Actin-related protein 2/3 complex subunit 4
Actin-related protein 2/3 complex subunit 2
ADP-ribosylation factor 1
ADP-ribosylation factor 2
Argininosuccinate synthase
Argininosuccinate lyase
ADP-ribosylation factor-like protein 1
Transaminated amino acid decarboxylase
Chorismate synthase
Phospho-2-dehydro-3-deoxyheptonate aldolase, phenylalanine-inhibited
Phospho-2-dehydro-3-deoxyheptonate aldolase, tyrosine-inhibited
Aromatic/aminoadipate aminotransferase 1
Centractin
Actin-related protein 2
Actin-related protein 3
Actin-related protein 4
Actin-related protein 5
Actin-like protein ARP8
Asparagine synthetase [glutamine-hydrolyzing] 2
Autophagy-related protein 11
Autophagy-related protein 13
Autophagy-related protein 34
Ammonia transport outward protein 2
Late secretory pathway protein AVL9
Target of rapamycin complex 2 subunit AVO1
Target of rapamycin complex 2 subunit AVO2
Putative protease AXL1
Box C/D snoRNA protein 1
Protein BCH1
Protein BCP1
(R,R)-butanediol dehydrogenase
Bud emergence protein 1
Bud emergence protein 4
Glucan 1,3-beta-glucosidase
Intron-encoded RNA maturase bI4;Truncated, nonfunctional cytochrome b;RNA maturase bI4
Protein BIM1
Dethiobiotin synthetase
Probable target of rapamycin complex 2 subunit BIT2
Protein BMH2
Protein BNI1
Beige protein homolog 1
Bile pigment transporter 1
Mitotic check point protein BUB2
Inhibitory regulator protein BUD2/CLA2
Bud site selection protein 27
Bud site selection protein 3
Bud site selection protein 6
Cap-associated protein CAF20
Protein CAF40
Protein CAJ1
F-actin-capping protein subunit alpha
F-actin-capping protein subunit beta

Arginase
Ornithine aminotransferase
Regulatory protein CAT8
Cytochrome b termination protein 1
Protein CCC1
T-complex protein 1 subunit epsilon
T-complex protein 1 subunit eta
T-complex protein 1 subunit theta
Cell division control protein 10
Cell division control protein 11
Cell division control protein 15
Pyruvate kinase 1
Anaphase-promoting complex subunit CDC23
Cell division control protein 25
Cyclin-dependent kinase 1
Cell division control protein 31
Cell division control protein 53
Cell division control protein 7
Cytoplasmic export protein 1
Chitin synthase 2
Cytosolic iron-sulfur protein assembly protein 1
Probable electron transfer flavoprotein subunit beta
Choline kinase
Clustered mitochondria protein 1
**Calmodulin 1**
Calcium/calmodulin-dependent protein kinase I
Cofilin
Conserved oligomeric Golgi complex subunit 3
Conserved oligomeric Golgi complex subunit 5
Conserved oligomeric Golgi complex subunit 8
Protein CASP
Carbamoyl-phosphate synthase arginine-specific large chain
Peptidyl-prolyl cis-trans isomerase B
Exportin-1
Coronin-like protein
Importin alpha re-exporter
Phosphatidylinositol transfer protein CSR1
Catalase T
CUE domain-containing protein 3
Cytochrome b5
Adenylate cyclase
Cystathionine gamma-lyase
Dihydroxyacetone kinase 1
Cell cycle protein kinase DBF2
Lariat debranching enzyme
Delta(3,5)-Delta(2,4)-dienoyl-CoA isomerase
m7GpppX diphosphatase
Inactive diphosphatase DCS2
Mannan endo-1,6-alpha-mannosidase DCW1
DNA damage-inducible protein 1
Stress protein DDR48
DER1-like family member protein 1
2-deoxy-glucose resistant protein 2
Vacuolar protein-sorting-associated protein 46
DOA4-independent degradation protein 4
Down-regulator of invasive growth 2

Dynamin-related protein DNM1
Protein dopey
Peroxiredoxin DOT5
Sphingosine-1-phosphate lyase
Aspartate--tRNA ligase, cytoplasmic
Endo-1,3(4)-beta-glucanase 1
Urea amidolyase;Urea carboxylase;Allophanate hydrolase
Dynein heavy chain, cytoplasmic
Dynein light chain 1, cytoplasmic
Deoxyhypusine synthase
3,2-trans-enoyl-CoA isomerase
Putative metallocarboxypeptidase ECM14
UPF0045 protein ECM15
Protein ECM21
Protein ECM25
Protein ECM30
Protein ECM5
Enhancer of mRNA-decapping protein 3
Nascent polypeptide-associated complex subunit beta-1
Nascent polypeptide-associated complex subunit alpha
Elongation of fatty acids protein 2
Elongation of fatty acids protein 3
Elongator complex protein 3
Elongator complex protein 6
Putative glucokinase-2
Endosomal protein P24B
Protein EMP47
Epsin-2
Epsin-3
Epsin-5
ER-retained PMA1-suppressing protein 1
ER lumen protein-retaining receptor
Squalene monooxygenase
Mevalonate kinase
Hydroxymethylglutaryl-CoA synthase
C-8 sterol isomerase
Farnesyl pyrophosphate synthase
Sterol-4-alpha-carboxylate 3-dehydrogenase, decarboxylating
Ergosterol biosynthetic protein 28
Cytochrome P450 61
Phosphomevalonate kinase
Protein ERP4
FAD-linked sulfhydryl oxidase ERV2
Pre-rRNA-processing protein ESF2
Glucan 1,3-beta-glucosidase I/II
Exocyst complex component EXO84
Cyclin-dependent kinase inhibitor FAR1
Factor arrest protein 8
Fructose-1,6-bisphosphatase
rRNA-processing protein FCF1
rRNA-processing protein FCF2
Formate dehydrogenase 1
Peroxisomal hydratase-dehydrogenase-epimerase;2-enoyl-CoA hydratase;(3R)-3-hydroxyacyl-CoA dehydrogenase
Flippase kinase 1
FK506-binding protein 1
Putative Xaa-Pro aminopeptidase FRA1

Family of serine hydrolases 1
Protein FYV10
Glutamate decarboxylase
Galactokinase
Bifunctional protein GAL10;UDP-glucose 4-epimerase;Aldose 1-epimerase
Galactose transporter
Galactose-1-phosphate uridylyltransferase
SNF1 protein kinase subunit beta-3
Glycerol 2-dehydrogenase (NADP(+))
Glycerophosphodiester phosphodiesterase GDE1
NADP-specific glutamate dehydrogenase 1
Rab GDP-dissociation inhibitor
ATPase GET3
Golgi to ER traffic protein 4
Glutamine--fructose-6-phosphate aminotransferase [isomerizing]
Glucose-induced degradation protein 8
GLC7-interacting protein 2
Protein GIR2
Protein GIS4
1,4-alpha-glucan-branching enzyme
Glycogenin-1
Glucokinase-1
Lactoylglutathione lyase
Hydroxyacylglutathione hydrolase, cytoplasmic isozyme
Low specificity L-threonine aldolase
6-phosphogluconate dehydrogenase, decarboxylating 2
Golgi SNAP receptor complex member 1
Glycerol-3-phosphate dehydrogenase [NAD(+)] 1
Glycogen phosphorylase
Glycerol-3-phosphate O-acyltransferase 2
Monothiol glutaredoxin-3
Monothiol glutaredoxin-4
1,3-beta-glucan synthase component GSC2
Glucose-signaling factor 2
Glutathione transferase 3
Protein GVP36
Elongation factor 1 alpha-like protein
Protein HBT1
Elongation factor 3B
Delta-aminolevulinic acid dehydratase
HMG2-induced ER-remodeling protein 1
Protein HGH1
Protein HIR2
Histidine biosynthesis trifunctional protein;Phosphoribosyl-AMP cyclohydrolase;Phosphoribosyl-ATP pyrophosphohydrolase;Histidi
Protein HMF1
High mobility group protein 1
Heme-binding protein HMX1
Hit family protein 1
Mitogen-activated protein kinase HOG1
Aspartate-semialdehyde dehydrogenase
Aspartokinase
Homoserine dehydrogenase
Protein HOS4
Protein HSH49
Cell wall mannoprotein HSP150
Heat shock protein 26

Glutathione-independent glyoxalase HSP31
Probable glutathione-independent glyoxalase HSP32
Heat shock protein 42
Probable E3 ubiquitin-protein ligase HUL5
Hexokinase-1
Hexokinase-2
Probable glucose transporter HXT5
High-affinity hexose transporter HXT6
High-affinity hexose transporter HXT6
Protein ICE2
Isocitrate lyase
Isopentenyl-diphosphate Delta-isomerase
Isocitrate dehydrogenase [NADP] cytoplasmic
Isocitrate dehydrogenase [NADP]
Ino eighty subunit 2
Protein IFH1
Elongator complex protein 5
Inosine-5-monophosphate dehydrogenase 3
Sugar utilization regulatory protein IMP2
Inositol monophosphatase 2
Inositol-3-phosphate synthase
Polyphosphatidylinositol phosphatase INP52;SAC1-like phosphoinositide phosphatase;Phosphatidylinositol 4,5-bisphosphate 5-pho
Pre-rRNA-processing protein IPI1
Inorganic pyrophosphatase
Increased recombination centers protein 19
Increasing suppression factor 1
Protein IVY1
Intrastrand cross-link recognition protein
Carboxylic acid transporter protein homolog
Uncharacterized protein JIP4
Importin subunit beta-2
Importin beta-like protein KAP120
Importin beta-like protein KAP122
Importin subunit beta-1
Inositol hexakisphosphate kinase 1
Protein KES1
Dual specificity protein kinase KNS1
AP-1 accessory protein LAA1
Leukotriene A-4 hydrolase homolog
Sphingoid long chain base kinase 5
Heat shock protein 70 homolog LHS1
Exportin-T
LAS seventeen-binding protein 3
Protein LTV1
Protein LUC7
Saccharopine dehydrogenase [NAD(+), L-lysine-forming]
L-aminoadipate-semialdehyde dehydrogenase
Saccharopine dehydrogenase [NADP(+), L-glutamate-forming]
Methionine aminopeptidase 1
Methionine aminopeptidase 2
Metacaspase-1
Protein kinase MCK1
ABC1 family protein MCP2
Malate dehydrogenase, cytoplasmic
Malate dehydrogenase, peroxisomal
Midasin

Adenylyl-sulfate kinase
Protein MET17;O-acetylhomoserine sulfhydrylase;O-acetylserine sulfhydrylase
S-methyl-5-thioadenosine phosphorylase
MAP-homologous protein 1
Protein MKT1
Protein MLF3
Protein MLP2
Malate synthase 1, glyoxysomal
E3 ubiquitin-protein ligase linker protein MMS1
Mannan polymerase complexes subunit MNN9
Manganese resistance protein MNR2
DBF2 kinase activator protein MOB1
CBK1 kinase activator protein MOB2
Protein yippee-like MOH1
Protein MON2
Protein disulfide-isomerase MPD1
Methylthioribose-1-phosphate isomerase
Meiotic sister chromatid recombination protein 1
Putative aldehyde dehydrogenase-like protein YHR039C
DNA mismatch repair protein MSH6
Protein MSN5
Probable phosphatidylinositol 4-phosphate 5-kinase MSS4
Maintenance of telomere capping protein 1
Protein MUK1
Diphosphomevalonate decarboxylase
ATP-dependent helicase NAM7
Protein NAM8
NAP1-binding protein
NAP1-binding protein 2
[NU+] prion formation protein 1
Nonsense-mediated mRNA decay protein 2
Nonsense-mediated mRNA decay protein 5
Nitrogen network kinase 1
Transaldolase NQM1
Stress response protein NST1
Neutral trehalase
Oxysterol-binding protein homolog 2
Outer spore wall protein 5
OTU domain-containing protein 2
NADPH dehydrogenase 3
Protein PAM1
Protein PAR32
Protease B inhibitor 2
Protein PBN1
MAP kinase kinase PBS2
Probable tubulin--tyrosine ligase PBY1
Peroxisomal coenzyme A diphosphatase 1, peroxisomal
Phosphoenolpyruvate carboxykinase [ATP]
Peroxisomal-coenzyme A synthetase
Choline-phosphate cytidylyltransferase
Protein PDC2
Probable 2-methylcitrate dehydratase
Phosphatidylinositol transfer protein PDR16
Saccharopepsin
E3 ubiquitin-protein ligase PEP5
Protein PET10

Peroxisomal ATPase PEX1
Peroxisomal biogenesis factor 3
Profilin
Phosphoglucomutase 2
Regulatory protein PHO2
Phosphate system positive regulatory protein PHO81
Cyclin-dependent protein kinase PHO85
Inorganic phosphate transporter PHO86
Low-affinity phosphate transporter PHO91
GSY2-interacting protein PIG2
[PSI+] inducibility protein 3
Phosducin-like protein 1
Plasma membrane ATPase 2
Calcium-transporting ATPase 2
Negative regulator of sporulation PMD1
Mannose-6-phosphate isomerase
Calcium-transporting ATPase 1
Nicotinamidase
3-ketoacyl-CoA thiolase, peroxisomal
Endopolyphosphatase
Cerevisin
Carboxypeptidase Y
Pre-mRNA-processing factor 19
Ribose-phosphate pyrophosphokinase 1
Ribose-phosphate pyrophosphokinase 2
Ribose-phosphate pyrophosphokinase 3
Ribose-phosphate pyrophosphokinase 5
Phosphatidylserine decarboxylase proenzyme 2;Phosphatidylserine decarboxylase 2 beta chain;Phosphatidylserine decarboxylase 2 a
Importin subunit beta-3
Protein PSP1
Protein PTI1
Pumilio homology domain family member 4
Periodic tryptophan protein 1
Peroxisomal long-chain fatty acid import protein 2
Peroxisomal long-chain fatty acid import protein 1
Glutamine-dependent NAD(+) synthetase
UDP-N-acetylglucosamine pyrophosphorylase
RNA 3-terminal phosphate cyclase-like protein
Regulator of the glycerol channel 1
Respiratory growth induced protein 1
GTP-binding protein RHO3
GTP-binding protein RHO5
Ribosome assembly protein 1
GTP cyclohydrolase-2
6,7-dimethyl-8-ribityllumazine synthase
Meiotic activator RIM4
pH-response regulator protein palF/RIM8
Pre-rRNA-processing protein RIX1
Ribose-5-phosphate isomerase
Sporulation protein RMD1
Sporulation protein RMD8
[PIN+] prion protein RNQ1
Protein ROD1
Ubiquitin carboxyl-terminal hydrolase RPN11
26S protease regulatory subunit 4 homolog
26S protease regulatory subunit 6B homolog

26S protease regulatory subunit 6A
26S protease regulatory subunit 8 homolog
Exosome complex component RRP4
Exosome complex component RRP45
Respiration factor 1
Restriction of telomere capping protein 1
Reticulon-like protein 2
Reduced viability upon starvation protein 161
Reduced viability upon starvation protein 167
Fimbrin
Adenosylhomocysteinase
S-adenosylmethionine synthase 1
S-adenosylmethionine synthase 2
Protein SAP1
SIT4-associating protein SAP155
Protein transport protein SBH2
DnaJ-related protein SCJ1
Probable family 17 glucosidase SCW10
Probable family 17 glucosidase SCW4
Protein SDA1
Protein SDS23
Protein SDS24
SEH-associated protein 4
Exocyst complex component SEC10
Signal peptidase complex catalytic subunit SEC11
SEC14 cytosolic factor
Exocyst complex component SEC15
Alpha-soluble NSF attachment protein
Vesicular-fusion protein SEC18
Protein transport protein SEC24
Exocyst complex component SEC3
Ras-related protein SEC4
Exocyst complex component SEC5
Exocyst complex component SEC6
Protein transport protein SEC61
Translocation protein SEC62
Protein transport protein SEC7
Exocyst complex component SEC8
D-3-phosphoglycerate dehydrogenase 1
D-3-phosphoglycerate dehydrogenase 2
SET domain-containing protein 3
Protein SEY1
Protein SFI1
Suppressor of glycerol defect protein 1
SAGA-associated factor 29
SAGA-associated factor 73
Small glutamine-rich tetratricopeptide repeat-containing protein 2
Outer spore wall assembly protein SHE10
SWI5-dependent HO expression protein 2
SWI5-dependent HO expression protein 4
Seventh homolog of septin 1
SNF1 protein kinase subunit beta-1
Protein SIP3
Regulatory protein SIR4
Protein SKG3
Antiviral helicase SKI2

Antiviral protein SKI8
Protein SKT5
Protein SLA2
Protein SLF1
Antiviral helicase SLH1
Phosphatidylinositol 4,5-bisphosphate-binding protein SLM1
Phosphatidylinositol 4,5-bisphosphate-binding protein SLM2
Mitogen-activated protein kinase SLT2/MPK1
Protein SLY1
Cell wall assembly regulator SMI1
Protein SMY2
5-AMP-activated protein kinase subunit gamma
Sorting nexin-3
Sorting nexin-4
Sorting nexin-41
Probable pyridoxal 5-phosphate synthase subunit SNZ2;Probable pyridoxal 5-phosphate synthase subunit SNZ3
Protein SOK1
6-phosphogluconolactonase-like protein 2
Spermidine synthase
Sporulation-specific protein 73
Protein SPP41
Protein SPT2
Ribosome assembly protein SQT1
Lethal(2) giant larvae protein homolog SRO7
Heat shock protein SSA1
Heat shock protein SSA3
Heat shock protein SSA4
Heat shock protein SSB1
Heat shock protein SSB2
Heat shock protein homolog SSE2
Sec sixty-one protein homolog
MAP kinase kinase kinase SSK2
Protein SSO2
Protein STB3
Protein STB5
Protein STU2
Eukaryotic peptide chain release factor subunit 1
SWR1-complex protein 3
Oxysterol-binding protein homolog 1
Regulatory protein SWI6
Importin beta SMX1
Synchronized import protein 1
Cell division cycle protein CDT1
Transaldolase
Type 2A phosphatase-associated protein 42
T-complex protein 1 subunit alpha
Glyceraldehyde-3-phosphate dehydrogenase 1
Glyceraldehyde-3-phosphate dehydrogenase 3
Protein TEX1
Carboxypeptidase Y inhibitor
Sterol esterase TGL1
Lipase 4
THO complex subunit 2
Homoserine kinase
Threonine synthase
Threonine--tRNA ligase, cytoplasmic

Protein TMA108
Protein TOS1
Tropomyosin-1
Polyamine transporter 3
Alpha,alpha-trehalose-phosphate synthase [UDP-forming] 56 kDa subunit
Trehalose synthase complex regulatory subunit TPS3
Protein TRI1
Anthranilate synthase component 1
Multifunctional tryptophan biosynthesis protein;Anthranilate synthase component 2;Indole-3-glycerol phosphate synthase
Trafficking protein particle complex II-specific subunit 120
Trafficking protein particle complex subunit 31
Thioredoxin-1
Thioredoxin-2
Trehalose synthase complex regulatory subunit TSL1
Tubulin alpha-1 chain
Tubulin beta chain
Tubulin alpha-3 chain
Tubulin gamma chain
Twinfilin-1
Prephenate dehydrogenase [NADP(+)]
Tyrosine--tRNA ligase, cytoplasmic
S-adenosyl-L-methionine-dependent tRNA 4-demethylwyosine synthase
Ubiquitin-activating enzyme E1 1
Ubiquitin-conjugating enzyme E2 1
Ubiquitin-conjugating enzyme E2-16 kDa
Ubiquitin carboxyl-terminal hydrolase 1
Ubiquitin carboxyl-terminal hydrolase 11
Ubiquitin carboxyl-terminal hydrolase 13
Ubiquitin carboxyl-terminal hydrolase 2
Ubiquitin carboxyl-terminal hydrolase 3
Ubiquitin carboxyl-terminal hydrolase 6
Ubiquitin carboxyl-terminal hydrolase 7
Ubiquitin carboxyl-terminal hydrolase 8
E3 ubiquitin-protein ligase UBR1
UBX domain-containing protein 2
UBX domain-containing protein 3
UBX domain-containing protein 5
UBX domain-containing protein 7
E4 ubiquitin-protein ligase UFD2
Ubiquitin fusion degradation protein 4
Succinate-semialdehyde dehydrogenase [NADP(+)]
UTP--glucose-1-phosphate uridylyltransferase
Ubiquitin-like-specific protease 1
Nonsense-mediated mRNA decay protein 3
Dihydroorotate dehydrogenase (fumarate)
Orotidine 5-phosphate decarboxylase
U1 SNP1-associating protein 1
Vacuole morphology and inheritance protein 14
Mannan polymerase I complex VAN1 subunit
V-type proton ATPase subunit F
V-type proton ATPase subunit D
Growth regulation protein
Protein WHI4
Tryptophan--tRNA ligase, cytoplasmic
Uncharacterized protein YBL059W
YAP1-binding protein 1

YAP1-binding protein 2
Uncharacterized glycosyl hydrolase YBR056W
Uncharacterized protein YBR225W
Uncharacterized transporter YBR287W
Uncharacterized protein YCR016W
UPF0743 protein YCR087C-A
Uncharacterized protein YDL027C
Uncharacterized ABC transporter ATP-binding protein YDR061W
UPF0661 TPR repeat-containing protein YDR161W
Uncharacterized protein YDR239C
FAS1 domain-containing protein YDR262W
Uncharacterized protein YDR476C
Uncharacterized protein YDR514C
Putative 2-hydroxyacyl-CoA lyase
Uncharacterized protein YEL137C
UPF0160 protein YER156C
Uncharacterized protein YFL042C
Uncharacterized protein YGL082W
Uncharacterized protein YGR122W
Uncharacterized protein YGR130C
Uncharacterized GTP-binding protein YGR210C
Uncharacterized protein YGR237C
Citrate/oxoglutarate carrier protein
Putative proline--tRNA ligase YHR020W
Uncharacterized protein YHR033W
Uncharacterized protein YHR080C
Uncharacterized protein YHR097C
Uncharacterized protein YHR127W
Uncharacterized protein YHR182W
Putative zinc metalloproteinase YIL108W
Uncharacterized protein YIL161W
Prenylated Rab acceptor 1
Protein YIP5
Uncharacterized protein YJL147C
Cell wall protein YJL171C
MEMO1 family protein YJR008W
Uncharacterized protein YJR111C
Uncharacterized protein YKL023W
Putative uncharacterized hydrolase YKL033W-A
Uncharacterized protein YKL075C
Uncharacterized protein YKL077W
Probable intramembrane protease YKL100C
Synaptobrevin homolog YKT6
Obg-like ATPase homolog
Uncharacterized protein YLL007C
KH domain-containing protein YLL032C
Uncharacterized protein YLR072W
Uncharacterized protein YLR179C
Putative 6-phosphofructo-2-kinase/fructose-2,6-bisphosphatase YLR345W;6-phosphofructo-2-kinase;Fructose-2,6-bisphosphatase
Probable ADP-ribose 1-phosphate phosphatase YML087W
Uncharacterized protein YML119W
Uncharacterized protein YMR160W
Uncharacterized protein YMR196W
Uncharacterized protein YMR265C
Uncharacterized protein YMR315W
Uncharacterized phosphatase YNL010W

Uncharacterized protein YNL050C
Uncharacterized protein YNL134C
Uncharacterized protein YNR034W-A
Uncharacterized protein YNR040W
Uncharacterized WD repeat-containing protein YOL087C
Protein YOP1
Uncharacterized protein YOR093C
Putative uncharacterized hydrolase YOR131C
Protein OS-9 homolog
Phosphorelay intermediate protein YPD1
AGC kinase YPK3
Uncharacterized protein YPL034W
Uncharacterized protein YPL067C
Putative aryl-alcohol dehydrogenase YPL088W
Uncharacterized protein YPL191C
Protein PBDC1 homolog
UPF0662 protein YPL260W
Uncharacterized protein YPR084W
Dilute domain-containing protein YPR089W
Uncharacterized PH domain-containing protein YPR091C
Uncharacterized protein YPR148C
GTP-binding protein YPT1
GTP-binding protein YPT32/YPT11;GTP-binding protein YPT31/YPT8
PX domain-containing protein YPT35
GTP-binding protein YPT52
GTP-binding protein YPT7
RNA annealing protein YRA2
ADP-ribose pyrophosphatase
Protein YSC84
Probable 26S protease subunit YTA6
Protein ZPS1
Glucose-6-phosphate 1-dehydrogenase
Arginine--tRNA ligase, cytoplasmic
Cysteine--tRNA ligase
Glucose-6-phosphate 1-epimerase
NAD(P)H-hydrate epimerase
S-formylglutathione hydrolase

Supplementary Table S4. Fold-change expression values for DEG in both DM and PS yeast are indicated.

Gene Standard Name	PS	DM	Gene Systematic Name	Annotation
GRE1	2.4	4.9	YPL223C	Genes de Respuesta a Estres 1 (spanish for stress responsive genes)
AGP1		4.6	YCL025C	Glutamine permease
SIP18	1.6	4.5	YMR175W	Salt Induced Protein 18; paralog to GRE1
GEX1	2.8	4.2	YCL073C	Glutathione EXchanger
SIT1		3.5	YEL065W	Siderophore Iron Transport 1
RPL18B		3.4	YNL301C	Ribosomal 60S subunit protein L18B
FIT2		3.3	YOR382W	Facilitator of Iron Transport
ENO2	1.7	3.3	YHR174W	Enolase 2
STL1		3.2	YDR536W	Glycerol proton symporter; plasma membrane
THI4		3.2	YGR144W	Thiazole synthase; thiamine biosynthesis
DIT2		3.2	YDR402C	DITyrosine 2
CUP1-2	1.9	3.0	YHR055C	Metallothionein CUP1-2
FET3	-1.8	3.0	YMR058W	FErrous Transport 3; ferroxidase
CUP1-1	1.9	3.0	YHR053C	Metallothionein CUP1-1
FOX2	-2.5	-3.0	YKR009C	Hydroxyacyl-CoA dehydrogenase/enoyl-CoA hydratase (bifunctional)
FDH2	-1.8	-3.1	YPL275W	Formate DeHydrogenase 2; pseudogene
CYC7	-1.6	-3.2	YEL039C	CYtochrome C 7
ADH2	-2.9	-3.2	YMR303C	Alcohol DeHydrogenase 2
YHI9	-1.9	-3.2	YHR029C	Protein of unknown function
AAD4		-3.2	YDL243C	Aryl-Alcohol Dehydrogenase 4
MIG2	-2.2	-3.3	YGL209W	Multicopy Inhibitor of GAL gene expression 2
OPT2		-3.3	YPR194C	OligoPeptide Transporter 2
SCW11	1.7	-3.3	YGL028C	Soluble Cell Wall protein 11; glucan endo-1,3-beta-D-glucosidase
HSP30		-3.3	YCR021C	Heat Shock Protein 30
FDH1	-4.4	-3.5	YOR388C	Formate DeHydrogenase 1
PXA1		-3.6	YPL147W	PeroXisomal ABC-transporter 1
HXT3	-5.7	-3.6	YDR345C	HeXose Transporter 3
BDH1	-2.0	-3.6	YAL060W	Butanediol DeHydrogenase 1
GAL2		-3.7	YLR081W	GALactose metabolism 2
YCT1		-3.8	YLL055W	Yeast Cysteine Transporter 1
ASP3-2	-1.7	-3.8	YLR157C	ASParaginase 3-2
ASP3-4	-1.7	-3.9	YLR160C	ASParaginase 3-4
ASP3-1	-1.7	-3.9	YLR155C	ASParaginase 3-1
ASP3-3	-1.7	-3.9	YLR158C	ASParaginase 3-3
DAL4	-5.0	-4.5	YIR028W	Degradation of Allantoin 4; allantoin permease
BDH2		-5.3	YAL061W	Medium-chain alcohol dehydrogenase with similarity to BDH1
DAL5	-3.3	-8.5	YJR152W	Degradation of Allantoin 5; allantoate permease
OPT1	-1.7	-9.3	YJL212C	OligoPeptide Transporter 1

Supplementary Table S5. qRT-PCR verification of RNA-seq expression values for five selected DE genes in PS- and DM-expressing yeast. qRT-PCR expression values are determined by comparing expression levels to empty vector (EV) yeast line, which is set to "1" for each gene tested. NDE indicates not differentially expressed (≤ 1.5-fold) in RNA-seq and qRT data for that gene. PCR primers used for each gene are indicated in Supplementary Table S1. Student's t-test analysis shows *p ≤ o.05, **p ≤ 0.01.

SGD ID	Symbol	Annotation	Fold-Change Expression			
			RNA-seq		qRT-PCR	
			PS	DM	PS	DM
Intracellular P storage and remobilization						
S000005940	VTC3	polyphosphate synthesis, regulatory subunit of the vacuolar transporter chaperone (VTC) complex	2.1	NDE	1.99**	NDE
Intracellular Recycling of P						
S000006031	GDE1	Phosphodiesterase; product serves as a phosphate source	1.5	NDE	3.3*	NDE
Pi Transporters						
S000000695	GIT1	Glycerolphosphoinositol 1; PM permease; substrates as sources of inositol and phosphate; Pi uptake	5.8	2.2	4.38	4.61
Nitrogen transport and metabolism						
S000003913	DAL5	Degradation of Allantoin 4; allantoin permease	-3.3	-8.5	-2.04*	-3.44*
S000004145	ASP3-1	ASParaginase 3-1	-1.7	-3.9	-1.96*	-2.3**
S000005909	FIT2	Facilitator of iron transport	NDE	3.3	NDE	2.18
S000000530	AGP1	Glutamine permease	NDE	4.6	NDE	1.83*

Supplementary Table S6. Differential expression of genes that function in phosphate homeostasis in yeast PS and DM lines. Genes with PS-specific binding sites in their promoters are indicated and gene models for binding sites and their potential target genes are shown in **Figure x.** Grey-shaded genes are Pho4p-regulated in WT yeast (Zhou and Shea 2011, https://doi.org/10.1016/j.molcel.2011.05.025 ; SGD annotation). InsPPs bind SPX domains (*) of proteins in the INPHORS (intracellular phosphate reception and signaling) pathway that regulate Pi homeostasis, in coordination with transcriptional regulation of *PHO* regulon genes; Austin and Mayer 2020).

Standard Name	SPX-Domain Protein*	Function	Expression FC		PS-Specific Binding SIte
			PS	DM	
**Extracellular Foraging for P**					
PHO3		Acid phosphatase PHO3, constitutive, secreted	2.9		
PHO5		Repressible acid phosphatase, secreted			
PHO11		Repressible acid phosphatase, secreted	1.8		X
PHO12		Repressible acid phosphatase, secreted	2.5	1.5	X
**Pi Transporters**					
ERD1		regulates ER luminal protein retention; Pi export from Golgi to cytosol	1.6		X
GIT1		Glycerolphosphoinositol 1; PM permease; substrates are sources of inositol and phosphate; Pi uptake	5.8	2.2	
PHO84		High affinity Pi transporter (expressed in *pho84* mutant but non-functional)	3.4		
PHO87	X	Low-affinity inorganic phosphate (Pi) transporter; ; not regulated in response to Pi limitation			
PHO89		Plasma membrane Na+/Pi cotransporter; high-affinity Pi uptake	1.8		
PHO90	X	Low-affinity inorganic phosphate (Pi) transporter; ; not transcriptionally regulated in response to Pi limitation	-2.4	-2.1	
PHO91	X	Low-affinity vacuolar inorganic phosphate (Pi) exporter; not transcriptionally regulated in response to Pi limitation			
SYG1	X	Suppressor of Yeast GPA1; putative Pi exporter; PM		-1.5	
**Intracellular Recycling of P**					
GDE1	X	Phosphodiesterase; product serves as a phosphate source	1.5		X
GPP2		Glycerol-3-Phosphate phosphatase			
PHM8		Lysophosphatidic acid phosphatase, nucleotidase; response to phosphate starvation		1.5	
**Intracellular Storage, Remobilization of P**					
PHO8		Repressible vacuolar alkaline phosphatase; controls polyphosphate content; regulated by levels of Pi			
PPN1		Dual endo- and exopolyphosphatase; not regulated by Pi levels			
PPN2		Repressible vacuolar alkaline phosphatase; polyphosphate metabolism; Zn2+-dependent endopolyphosphatase	1.6		X
VTC1		polyphosphate synthesis, regulatory subunit of the vacuolar transporter chaperone (VTC) complex			
VTC2	X	polyphosphate synthesis, regulatory subunit of the vacuolar transporter chaperone (VTC) complex			
VTC3	X	polyphosphate synthesis, regulatory subunit of the vacuolar transporter chaperone (VTC) complex	2.1		
VTC4	X	polyphosphate synthesis, subunit of the vacuolar transporter chaperone (VTC) complex	1.5	-1.5	
VTC5	X	polyphosphate synthesis, regulatory subunit of the vacuolar transporter chaperone (VTC) complex			
**Regulatory**					
CBF1		Centromere binding factor 1; competes for Pho4 binding site motif CACGTG		-1.7	
PHO2		Transcription factor PHO2; constitutively expressed	2.2		X
PHO4		Transcription factor PHO4; constitutively expressed	1.7		
PHO81	X	Cyclin-dependent kinase (CDK) inhibitor; facilitates Pho4 translocation into the nucleus	1.6		
PHO85		Cyclin-dependent kinase (CDK)	1.7		X
PHO86		ER protein, regulates Pho84 trafficiking to PM		-1.7	
PHO92		Post-transcriptional regulation of Pho4 via Pi-dependent degradation of Pho4 mRNA			
SPL2		Suppressor of PLc1 deletion, regulatory protein	2.4		X
**Miscellaneous Phosphate Starvation Responsive**					
ENA1		P-type ATPase sodium pump; Na+ / Li+ efflux, salt tolerance	1.7		
PHM6		Protein of unknown function	3.3	-1.7	X
PHM7		Protein of unknown function		-1.8	X
**Inositol Metabolism****					
Inositol Synthesis, Cellular Uptake of Inositol					
INO1		Inositol-3-phosphate synthase	5.0	2.4	
ITR1		*myo*-Inositol transporter	1.8	2.2	
InsP Synthesis					
PLC1		Phospholipase C1; synthesis of inositol 1,4,5-triphosphate precursor of InsPPs	1.8		X
Regulation of Inositol Metabolism					
INO2			2.5		
InsPP Synthesis					
IPK1		Inositol Polyphosphate Kinase 1; phytate synthesis	2.1		X
IPMK		Inositol polyphosphate multikinase (IPMK), arginine-requiring; InsP5 synthesis			
KCS1		InsP6 and InsP7 kinase; important for phosphate signaling	1.5		
VIP1		Bifunctional inositol pyrophosphate kinase and phosphatase;; important for phosphate signaling			
InsPP Catabolism					
DDP1		Diadenosine and diphosphoinositol polyphosphate phosphatase; InsP7 hydrolysis			
SIW14		Synthetic Interaction with Whi2; hydrolyzes the ß-phosphate of 5-InsP7			

Supplementary Table S7. Some differentially-expressed genes in day 7 Arabidopsis *PS* and *DM* seedlings.

Gene	FC Expression		Symbol	Annotation
	PS	DM		
AT5G05060	41.9	27.1	MUG13.8	Cystatin/monellin superfamily protein; thiol proteinase inhibitors
AT1G75945	1.7	9.4		Uncharacterized protein
AT4G15670		2.4	GRXS7	Monothiol glutaredoxin-S7
AT1G08630		2.3	THA1	Threonine aldolase 1
AT2G19800		2.2	MIOX2	*myo* -inositol oxygenase 2; synctium formation
AT1G52770		2.1		Phototropic-responsive NPH3 family protein
AT1G75750	1.7	2.1	GASA1	Gibberellin-regulated protein 1
AT2G47270	2.0	2.1	UPB1	Transcription factor UPBEAT1; regulation of ROS, root development
AT3G57520		2.1	RFS2	Galactinol-sucrose galactosyltransferase 2
AT4G15680		2.1	GRXS4	Monothiol glutaredoxin-S4
AT3G30775		2.1	POX1	Proline dehydrogenase 1 ; EARLY RESPONSIVE TO DEHYDRATION 5, ERD5
AT1G54020		2.0		GDSL esterase/lipase
AT3G16770	2.0	2.0	RAP2-3	Ethylene-responsive transcription factor ; resistance to H2O2 and heat stresses
AT3G48520		2.0	CYP94B3	Cytochrome P450 CYP94B3; attenuates the jasmonic acid signaling
AT1G07050	1.8	2.0		CCT motif family protein
AT4G21650	-1.6	-2.0	SBT3.13	Subtilisin-like protease SBT3.13
AT4G15393	-1.8	-2.0	CYP702A5	Cytochrome P450
AT4G12735	1.6	-2.0		Unknown protein
AT1G73010	-2.0	-2.0	PS2	phosphate starvation-induced gene 2; Inorganic pyrophosphatase 1
AT2G04070		-2.1	DTX4	DETOXIFICATION 4
AT1G29395		-2.2	COR413IM1	COLD REGULATED 413 INNER MEMBRANE 1
AT5G50950		-2.2	FUM2	FUMARASE 2
AT3G51895		-2.3	SULTR3;1	Sulfate transporter 3.1
AT1G05680		-2.3	UGT74E2	UDP-glycosyltransferase 74E2; ROS and auxin signaling
AT5G13170	1.5	-2.5	SWEET15	Bidirectional sugar transporter; senescence-associated gene 29
AT2G18193		-2.8		AAA-ATPase
AT1G77220		-3.2		Protein of unknown function (DUF300); LAZ1 homolog 1
AT2G04050	-1.9	-4.1	DTX3	DETOXIFICATION 3

Supplementary Table S8. qRT-PCR verification of RNA-seq expression values for five selected DE genes in PS2- and DM4-expressing Arabidopsis. qRT-PCR expression values are determined by comparing expression levels to WT lines which is set to 1 for each gene tested. NDE indicates not-differentially-expressed (< 1.5-fold) in RNA-seq and qRT data for that gene. Student's t-test analysis shows *p<0.05, **p<0.01.

ATG ID	Symbol	Annotation	Fold-Change Expression			
			RNA-seq		qRT-PCR	
			PS	DM	PS	DM
At2g33770	PHO2	Phosphate 2, Ubiquitin-conjugating enzyme 24	NDE	-1.6	NDE	-2.63**
At2g04050	DTX3	Detoxification 3, Mate efflux family protein	-1.9	-4.1	-1.85*	-1.72**
At2g47270	UPB1	UPBEAT 1 transcription factor	2	2.1	5.31*	1.8
At1g05680	UGT74E2	Uridine diphosphate glycosyltransferase	NDE	-2.3	NDE	-1.85*
At1g75945	Unknown	Hypothetical protein	1.7	9.4	9.8**	55.4*

Supplementary Table S9. Differentially-expressed phosphate starvation-responsive genes in *PS* and *DM* plants. Fold-change (FC) expression values are given for each plant, relative to WT control plants.

Gene	PS	DM	Symbol	Description
AT4G02270	1.7		RHS13	Root hair specific 13
AT5G58010	1.6		LRL3	Lotus japonicus ROOTHAIRLESS1 (LjRHL1)-like 3
AT5G47740	1.5		AT5G47740	Adenine nucleotide alpha hydrolases-like superfamily protein
AT1G76430	-1.5		PHT1;9	Phosphate transporter 1;9
AT1G20620	-1.5		CAT3	Catalase-3
AT5G43370	-1.6		PHT1;2	Phosphate transporter 1;2
AT1G73010	-2.0	-2.0	PPA1	Inorganic pyrophosphatase 1
AT5G20410		-1.8	MGD2	Monogalactosyldiacylglycerol synthase 2; chloroplastic
AT2G22240		-1.7	IPS2	Inositol-3-phosphate synthase isozyme 2
AT2G33770		-1.6	PHO2, UBC24	Phosphate 2; ubiquitin-conjugating enzyme E2 24
AT3G23430		-1.5	PHO1	Phosphate transporter PHO1
AT1G14700		-1.5	PAP3	Purple acid phosphatase 3
AT1G13300		1.6	HRS1	HYPERSENSITIVITY TO LOW PI-ELICITED PRIMARY ROOT SHORTENING 1
AT3G20520		1.7	GDPDL5	Glycerophosphodiester phosphodiesterase-like 5
AT5G41080		1.8	GDPD2	Glycerophosphodiester phosphodiesterase 2

Supplementary Table S10. Summary information about PS and DM binding sites integrated with PS- and DM-specific gene expression in yeast. Nearest upstream and downstream genes from each binding site are identified. Upstream "sense" or downstream "antisense" gene orientations do not have the PS or DM binding site in their promoter, so corresponding gene expression values are assumed to be unrelated to PS or DM binding to the site. A 5'- or 3'-distance = "0" indicates partial or complete binding site overlap with the the gene.

Peak ID	Chromosome	Region	Center of peak	Length (bp)	Peak shape score	P-value	5' gene	5' distance	5'gene orientation	3' gene	3' distance	3' gene orientation	5' gene			3'gene		
													FC Expression		Annotation	FC Expression		Annotation
													PS	DM		PS	DM	
PS-Specific ChIP-Seq Binding SIte and PS-Specific Expression (5' Genes)																		
381300..381529_X	X	381300..381529	381,470	230	2.00	0.02	TAD2	298	antisense	KAR2	0	sense	2.7		tRNA-specific Adenosine Deaminase	2.4	-1.9	Hsp70 family ATPase KAR2
370062..370234_XVI	XVI	370062..370234	370,150	173	2.47	0.01	EEB1	620	antisense	SEC62	0	antisense	2.6		Ethyl Ester Biosynthesis	1.7		Sec63 complex subunit SEC62
550742..550890_IV	IV	550742..550890	550,807	149	3.52	0.00	BAP3	165	antisense	HEM12	969	sense	2.4		Branched-chain Amino acid Permease			HEMe biosynthesis; Uroporphyrinogen decarboxylase
682397..682660_II	II	682397..682660	682,554	264	3.11	0.00	LSR1	534	antisense	SWC5	0	antisense	2.4		SnRNAGene			Component of the SWR1 complex; complex exchanges histone variant H2AZ (Htz1p) for chromatin-bound histone H2A
958606..958795_IV	IV	958606..958795	958,737	190	1.86	0.03	YDR248C	266	antisense	YDR249C	0	antisense	2.2		gluconokinase	1.5		Putative protein of unknown function
797366..797624_XVI	XVI	797366..797624	797,558	259	2.08	0.02	SPN1	155	antisense	TOM5	0	sense	2.1		Suppresses Postrecruitment functions gene Number 1			Translocase of the Outer Mitochondrial membrane
818324..818530_XVI	XVI	818324..818530	818,474	207	2.89	0.00	KAR3	400	antisense	YPR142C	0	antisense	2.0		KARyogamy			Dubious open reading frame
818687..818860_XVI	XVI	818687..818860	818,788	174	3.20	0.00	KAR3	763	antisense	YPR142C	0	antisense	2.0		KARyogamy			Dubious open reading frame
73354..73485_XVI	XVI	73354..73485	73,433	132	2.62	0.00	VIK1	347	antisense	YAH1	0	antisense	2.0		Vegetative Interaction with Kar3p			Ferredoxin of the mitochondrial matrix
381318..381520_XIV	XIV	381318..381520	381,397	203	1.91	0.03	CPT1	486	antisense	DGR1	0	antisense	2.0		diacylglycerol cholinephosphotransferase			2-Deoxy-Glucose Resistant 1
626985..627226_XII	XII	626985..627226	627,155	242	2.03	0.02	CDD1	54	antisense	ERF2	0	sense	1.9		CytiDine Deaminase	-1.5	-1.7	palmitoyltransferase ERF2
497680..497914_IV	IV	497680..497914	497,756	235	2.34	0.01	VPS54	361	antisense	REG1	0	antisense	1.8		Vacuolar Protein Sorting	1.6		protein phosphatase regulator REG1
241566..241690_XVI	XVI	241566..241690	241,607	125	1.97	0.02	MLH3	68	antisense	SVS1	227	antisense	1.8		mismatch repair protein MLH3	1.9		Suppressor of Vanadate Sensitivity
242305..242454_XVI	XVI	242305..242454	242,390	150	2.19	0.01	MLH3	807	antisense	SVS1	0	antisense	1.8		mismatch repair protein MLH3	1.9		Suppressor of Vanadate Sensitivity
324524..324643_XVI	XVI	324524..324643	324,551	120	1.66	0.05	YPL119C-A	236	antisense	DBP1	0	antisense	1.8		Putative protein of unknown function	1.6		DEAD-box ATP-dependent RNA helicase DBP1
325008..325151_XVI	XVI	325008..325151	325,065	144	1.83	0.03	YPL119C-A	720	antisense	DBP1	0	antisense	1.8		Putative protein of unknown function	1.6		DEAD-box ATP-dependent RNA helicase DBP1
656760..656921_XVI	XVI	656760..656921	656,782	162	1.83	0.03	THP3	207	antisense	MCM16	0	sense	1.8		Protein THP3	1.9		Component of the Ctf19 complex and the COMA subcomplex
95281..95492_XVI	XVI	95281..95492	95,356	212	3.29	0.00	IQG1	171	antisense	CIN2	0	antisense	1.8		IQGAP-related protein	1.6		GTPase-activating protein CIN2
95870..96027_XVI	XVI	95870..96027	95,932	158	3.28	0.00	IQG1	760	antisense	CIN2	0	antisense	1.8		IQGAP-related protein	1.6		GTPase-activating protein CIN2
1204675..1204795_IV	IV	1204675..1204795	1,204,734	121	1.92	0.03	CDC40	464	antisense	ESF1	0	antisense	1.8		Cell Division Cycle; pre-mRNA splicing factor			Eighteen S rRNA Factor
1410745..1410994_IV	IV	1410745..1410994	1,410,901	250	2.40	0.01	JIP4	650	antisense	YDR476C	0	antisense	1.8		Jumonji domain Interacting Protein	1.7	1.9	Putative protein of unknown function
425995..426140_XVI	XVI	425995..426140	426,046	146	3.03	0.00	YPL067C	147	antisense	RGL1	92	sense	1.8		Histidine Triad with Channel			Regulator of Rho1p signaling, cofactor of Tus1p
827538..827696_IV	IV	827538..827696	827,598	159	3.09	0.00	SAS4	182	antisense	CDC1	0	sense	1.8		Something About Silencing	1.8		putative lipid phosphatase CDC1
1127826..1128030_IV	IV	1127826..1128030	1,127,901	205	3.18	0.00	PEX3	230	antisense	UBX5	0	sense	1.8		PEroXin			UBiquitin regulatory X
554418..554541_XVI	XVI	554418..554541	554,490	124	2.21	0.01	SNF8	89	antisense	HAT1	63	sense	1.8		Sucrose NonFermenting			Histone AcetylTransferase
225897..226078_XVI	XVI	225897..226078	225,968	182	2.00	0.02	COX10	155	antisense	OYE3	90	antisense	1.8		Cytochrome c OXidase	2.0		Old Yellow Enzyme; NADPH dehydrogenase ZRG6
693208..693356_XVI	XVI	693208..693356	693,283	149	2.07	0.02	LTP1	789	antisense	TKL1	0	antisense	1.7		Low molecular weight protein Tyrosine Phosphatase		1.5	transketolase TKL1
904113..904292_IV	IV	904113..904292	904,206	180	2.14	0.02	RAD9	632	antisense	SPR28	0	antisense	1.7		DNA repair protein RAD9	2.7	2.8	SPorulation Regulated
1055158..1055281_IV	IV	1055158..1055281	1,055,232	124	1.74	0.04	HDA2	510	antisense	MHR1	0	sense	1.7		Histone DeAcetylase HDA1 subunit			Mitochondrial ribosomal protein of the large subunit
1126270..1126506_IV	IV	1126270..1126506	1,126,372	237	2.02	0.02	SKP1	251	antisense	PEX3	0	antisense	1.7		Suppressor of Kinetochore Protein mutant	1.8		PEroXin
833606..833852_XVI	XVI	833606..833852	833,687	247	1.76	0.04	URN1	147	antisense	MAY24	0	sense	1.7		U2-U5-U6 snRNP, RES complex, and NTC interacting protein			Peripheral ER membrane protein
1072839..1073011_IV	IV	1072839..1073011	1,072,940	173	2.48	0.01	CPR5	281	antisense	HNT2	0	antisense	1.7		Cyclosporin-sensitive Proline Rotamase			bis(5'-adenosyl)-triphosphatase
1073480..1073618_IV	IV	1073480..1073618	1,073,546	139	3.10	0.00	CPR5	922	antisense	HNT2	0	antisense	1.7		Cyclosporin-sensitive Proline Rotamase			bis(5'-adenosyl)-triphosphatase
396632..396751_VII	VII	396632..396751	396,693	120	1.67	0.05	GEP7	802	antisense	SDS23	0	antisense	1.7		GEnetic interactors of Prohibitins			Protein involved in cell separation during budding
697817..697971_XVI	XVI	697817..697971	697,923	155	2.42	0.01	YPR077C	314	antisense	YPR078C	0	antisense	1.7		Dubious open reading frame	1.9	1.6	Putative protein of unknown function
1035614..1035790_IV	IV	1035614..1035790	1,035,693	177	1.84	0.03	YDR286C	38	antisense	INM2	204	sense	1.6		Mitochondrial Glutaredoxin-like Protein of 12 kDa			INositol Monophosphatase
408920..409165_XVI	XVI	408920..409165	409,076	246	2.16	0.02	ATP4	175	antisense	YPL077C	202	antisense	1.6		F1F0 ATP synthase subunit 4			Putative protein of unknown function
1093505..1093739_IV	IV	1093505..1093739	1,093,676	235	2.04	0.02	RAD34	993	antisense	IPK1	0	antisense	1.6		Homologous to RAD4	2.1		Inositol Polyphosphate Kinase 1
125561..125821_IV	IV	125561..125821	125,662	261	2.04	0.02	PPH22	562	antisense	YDL187C	0	antisense	1.6		protein phosphatase 2A, PP2A			Dubious open reading frame
346547..346752_XII	XII	346547..346752	346,643	206	1.75	0.04	CDC45	605	antisense	LCL2	0	sense	1.6		Cell Division Cycle; DNA replication initiation factor	1.6		Long Chronological Lifespan 2
1398265..1398528_IV	IV	1398265..1398528	1,398,357	264	2.07	0.02	YDR467C	355	antisense	TLG1	0	antisense	1.6		Dubious open reading frame			T-snare affecting a Late Golgi compartment
389117..389320_IV	IV	389117..389320	389,271	204	2.53	0.01	PUS9	215	antisense	GPR1	0	antisense	1.6		pseudouridine synthase PUS9	1.6		G-Protein coupled Receptor
389493..389677_IV	IV	389493..389677	389,565	185	2.51	0.01	PUS9	591	antisense	GPR1	0	antisense	1.6		pseudouridine synthase PUS9	1.6		G-Protein coupled Receptor
683630..683867_IV	IV	683630..683867	683,805	238	2.49	0.01	MRPL1	48	antisense	TMA64	78	antisense	1.6		mitochondrial 54S ribosomal protein MRPL1	1.8		Translation Machinery Associated
684419..684546_IV	IV	684419..684546	684,488	128	1.89	0.03	MRPL1	837	antisense	TMA64	0	antisense	1.6		mitochondrial 54S ribosomal protein MRPL1	1.8		Translation Machinery Associated
345296..345467_VII	VII	345296..345467	345,382	172	2.25	0.01	MF(ALPHA)2	142	antisense	YGL088W	368	sense	1.6		Mating Factor ALPHA		2.2	Putative protein of unknown function
557253..557466_IV	IV	557253..557466	557,376	214	2.39	0.01	TPI1	780	antisense	DET1	0	antisense	1.6		Triose-Phosphate Isomerase			Decreased Ergosterol Transport; Acid phosphatase
603587..603889_XVI	XVI	603587..603889	603,774	303	2.00	0.02	AGC1	229	antisense	SDD4	21	antisense	1.6		Aspartate-Glutamate Carrier			Suppressor of Degenerative Death
611894..612192_XV	XV	611894..612192	612,033	299	2.65	0.00	SMP3	505	antisense	MRPL23	0	sense	1.6		glycosylphosphatidylinositol-alpha 1,2 mannosyltransferase			mitochondrial 54S ribosomal protein YmL23
1297155..1297269_IV	IV	1297155..1297269	1,297,219	115	2.22	0.01	ERD1	468	antisense	YDR415C	0	antisense	1.6		Endoplasmic reticulum Retention Defective 1			Putative aminopeptidase
320200..320373_IV	IV	320200..320373	320,326	174	2.47	0.01	VAM6	79	antisense	RXT3	294	antisense	1.6		VAcuolar Morphogenesis			Component of the Rpd3L histone deacetylase complex; involved in histone deacetylation
765538..765746_IV	IV	765538..765746	765,626	209	2.18	0.01	CTH1	382	antisense	GIR2	0	sense	1.6		mRNA decay factor CTH1; iron homeostasis			Highly-acidic RWD domain-containing cytoplasmic protein
345629..345783_XVI	XVI	345629..345783	345,710	155	2.52	0.01	GDE1	889	antisense	YPL109C	0	antisense	1.5		GlycerophosphoDiEsterase 1			UbiB protein kinase-like family member; mitochondrial organization
514584..514790_XVI	XVI	514584..514790	514,675	207	2.81	0.00	ULP1	405	antisense	VTC3	0	antisense	1.5		UbL-specific Protease	2.1		Vacuolar Transporter Chaperone 3
98812..99131_XVI	XVI	98812..99131	99,016	320	2.53	0.01	HSP82	186	antisense	YAR1	352	sense	1.5		ATP-dependent molecular chaperone HSP82			Yeast Ankyrin Repeat
488363..488696_XIV	XIV	488363..488696	488,463	334	2.78	0.00	MLF3	238	antisense	MSK1	0	sense	1.5		Multicopy suppressor of LeFlunomide sensitivity			Mitochondrial aminoacyl-tRNA Synthetase, lysine (K)
826497..826735_XVI	XVI	826497..826735	826,573	239	2.70	0.00	YPR146C	820	antisense	YPR147C	0	antisense	1.5		Dubious open reading frame			Bifunctional triacylglycerol lipase and short chain ester hydrolase
225377..225694_XIV	XIV	225377..225694	225,484	318	2.13	0.02	CNM67	907	antisense	SQS1	0	antisense	1.5		Chaotic Nuclear Migration			Protein that stimulates the ATPase and helicase activities of Prp43p
192129..192398_VII	VII	192129..192398	192,215	270	2.72	0.00	YGL165C	152	antisense	YRB30	0	antisense	1.5		Dubious open reading frame	-1.5		Yeast Ran Binder
1114089..1114231_IV	IV	1114089..1114231	1,114,171	143	2.41	0.01	PEP7	61	antisense	UTP4	201	antisense	1.5		carboxyPEPtidase Y-deficient			Subunit of U3-containing 90S preribosome and SSU processome complexes
458609..458877_IV	IV	458609..458877	458,784	269	1.89	0.03	MAF1	505	antisense	SOK1	0	antisense	1.5		RNA polymerase III-inhibiting protein MAF1			Suppressor Of Kinase
1489080..1489268_IV	IV	1489080..1489268	1,489,151	189	3.07	0.00	**AGE1**	89	antisense	YDR524W-C	134	sense	1.5		Arf Gap with Effector function(s)	1.7		protein of unknown function
149002..149140_XIII	XIII	149002..149140	149,093	139	1.94	0.03	MFT1	318	antisense	PIF1	0	antisense	-1.5		THO complex subunit MFT1			DNA helicase PIF1
66269..66590_VIII	VIII	66269..66590	66,499	322	3.24	0.00	AIM17	409	antisense	OPI1	0	antisense	-1.5		Altered Inheritance rate of Mitochondria			OverProducer of Inositol; transcriptional regulator OPI1
66835..66933_VIII	VIII	66835..66933	66,874	99	2.12	0.02	AIM17	975	antisense	OPI1	0	antisense	-1.5		Altered Inheritance rate of Mitochondria			OverProducer of Inositol; transcriptional regulator OPI1
915186..915430_VII	VII	915186..915430	915,322	245	3.23	0.00	YGR210C	447	antisense	ZPR1	0	sense	-1.5		Putative protein of unknown function	-1.6		zinc finger-containing protein ZPR1
733712..734034_XV	XV	733712..734034	733,803	323	3.18	0.00	RET1	254	antisense	PTP2	0	sense	-1.5		DNA-directed RNA polymerase III core subunit RET1			tyrosine protein phosphatase PTP2
311066..311314_IV	IV	311066..311314	311,162	249	2.97	0.00	RPP1A	943	antisense	THI3	0	antisense	-1.5		Ribosomal Protein P1 Alpha			branched-chain-2-oxoacid decarboxylase THI3
962885..963058_VII	VII	962885..963058	962,969	174	2.05	0.02	SPG1	67	antisense	YGR237C	239	antisense	-1.5		Stationary Phase Gene			Putative protein of unknown function
280215..280375_V	V	280215..280375	280,282	161	1.80	0.04	CEM1	588	antisense	YER062C	0	antisense	-1.5		Mitochondrial beta-keto-acyl synthase		1.5	Glycerol-3-Phosphate Phosphatase
831140..831289_XV	XV	831140..831289	831,199	150	3.51	0.00	VPH1	565	antisense	FSF1	0	antisense	-1.5		H(+)-transporting V0 sector ATPase subunit a			Fungal SideroFlexin 1; alpha-isopropylmalate carrier
234974..235350_XII	XII	234974..235350	235,075	377	2.36	0.01	PDC1	892	antisense	STU2	0	antisense	-1.5		indolepyruvate decarboxylase 1			Microtubule polymerase of the XMAP215/Dis1 family
141958..142108_VIII	VIII	141958..142108	142,038	151	1.91	0.03	ARG4	555	antisense	DED81	0	antisense	-1.5		Argininosuccinate lyase	-1.9		Cytosolic asparaginyl-tRNA synthetase
539902..540111_XII	XII	539902..540111	540,059	210	1.90	0.03	HCR1	312	antisense	UPS1	0	antisense	-1.6		High-Copy suppressor of Rpg1			Phosphatidic acid transfer protein
540326..540512_XII	XII	540326..540512	540,360	187	1.79	0.04	HCR1	736	antisense	UPS1	0	antisense	-1.6		High-Copy suppressor of Rpg1			Phosphatidic acid transfer protein
83076..83285_V	V	83076..83285	83,178	210	5.74	0.00	RAD23	472	antisense	ANP1	0	antisense	-1.6		RADiation sensitive			Subunit of the alpha-1,6 mannosyltransferase complex; osmotic sensitivity
83557..83738_V	V	83557..83738	83,660	182	3.04	0.00	RAD23	953	antisense	ANP1	0	antisense	-1.6		RADiation sensitive			Subunit of the alpha-1,6 mannosyltransferase complex; osmotic sensitivity
497294..497471_VIII	VIII	497294..497471	497,351	178	3.23	0.00	AIM18	13	antisense	AIM46	18	antisense	-1.6		Altered Inheritance rate of Mitochondria			Altered Inheritance rate of Mitochondria
39422..39641_VIII	VIII	39422..39641	39,528	220	4.33	0.00	GUT1	913	antisense	GOS1	0	antisense	-1.6		glycerol kinase			v-SNARE protein involved in Golgi transport
730675..730831_XIV	XIV	730675..730831	730,729	157	2.87	0.00	HOL1	488	antisense	BIO5	786	antisense	-1.6		HistidinOl transporter	-2.2		Transmembrane protein involved in the biotin biosynthesis
714014..714204_XII	XII	714014..714204	714,111	191	1.79	0.04	RPS30A	855	antisense	MEC3	0	antisense	-1.6		ribosomal 40S subunit protein S30A			Mitosis Entry Checkpoint
16268..16469_XII	XII	16268..16469	16,373	202	2.78	0.00	AYT1	195	antisense	MHT1	169	antisense	-1.6		AcetYlTransferase			S-Methylmethionine-Homocysteine methylTransferase
16625..16814_XII	XII	16625..16814	16,721	190	3.08	0.00	AYT1	552	antisense	MHT1	0	antisense	-1.6		AcetYlTransferase			S-Methylmethionine-Homocysteine methylTransferase
375265..375515_VII	VII	375265..375515	375,406	251	5.07	0.00	RPB9	437	antisense	YGL069C	0	antisense	-1.6		RNA Polymerase B			Dubious open reading frame
189015..189275_IX	IX	189015..189275	189,198	261	2.99	0.00	RSM25	230	antisense	YIL092W	0	sense	-1.7		mitochondrial 37S ribosomal protein RSM25			Putative protein of unknown function
96749..96931_XV	XV	96749..96931	96,828	183	6.04	0.00	MCH4	387	antisense	YOL118C	0	antisense	-1.7		MonoCarboxylate transporter Homolog	-1.9		Putative protein of unknown function
696959..697094_XII	XII	696959..697094	697,040	136	2.30	0.01	DBP9	128	antisense	YSH1	61	antisense	-1.7		Dead Box Protein; ATP-dependent DNA/RNA helicase			Endoribonuclease YSH1
431737..431887_XV	XV	431737..431887	431,794	151	3.04	0.00	NOB1	110	antisense	SGT1	298	sense	-1.7		Nin1 (One) Binding protein			Suppressor of G2 (Two) allele of skp1; kinetochore complex assembly
286604..286778_XII	XII	286604..286778	286,703	175	2.53	0.01	BOS1	44	antisense	SIC1	41	sense	-1.8		Bet One Suppressor; v-SNARE (vesicle specific SNAP receptor)			cyclin-dependent protein serine/threonine kinase inhibiting protein SIC1
124817..125032_XIII	XIII	124817..125032	124,937	216	1.76	0.04	RPL6A	644	antisense	TCB3	0	antisense	-1.8		ribosomal 60S subunit protein L6A			Cortical ER protein involved in ER-plasma membrane tethering
296609..296878_XII	XII	296609..296878	296,696	270	3.57	0.00	EMP70	514	antisense	RAX2	0	antisense	-1.8		Protein with a role in cellular adhesion and filamentous growth			Protein required for the maintenance of bud site selection
61309..61583_II	II	61309..61583	61,404	275	2.40	0.01	RPL23A	569	antisense	YBL086C	0	antisense	-1.8		Ribosomal Protein of the Large subunit		-1.8	Protein of unknown function
271733..271971_V	V	271733..271971	271,796	239	2.73	0.00	HMF1	606	antisense	PET117	0	sense	-1.8		Homologous Mmf1p Factor; isoleucine biosynthesis			Assembly factor that couples heme a synthesis to complex IV assembly
788713..788890_VII	VII	788713..788890	788,795	178	3.03	0.00	RPL24B	933	antisense	GPC1	140	sense	-1.9		ribosomal 60S subunit protein L24B			GlyceroPhosphoCholine acyltransferase
415911..416188_IX	IX	415911..416188	415,974	278	3.07	0.00	DAL3	293	antisense	MGA2	0	sense	-1.9		Ureidoglycolate lyase			ER membrane protein involved in regulation of OLE1 transcription
349057..349192_XIV	XIV	349057..349192	349,124	136	2.88	0.00	RPC31	535	antisense	YNL150W	58	sense	-1.9		RNA polymerase III subunit C31			Dubious open reading frame
348620..348826_XIV	XIV	348620..348826	348,684	207	3.94	0.00	RPC31	98	antisense	YNL150W	424	sense	-1.9		RNA polymerase III subunit C31			Dubious open reading frame
338969..339121_XV	XV	338969..339121	339,046	153	2.82	0.00	TSR3	347	antisense	SGT2	0	antisense	-1.9		Twenty S rRNA accumulation			Glutamine-rich cytoplasmic cochaperone
265637..265871_IX	IX	265637..265871	265,736	235	1.81	0.04	YIL047C-A	169	antisense	SYG1	0	antisense	-2.0		Dubious open reading frame		-1.5	Suppressor of Yeast GPA1 (G Protein Alpha subunit)
806452..806686_VII	VII	806452..806686	806,547	235	5.42	0.00	MTR3	430	antisense	NSR1	0	antisense	-2.0		MRNA TRansport	-2.7		Nucleolin, nucleolar protein that binds nuclear localization sequences
284265..284467_VIII	VIII	284265..284467	284,392	203	1.65	0.05	GAR1	966	antisense	YNG2	0	antisense	-2.0		H/ACA snoRNP pseudouridylase subunit GAR1			histone acetyltransferase YNG2
76853..77016_X	X	76853..77016	76,949	164	2.17	0.01	BUD19	342	antisense	SWE1	0	antisense	-2.1		Dubious open reading frame		-2.0	tyrosine protein kinase SWE1
396484..396672_XIII	XIII	396484..396672	396,565	189	2.38	0.01	ARG7	104	antisense	RIM9	404	sense	-2.1		Mitochondrial ornithine acetyltransferase			Regulator of IME2
648962..649224_VII	VII	648962..649224	649,070	263	3.25	0.00	RPL11B	54	antisense	PIL1	373	antisense	-2.1		ribosomal 60S subunit protein L11B			Phosphorylation Inhibited by Long chain bases
634014..634350_XIV	XIV	634014..634350	634,211	337	3.73	0.00	ATO2	157	antisense	RPC34	0	antisense	-2.1		Ammonia (Ammonium) Transport Outward			RNA polymerase III subunit C34
634438..634713_XIV	XIV	634438..634713	634,609	276	2.06	0.02	ATO2	581	antisense	RPC34	0	antisense	-2.1		Ammonia (Ammonium) Transport Outward			RNA polymerase III subunit C34
223987..224123_III	III	223987..224123	224,047	137	1.68	0.05	PWP2	758	antisense	YIH1	0	antisense	-2.1		Periodic tryptophan (W) Protein			Negative regulator of eIF2 kinase Gcn2p
297887..298111_XIV	XIV	297887..298111	298,010	225	2.58	0.00	IPI3	259	antisense	PBR1	223	sense	-2.2		Involved in Processing ITS2; pre-rRNA-processing protein IPI3			Potentiates Bioactive compound Response; putative oxidoreductase
779707..779845_XIV	XIV	779707..779845	779,783	139	2.01	0.02	AIF1	968	antisense	COS10	70	sense	-2.3		Apoptosis-Inducing Factor			Endosomal protein involved in turnover of plasma membrane proteins
731274..731449_XIII	XIII	731274..731449	731,339	176	2.90	0.00	**RRP5**	150	antisense	RPS10B	964	sense	-2.3		Ribosomal RNA Processing	-1.6		ribosomal 40S subunit protein S10B
902655..902852_VII	VII	902655..902852	902,747	198	1.79	0.04	ELP2	384	antisense	YGR201C	0	antisense	-2.4		Elongator subunit ELP2			Putative protein of unknown function
897628..897762_XV	XV	897628..897762	897,695	135	2.97	0.00	YOR309C	549	antisense	NOP58	0	antisense	-2.8		Dubious open reading frame	-2.1		RNA-processing protein NOP58
PS-Specific ChIP-Seq Binding SIte and PS-Specific Expression (3' Genes)																		
95457..95639_XIV	XIV	95457..95639	95,555	183	2.25	0.01	CUS2	0	sense	YNL285W	533	sense			Cold sensitive U2 snRNA Suppressor	2.8		Dubious open reading frame
544303..544497_XIV	XIV	544303..544497	544,359	195	1.84	0.03	LAP2	0	sense	YIP3	770	sense			bifunctional aminopeptidase/epoxide hydrolase	2.1		Ypt-Interacting Protein; localized to COPII vesicles
1213501..1213629_IV	IV	1213501..1213629	1,213,584	129	1.91	0.03	KEI1	0	sense	YPR1	274	sense	2.0		Kex2-cleavable protein Essential for Inositol phosphorylceramide synthesis	2.1		trifunctional aldehyde reductase/carbonyl reductase (NADPH)/glucose 1-dehydrogenase (NADP(+)) YPR1
473329..473589_IV	IV	473329..473589	473,500	261	1.94	0.03	PSF1	0	sense	RAD61	456	sense	1.9		Partner of Sld Five	2.0		RADiation sensitive
874661..874880_XVI	XVI	874661..874880	874,794	220	2.20	0.01	MMS1	0	sense	RHO1	487	sense	2.0		Subunit of E3 ubiquitin ligase complex involved in replication repair	2.0		GTP-binding protein RHO1
669841..670084_XII	XII	669841..670084	669,901	244	2.07	0.02	TMA7	178	antisense	RED1	255	sense		1.7	Translation Machinery Associated	2.0		REDuctional division
703098..703352_VII	VII	703098..703352	703,227	255	4.10	0.00	YGR107W	0	sense	CLB1	283	sense			Dubious open reading frame	2.0		CycLin B
656760..656921_XVI	XVI	656760..656921	656,782	162	1.83	0.03	THP3	207	antisense	MCM16	0	sense	1.8		Protein THP3	1.9		Component of the Ctf19 complex and the COMA subcomplex
494902..495025_XVI	XVI	494902..495025	494,956	124	2.54	0.01	TRM44	0	sense	SUV3	480	sense			tRNA(Ser) Um(44) 2'-O-methyltransferase	1.9		ATP-dependent RNA helicase SUV3
461386..461595_IV	IV	461386..461595	461,454	210	2.77	0.00	SOK1	138	antisense	TRP1	246	sense			Suppressor Of Kinase	1.9		phosphoribosylanthranilate isomerase TRP1
472293..472535_IV	IV	472293..472535	472,378	243	1.77	0.04	RPL4B	0	sense	PSF1	621	sense		1.5	ribosomal 60S subunit protein L4B	1.9		Partner of Sld Five
769633..769881_IV	IV	769633..769881	769,725	249	3.22	0.00	RPA14	0	sense	YDR157W	49	sense			RNA polymerase I subunit A14	1.9		Putative protein of unknown function
787069..787277_XVI	XVI	787069..787277	787,136	209	2.61	0.00	CTR1	0	sense	YLH47	683	sense	1.5		high-affinity Cu transporter CTR1	1.9		Yeast LETM1 Homolog of 47 kD
841332..841627_XV	XV	841332..841627	841,495	296	2.20	0.01	RIM20	262	antisense	CAF20	0	sense			Regulator of IME2	1.8		Cap Associated Factor
1190042..1190272_IV	IV	1190042..1190272	1,190,126	231	3.22	0.00	CNL1	474	antisense	GGA1	0	sense	2.3	1.5	CNo-Like	1.8		Golgi-localized, Gamma-adaptin ear homology, Arf-binding protein
255214..255498_IV	IV	255214..255498	255,373	285	2.25	0.01	YDL114W-A	165	sense	YDL114W	105	sense			Dubious open reading frame	1.8		short-chain dehydrogenase/reductase
827538..827696_IV	IV	827538..827696	827,598	159	3.09	0.00	SAS4	182	antisense	CDC1	0	sense	1.8		Something About Silencing	1.8		putative lipid phosphatase CDC1
627723..627928_XVI	XVI	627723..627928	627,803	206	6.69	0.00	APL4	755	antisense	CSR2	0	sense			clathrin Adaptor Protein complex Large chain	1.8		Chs5 Spa2 Rescue
623308..623476_XVI	XVI	623308..623476	623,387	169	4.85	0.00	tF(GAA)P2	676	antisense	YOP1	50	sense			TRNAGene	1.8		YIP One Partner
34765..34929_XVI	XVI	34765..34929	34,825	165	3.90	0.00	KAR9	0	sense	PLC1	306	sense	1.8		KARyogamy; Spindle positioning factor	1.8		Phospholipase C1
580131..580262_IV	IV	580131..580262	580,187	132	2.12	0.02	RPS13	0	sense	RRG1	424	sense	-1.5		ribosomal 40S subunit protein S13	1.7		Required for Respiratory Growth
774283..774569_XIII	XIII	774283..774569	774,450	287	4.63	0.00	GTO3	267	sense	HOR7	182	sense	1.7		Glutathione Transferase Omega-like	1.7		HyperOsmolarity-Responsive
231089..231334_XVI	XVI	231089..231334	231,165	246	3.40	0.00	MEX67	250	antisense	MRX4	0	sense			mRNA export factor MEX67	1.7		Mitochondrial oRganization of gene eXpression (MIOREX)
1489080..1489268_IV	IV	1489080..1489268	1,489,151	189	3.07	0.00	**AGE1**	89	antisense	**YDR524W-C**	134	sense	1.5		Arf Gap with Effector function(s)	1.7		protein of unknown function
165362..165727_XVI	XVI	165362..165727	165,609	366	2.14	0.02	HRR25	0	sense	TPK2	528	sense			Conserved casein kinase, monopolin subunit	1.7		cAMP-dependent protein kinase catalytic subunit TPK2
228238..228418_IV	IV	228238..228418	228,330	181	2.48	0.01	LYS21	0	sense	STF1	752	sense			Homocitrate synthase isozyme; lysine biosynthesis	1.7		ATPase-binding protein; regulation of the mitochondrial F1F0-ATP synthase
1383578..1383771_IV	IV	1383578..1383771	1,383,631	194	1.97	0.02	PFA5	134	antisense	TFB3	39	sense			palmitoyltransferase PFA5	1.7		Transcription Factor B subunit 3
830004..830192_XVI	XVI	830004..830192	830,076	189	1.75	0.04	NCE102	0	sense	YPR150W	806	sense	2.0	-1.8	NonClassical Export	1.7		Dubious open reading frame
906220..906413_XVI	XVI	906220..906413	906,341	194	1.98	0.02	GDB1	0	sense	ATG13	804	sense			bifunctional 4-alpha-glucanotransferase/amylo-alpha-1,6-glucosidase	1.7		Regulatory subunit of the Atg1p signaling complex
1103500..1103692_IV	IV	1103500..1103692	1,103,621	193	3.20	0.00	HIM1	71	sense	MCM21	65	sense			High Induced Mutagenesis	1.6		Component of the kinetochore sub-complex COMA
494484..494811_XV	XV	494484..494811	494,672	328	4.57	0.00	TMA46	13	sense	ECM3	315	sense			Translation Machinery Associated	1.6		putative ATPase ECM3
346547..346752_XII	XII	346547..346752	346,643	206	1.75	0.04	CDC45	605	antisense	LCL2	0	sense	1.6		Cell Division Cycle; DNA replication initiation factor	1.6		Long Chronological Lifespan 2
345351..345557_IV	IV	345351..345557	345,467	207	2.88	0.00	RAD59	397	antisense	USO1	107	sense		-2.2	RADiation sensitive; Protein involved DNA double-strand break repair	1.6		yUSOu - transport in Japanese; involved in vesicle-mediated ER to Golgi transport
345000..345154_IV	IV	345000..345154	345,093	155	2.86	0.00	RAD59	46	antisense	USO1	510	sense		-2.2	RADiation sensitive; Protein involved DNA double-strand break repair	1.6		yUSOu - transport in Japanese; involved in vesicle-mediated ER to Golgi transport
1020502..1020607_IV	IV	1020502..1020607	1,020,554	106	1.82	0.03	RNH202	81	sense	RRP45	139	sense	1.6		RNase H	1.6		exosome non-catalytic core subunit RRP45
165062..165284_XV	XV	165062..165284	165,191	223	2.13	0.02	PHM7	0	sense	ATG34	429	sense		-1.8	PHosphate Metabolism 7	1.6		Receptor protein involved in selective autophagy during starvation
1009071..1009192_IV	IV	1009071..1009192	1,009,124	122	2.02	0.02	GLO2	0	sense	DON1	983	sense			Cytoplasmic glyoxalase II	1.6		DONut; meiosis-specific component of the spindle pole body
314589..314810_II	II	314589..314810	314,673	222	1.90	0.03	CHS2	0	sense	ATP3	764	sense	2.3		CHitin Synthase II	1.6		Gamma subunit of the F1 sector of mitochondrial F1F0 ATP synthase
597767..597941_IV	IV	597767..597941	597,876	175	1.96	0.02	PPH3	0	sense	RAD55	526	sense			Catalytic subunit of protein phosphatase PP4 complex	1.6		RADiation sensitive; putative DNA-dependent ATPase RAD55
1130805..1130954_IV	IV	1130805..1130954	1,130,885	150	4.35	0.00	GPI8	0	sense	IRC3	46	sense	2.5		GlycosylPhosphatidylInositol anchor biosynthesis	1.6		double-stranded DNA-dependent ATPase
1398870..1399078_IV	IV	1398870..1399078	1,398,916	209	1.70	0.04	TLG1	169	antisense	SDC1	0	sense			T-snare affecting a Late Golgi compartment	1.6		Set1c, homolog of Dpy30 from C.elegans
817092..817252_IV	IV	817092..817252	817,153	161	2.43	0.01	UBC1	0	sense	SDH4	697	sense			E2 ubiquitin-conjugating protein UBC1	1.6		Succinate DeHydrogenase
1369503..1369712_IV	IV	1369503..1369712	1,369,595	210	2.92	0.00	NHX1	116	sense	TOM1	77	sense			Na+/H+ eXchanger	1.6		E3 ubiquitin-protein ligase TOM1
1260672..1260816_IV	IV	1260672..1260816	1,260,739	145	2.02	0.02	SHE9	0	sense	RPT3	864	sense	1.7		Sensitivity to High Expression	1.6		proteasome regulatory particle base subunit RPT3
1145411..1145650_IV	IV	1145411..1145650	1,145,501	240	2.48	0.01	MRX8	0	sense	MRPS28	668	sense			Mitochondrial oRganization of gene eXpression (MIOREX)	1.5		mitochondrial 54S ribosomal protein YmL49
596521..596632_XVI	XVI	596521..596632	596,584	112	2.68	0.00	RLF2	224	sense	MCM4	117	sense	2.1		Rap1 protein Localization Factor	1.5		MCM DNA helicase complex subunit MCM4
264680..264946_XVI	XVI	264680..264946	264,825	267	2.76	0.00	YPL152W-A	0	sense	RRD2	81	sense			Protein of unknown function	1.5		peptidylprolyl isomerase RRD2
885213..885312_XVI	XVI	885213..885312	885,263	100	1.71	0.04	BSP1	0	sense	YPR172W	483	sense	1.7		Binding protein of Synaptojanin Polyphosphoinositide phosphatase domain	1.5		pyridoxal 5'-phosphate synthase
1339150..1339325_IV	IV	1339150..1339325	1,339,216	176	2.85	0.00	THI74	0	sense	LRS4	350	sense	1.5		Mitochondrial transporter repressible by thiamine	1.5		Loss of RDNA Silencing
380331..380545_II	II	380331..380545	380,477	215	2.13	0.02	ALG14	396	antisense	YBR071W	0	sense			Component of UDP-GlcNAc transferase	1.5		Protein of unknown function found in the cytoplasm and bud neck
1273995..1274271_IV	IV	1273995..1274271	1,274,072	277	3.59	0.00	DIT2	291	antisense	DIT1	330	sense		3.2	N-formyltyrosine oxidase	1.5		Sporulation-specific enzyme required for spore wall maturation
859033..859185_IV	IV	859033..859185	859,106	153	3.30	0.00	COQ4	0	sense	MSC2	160	sense			COenzyme Q	1.5		Meiotic Sister-Chromatid recombination
1086624..1086711_IV	IV	1086624..1086711	1,086,673	88	1.77	0.04	TFB1	0	sense	SSF2	869	sense			TFIIH/NER complex subunit TFB1	1.5		rRNA-binding ribosome biosynthesis protein
808864..809107_XII	XII	808864..809107	808,947	244	2.38	0.01	SPO77	45	sense	FKS1	889	sense			Meiosis-specific protein of unknown function	-1.5		1,3-beta-D-glucan synthase
131528..131674_IX	IX	131528..131674	131,629	147	2.30	0.01	POG1	0	sense	QDR2	569	sense			Transcriptional activator POG1	-1.5		cation transporter QuiniDine Resistance
663396..663552_XIV	XIV	663396..663552	663,508	157	2.67	0.00	TIM23	0	sense	RCF2	717	sense			Translocase of the Inner Mitochondrial membrane	-1.5		Cytochrome c oxidase subunit
622499..622619_XIV	XIV	622499..622619	622,556	121	2.31	0.01	MRP7	70	antisense	HRB1	295	sense			mitochondrial 54S ribosomal protein YmL2	-1.5		Poly(A+) RNA-binding protein
131356..131472_VII	VII	131356..131472	131,409	117	2.72	0.00	DSD1	186	sense	GCN1	52	sense		-1.9	D-serine ammonia-lyase DSD1	-1.5		Positive regulator of the Gcn2p kinase activity

260693..260887_XII	XII	260693..260887	260,803	195	2.28	0.01	REX2	145	antisense	FRS1	91	sense			3'-5' RNA exonuclease	-1.5		phenylalanyl (F)-tRNA Synthetase
98694..98805_XI	XI	98694..98805	98,749	112	2.35	0.01	YKL183C-A	86	antisense	LOT5	0	sense			Putative protein of unknown function	-1.5		LOw Temperature-responsive
516951..517110_VIII	VIII	516951..517110	517,041	160	1.81	0.04	SET5	465	antisense	BAT1	421	sense	-1.7	-2.0	S-adenosylmethionine-dependent methyltransferase	-1.5		Branched-chain Amino acid Transaminase
516564..516788_VIII	VIII	516564..516788	516,649	225	2.33	0.01	SET5	78	antisense	BAT1	743	sense	-1.7	-2.0	S-adenosylmethionine-dependent methyltransferase	-1.5		Branched-chain Amino acid Transaminase
578702..579056_IV	IV	578702..579056	578,954	355	3.06	0.00	AIM7	0	sense	RPS13	401	sense	1.5		Altered Inheritance rate of Mitochondria	-1.5		ribosomal 40S subunit protein S13
427360..427628_IV	IV	427360..427628	427,439	269	6.90	0.00	TSC13	425	antisense	NOP1	0	sense			Enoyl reductase; very long chain fatty acid elongation	-1.5		rRNA methyltransferase NOP1
637143..637383_XIII	XIII	637143..637383	637,255	241	2.84	0.00	MRPS17	138	antisense	GCV2	116	sense			mitochondrial 37S ribosomal protein MRPS17	-1.5		glycine decarboxylase subunit P
461169..461294_X	X	461169..461294	461,218	126	2.39	0.01	GPI14	0	sense	TMA22	534	sense		-1.8	GlycosylPhosphatidylInositol anchor biosynthesis	-1.5		Translation Machinery Associated
58792..59087_V	V	58792..59087	58,887	296	2.06	0.02	VMA8	0	sense	YEL050W-A	461	sense			H(+)-transporting V1 sector ATPase subunit D	-1.5		Dubious open reading frame
651008..651149_X	X	651008..651149	651,074	142	2.45	0.01	IBA57	0	sense	RPS5	751	sense		-1.9	Iron-sulfur cluster assembly factor for Biotin synthase and Aconitase-like mitochondrial pro	-1.6		ribosomal 40S subunit protein S5
490163..490284_VIII	VIII	490163..490284	490,227	122	1.97	0.02	MDM31	0	sense	NVJ1	462	sense			Mitochondrial Distribution and Morphology	-1.6		Nucleus-Vacuole Junction
89098..89325_VII	VII	89098..89325	89,256	228	2.61	0.00	CLG1	0	sense	YGL214W	684	sense			Cyclin-like protein that interacts with Pho85p	-1.6		Dubious open reading frame
898380..898508_XII	XII	898380..898508	898,453	129	2.35	0.01	REH1	707	antisense	RPS29A	142	sense			Cytoplasmic 60S subunit biogenesis factor	-1.6		ribosomal 40S subunit protein S29A
227253..227568_II	II	227253..227568	227,382	316	2.89	0.00	tS(AGA)B	96	sense	UTP20	67	sense			TRNAGene	-1.6		rRNA-processing protein UTP20
915186..915430_VII	VII	915186..915430	915,322	245	3.23	0.00	YGR210C	447	antisense	ZPR1	0	sense	-1.5		Putative protein of unknown function	-1.6		zinc finger-containing protein ZPR1
73649..73808_XIII	XIII	73649..73808	73,710	160	1.78	0.04	TSL1	0	sense	YML099W-A	420	sense	-1.5	-2.1	Trehalose Synthase Long chain	-1.6		Dubious open reading frame
73181..73334_XIII	XIII	73181..73334	73,252	154	2.20	0.01	TSL1	0	sense	YML099W-A	894	sense	-1.5	-2.1	Trehalose Synthase Long chain	-1.6		Dubious open reading frame
105212..105531_VIII	VIII	105212..105531	105,412	320	2.42	0.01	RPL14B	120	sense	OSH7	523	sense	-2.0		ribosomal 60S subunit protein L14B	-1.6		OxySterol binding protein Homolog
284626..284793_XIII	XIII	284626..284793	284,691	168	1.96	0.02	ADI1	0	sense	ANY1	306	sense	-1.5		Acireductone DIoxygenase	-1.6		Antagonizes Neo1 Yeast phospholipid flippase
731274..731449_XIII	XIII	731274..731449	731,339	176	2.90	0.00	**RRP5**	150	antisense	**RPS10B**	964	sense	-2.3		Ribosomal RNA Processing	-1.6		ribosomal 40S subunit protein S10B
331592..331749_II	II	331592..331749	331,657	158	3.25	0.00	ZTA1	80	antisense	FMP23	83	sense		-1.5	NADPH-dependent quinone reductase	-1.6		Found in Mitochondrial Proteome
58857..59104_III	III	58857..59104	58,939	248	4.19	0.00	SRO9	178	antisense	GFD2	0	sense	-1.5	1.8	Cytoplasmic RNA-binding protein	-1.6		Great for Full DEAD box protein activity
693178..693306_VII	VII	693178..693306	693,241	129	2.92	0.00	MDR1	80	sense	PCP1	56	sense			Cytoplasmic GTPase-activating protein	-1.7		rhomboid protease PCP1; mitochondrial
52902..53106_XV	XV	52902..53106	52,954	205	1.79	0.04	CTR9	113	antisense	NOP8	0	sense			Component of the Paf1p complex involved in transcription elongation	-1.7		NucleOlar Protein; required for 60S ribosomal subunit biogenesis
1010274..1010538_XV	XV	1010274..1010538	1,010,473	265	1.94	0.03	HAP5	0	sense	VTS1	650	sense			Heme Activator Protein; Subunit of the Hap2p/3p/4p/5p CCAAT-binding complex	-1.7		VTi1-2 Suppressor; mRNA degradation
86251..86473_XII	XII	86251..86473	86,340	223	2.31	0.01	TPO1	0	sense	ISA1	929	sense	-1.9		Polyamine transporter 1	-1.7		Fe-binding Fe/S cluster assembly protein ISA1
90897..91238_III	III	90897..91238	91,157	342	2.74	0.00	tL(CAA)C	0	sense	LEU2	85	sense			TRNAGene	-1.7		3-isopropylmalate dehydrogenase
101813..101955_XIII	XIII	101813..101955	101,871	143	2.32	0.01	YML083C	762	antisense	YML082W	0	sense			Protein of unknown function	-1.7		putative cystathionine gamma-synthase
314084..314246_VII	VII	314084..314246	314,178	163	2.02	0.02	SEH1	0	sense	LSG1	384	sense			Subunit of the Nup84 nuclear pore and SEACAT subcomplexes	-1.7		Large-Subunit Gtpase
49251..49468_XII	XII	49251..49468	49,318	218	1.98	0.02	YLL044W	588	sense	FPS1	469	sense			Dubious open reading frame	-1.8		Aquaglyceroporin, plasma membrane channel
439474..439638_VII	VII	439474..439638	439,569	165	2.08	0.02	RPL30	0	sense	CGR1	424	sense	-1.5	1.8	ribosomal 60S subunit protein L30	-1.8		Coiled-coil Growth-Regulated
360966..361295_VIII	VIII	360966..361295	361,244	330	3.18	0.00	YHR127W	0	sense	FUR1	819	sense			Protein of unknown function	-1.8		Uracil phosphoribosyltransferase
373728..373899_XI	XI	373728..373899	373,825	172	2.02	0.02	TUL1	0	sense	YKL033W-A	605	sense			Subunit of the DSC ubiquitin ligase complex	-1.9		putative hydrolase
352357..352520_XIII	XIII	352357..352520	352,456	164	2.43	0.01	tF(GAA)M	0	sense	ARG80	82	sense			TRNAGene	-1.9		Transcription factor involved in regulating arginine-responsive genes
84118..84379_XII	XII	84118..84379	84,257	262	4.76	0.00	FRA1	407	sense	TPO1	424	sense			aminopeptidase P	-1.9		Polyamine transporter 1
393350..393516_VIII	VIII	393350..393516	393,437	167	4.00	0.00	MRPL6	66	antisense	IMP3	17	sense			mitochondrial 54S ribosomal protein YmL16	-1.9		snoRNA-binding rRNA-processing protein IMP3
214700..214849_XIV	XIV	214700..214849	214,782	150	2.77	0.00	BNI4	99	sense	CSL4	73	sense			Targeting subunit for Glc7p protein phosphatase	-1.9		exosome non-catalytic core subunit CSL4
675065..675261_XIV	XIV	675065..675261	675,211	197	1.91	0.03	BUD17	0	sense	CPR8	915	sense			Putative pyridoxal kinase	-1.9		Cyclosporin-sensitive Proline Rotamase
82194..82440_VI	VI	82194..82440	82,341	247	3.09	0.00	GYP8	281	antisense	STE2	139	sense			Gtpase-activating protein for Ypt Proteins	-1.9		alpha-factor pheromone receptor STE2
410232..410382_IX	IX	410232..410382	410,295	151	2.60	0.00	DAL4	0	sense	DAL2	424	sense	-5.0	-4.5	Allantoin permease	-2.0		Allantoicase; degradation of allantoin
103726..103886_VIII	VIII	103726..103886	103,792	161	1.99	0.02	HSE1	0	sense	RPL14B	390	sense			Subunit of the endosomal Vps27p-Hse1p complex	-2.0		ribosomal 60S subunit protein L14B
127321..127519_XII	XII	127321..127519	127,422	199	3.25	0.00	YEH1	65	sense	SOF1	3	sense			Yeast steryl Ester Hydrolase	-2.2		rRNA-processing protein SOF1
505076..505215_XII	XII	505076..505215	505,149	140	2.39	0.01	IDP2	0	sense	CBF5	918	sense		1.5	Isocitrate Dehydrogenase, NADP-specific	-2.2		pseudouridine synthase CBF5
727319..727437_XV	XV	727319..727437	727,367	119	2.67	0.00	GEP3	84	antisense	NOC2	74	sense			Protein required for mitochondrial ribosome small subunit biogenesis	-2.3		mRNA-binding ribosome synthesis protein NOC2
383129..383323_X	X	383129..383323	383,222	195	1.86	0.03	KAR2	0	sense	HCA4	513	sense	2.4	-1.9	Hsp70 family ATPase KAR2	-2.4		RNA-dependent ATPase HCA4
557244..557446_XVI	XVI	557244..557446	557,310	203	2.00	0.02	CIT3	0	sense	PDH1	938	sense		1.8	CItrate synthase	-2.4		putative 2-methylcitrate dehydratase
431521..431777_XI	XI	431521..431777	431,652	257	3.69	0.00	snR87	382	antisense	RPL14A	128	sense			SnoRNAGene	-2.5		ribosomal 60S subunit protein L14A
**DM-Specific ChIP-Seq Binding SIte and DM-Specific Expression (5' Gene)**																		
77966..78205_III	III	77966..78205	78,101	240	1.90	0.03	AGP1	46	antisense	YCL023C	743	antisense		4.6	high-Affinity Glutamine Permease			Dubious open reading frame
781061..781214_VII	VII	781061..781214	781,126	154	2.64	0.00	THI4	0	sense	ENP2	552	sense		3.2	THIamine metabolism; Thiazole synthase	-1.9		ribosome biosynthesis protein ENP2
554166..554459_XII	XII	554166..554459	554,298	294	2.68	0.00	HMX1	488	antisense	ENT2	118	sense		2.4	HeMe oXygenase	1.7		Epsin N-Terminal homology
436364..436506_II	II	436364..436506	436,417	143	2.21	0.01	YBR096W	0	sense	VPS15	444	sense		2.4	Putative protein of unknown function	1.6		Vacuolar Protein Sorting 15
436772..436905_II	II	436772..436905	436,835	134	1.79	0.04	YBR096W	58	sense	VPS15	45	sense		2.4	Putative protein of unknown function	1.6		Vacuolar Protein Sorting 15
287968..288122_XV	XV	287968..288122	288,040	155	2.53	0.01	TAT2	17	sense	tY(GUA)O	69	sense		2.2	High affinity tryptophan and tyrosine permease			TRNAGene
720839..721007_II	II	720839..721007	720,924	169	1.78	0.04	SPO23	234	sense	MRPS5	382	sense		2.0	Protein of unknown function; SPOrulation			mitochondrial 37S ribosomal protein MRPS5
153772..153991_II	II	153772..153991	153,842	220	2.19	0.01	POL12	161	antisense	STU1	0	antisense		1.9	DNA-directed DNA polymerase alpha subunit POL12			Microtubule plus-end-tracking non-motor protein
55231..55471_VI	VI	55231..55471	55,381	241	1.66	0.05	ACT1	534	antisense	YPT1	0	antisense		1.9	ACTin	1.5		Rab family GTPase YPT1
98967..99094_XV	XV	98967..99094	99,039	128	1.77	0.04	RRI2	0	sense	MSN1	714	sense		1.9	Subunit of the COP9 signalosome (CSN) complex		1.6	Multicopy suppressor of SNF1 mutation
301268..301426_II	II	301268..301426	301,328	159	1.85	0.03	RPL4A	13	sense	YBR032W	92	sense		1.8	Ribosomal 60S subunit protein L4A		1.5	Putative protein of unknown function
119750..119984_XIII	XIII	119750..119984	119,860	235	2.49	0.01	HMG1	851	antisense	FPR3	104	antisense		1.8	3-Hydroxy-3-MethylGlutaryl-coenzyme a reductase			Nucleolar peptidyl-prolyl cis-trans isomerase (PPIase)
498075..498245_VII	VII	498075..498245	498,149	171	3.95	0.00	EFM5	40	antisense	SWC4	230	antisense		1.8	Elongation Factor Methyltransferase			Component of the Swr1p complex that incorporates Htz1p into chromatin
47971..48130_VII	VII	47971..48130	48,054	160	1.74	0.04	KAP114	0	sense	DOC1	482	sense		1.8	KAryoPherin			Destruction Of Cyclin B
592535..592695_VII	VII	592535..592695	592,635	161	1.79	0.04	YGR050C	92	antisense	YGR051C	210	antisense		1.8	Protein of unknown function			Dubious open reading frame
160879..161040_XV	XV	160879..161040	160,961	162	3.57	0.00	ADH1	284	antisense	YOL085W-A	538	sense		1.7	alcohol dehydrogenase ADH1			Dubious open reading frame
21093..21274_VIII	VIII	21093..21274	21,181	182	3.08	0.00	ARN1	121	antisense	EFM1	508	sense		1.7	ARN family transporter for siderophore-iron chelates	-1.8		Elongation Factor Methyltransferase
738895..739135_XIII	XIII	738895..739135	738,975	241	2.19	0.01	FUS2	0	sense	TRI1	49	sense		1.7	cell FUSion regulator			Non-essential sumoylated protein of unknown function
1161341..1161458_IV	IV	1161341..1161458	1,161,397	118	2.88	0.00	HXT6	20	antisense	YDR344C	547	antisense		1.7	hexose transporter HXT6			Putative protein of unknown function
1162011..1162246_IV	IV	1162011..1162246	1,162,093	236	2.64	0.00	HXT6	690	antisense	YDR344C	0	antisense		1.7	hexose transporter HXT6			Putative protein of unknown function
226400..226573_XV	XV	226400..226573	226,448	174	1.93	0.03	THI20	324	antisense	tG(GCC)O1	37	antisense		1.7	trifunctional hydroxymethylpyrimidine kinase/phosphomethylpyrimidine kinase/thiaminase			TRNAGene
930550..930781_IV	IV	930550..930781	930,665	232	4.34	0.00	RTN1	192	antisense	LYS4	347	sense		1.6	ReTiculoN-like; nuclear pore assembly			Homoaconitas; lysine biosynthesis
128622..128848_XV	XV	128622..128848	128,724	227	2.65	0.00	IZH4	701	antisense	PKH2	388	sense		1.6	Implicated in Zinc Homeostasis			serine/threonine protein kinase PKH2
747149..747297_XII	XII	747149..747297	747,217	149	2.23	0.01	YLR307C-A	37	antisense	CDA2	639	sense		1.6	Delta-Psi dependent mitochondrial Assembly protein of 10 kDa			Chitin DeAcetylase
614282..614518_XII	XII	614282..614518	614,381	237	2.73	0.00	THI7	118	sense	FAR10	236	sense		1.6	thiamine transporter THI7			rotein involved in recovery from arrest in response to pheromone
853839..854010_XII	XII	853839..854010	853,933	172	2.26	0.01	YLR363W-A	120	sense	GRX8	51	sense		1.6	Protein of unknown function		-1.6	glutathione-disulfide reductase
231243..231460_XII	XII	231243..231460	231,305	218	2.30	0.01	YLR042C	791	antisense	TRX1	241	antisense		1.6	Negative regulator of the Filamentous Growth MAPK pathway			ThioRedoXin
921831..922002_VII	VII	921831..922002	921,884	172	2.04	0.02	RPS0A	42	sense	RSM27	172	sense		1.6	Ribosomal Protein of the Small subunit			mitochondrial 37S ribosomal protein RSM27
209377..209525_V	V	209377..209525	209,446	149	3.32	0.00	CHO1	902	antisense	GAL83	0	antisense		1.6	Phosphatidylserine synthase			One of three alternate beta-subunits of the Snf1 kinase complex
383515..383662_XIV	XIV	383515..383662	383,570	148	1.67	0.05	TEP1	0	sense	FAR11	324	sense		1.6	putative phosphatidylinositol-3,4,5-trisphosphate 3-phosphatase			Protein involved in recovery from cell cycle arrest
139825..139958_V	V	139825..139958	139,910	134	2.04	0.02	GCN4	61	antisense	YEL008W	553	sense		1.6	General control transcription factor GCN4			Putative protein of unknown function
270880..271012_V	V	270880..271012	270,959	133	2.03	0.02	RPL34A	694	antisense	HMF1	0	antisense		1.6	Ribosomal 60S subunit protein L34A	-1.8		Homologous Mmf1p Factor; isoleucine biosynthesis
46456..46594_V	V	46456..46594	46,527	139	3.13	0.00	SDD1	734	antisense	**HAT2**	573	sense		1.5	Suppressor of Degenerative Death		-1.5	Histone AcetylTransferase
448229..448525_XI	XI	448229..448525	448,472	297	1.79	0.04	ECM9	192	antisense	YKR005C	0	antisense		1.5	ExtraCellular Mutant			Putative protein of unknown function
535224..535430_XIV	XIV	535224..535430	535,367	207	1.73	0.04	YNL050C	241	antisense	SFB2	0	antisense		1.5	Putative protein of unknown function			COPII subunit SFB2
703271..703486_IV	IV	703271..703486	703,424	216	2.55	0.01	SWF1	0	sense	ARO1	997	sense		1.5	Spore Wall Formation			Pentafunctional arom protein; catalyzes steps 2 through 6 in the biosynthesis of chorismate
505338..505471_XII	XII	505338..505471	505,381	134	1.74	0.04	IDP2	0	sense	CBF5	662	sense		1.5	Isocitrate Dehydrogenase, NADP-specific	-2.2		pseudouridine synthase CBF5
213716..213994_XI	XI	213716..213994	213,865	279	2.56	0.01	SRP21	717	antisense	DGR2	147	sense		1.5	Signal Recognition Particle			2-Deoxy-Glucose Resistant 2
818285..818402_XII	XII	818285..818402	818,330	118	4.41	0.00	GAS2	523	sense	tR(CCG)L	206	sense		1.5	1,3-beta-glucanosyltransferase			TRNAGene
169872..170061_II	II	169872..170061	169,987	190	2.19	0.01	RPL19B	495	sense	LSM2	561	sense		1.5	Ribosomal 60S subunit protein L19B			Sm-like protein LSM2; degradation of cytoplasmic mRNAs
122256..122522_XIV	XIV	122256..122522	122,376	267	2.83	0.00	GOR1	85	antisense	TOF1	360	sense		-1.5	GlyOxylate Reductase			TOpoisomerase I-interacting Factor
194016..194366_XII	XII	194016..194366	194,292	351	1.87	0.03	UBR2	734	antisense	SNF7	85	sense		-1.5	putative ubiquitin-protein ligase UBR2			ESCRT-III subunit protein SNF7
31712..31957_XIII	XIII	31712..31957	31,830	246	2.64	0.00	YML119W	27	sense	NGL3	376	sense		-1.5	Putative protein of unknown function			3'-5' exonuclease specific for poly-A RNAs
658340..658642_XV	XV	658340..658642	658,553	303	2.95	0.00	DCS2	14	sense	MED4	104	sense		-1.5	5'-(N(7)-methyl 5'-triphosphoguanosine)-(mRNA) diphosphatase		1.9	Mediator of RNA polymerase II transcription subunit 4
371464..371675_XI	XI	371464..371675	371,588	212	3.21	0.00	UGP1	73	sense	TUL1	153	sense		-1.5	UDP-glucose pyrophosphorylase			Subunit of the DSC ubiquitin ligase complex
280221..280372_XIV	XIV	280221..280372	280,303	152	2.96	0.00	CHS1	323	sense	DUG3	59	sense		-1.5	CHitin Synthase			Component of glutamine amidotransferase (GATase II)
179531..179752_XIII	XIII	179531..179752	179,700	222	1.66	0.05	GSF2	0	sense	YML047W-A	140	sense		-1.5	Glucose Signaling Factor			Dubious open reading frame
483911..484136_IV	IV	483911..484136	484,050	226	1.67	0.05	YDR018C	50	antisense	GCV1	26	antisense		-1.5	putative acyltransferase			glycine decarboxylase subunit T
57664..57867_I	I	57664..57867	57,810	204	1.77	0.04	BOL3	278	antisense	YAL045C	0	antisense		-1.6	BolA-like protein		-2.3	Dubious open reading frame
319112..319436_XVI	XVI	319112..319436	319,377	325	1.75	0.04	RNY1	166	antisense	TFB2	0	antisense		-1.6	ribonuclease T2			TFIIH/NER complex subunit TFB2
137148..137516_III	III	137148..137516	137,247	369	2.92	0.00	ADP1	274	antisense	PGK1	229	sense		-1.6	ATP-Dependent Permease, ABC transporter			3-PhosphoGlycerate Kinase
291046..291244_X	X	291046..291244	291,149	199	2.47	0.01	PRY1	271	antisense	PRY3	91	antisense		-1.6	Pathogen Related in Yeast	-1.5	-2.9	GPI-anchored cell wall protein involved in export of sterols
263830..263983_V	V	263830..263983	263,898	154	2.56	0.01	GIP2	131	antisense	HIS1	908	antisense		-1.6	Putative regulatory subunit of protein phosphatase Glc7p			ATP phosphoribosyltransferase; histidine biosynthesis
649160..649317_X	X	649160..649317	649,226	158	2.52	0.01	ATP2	17	sense	IBA57	458	sense		-1.6	Beta subunit of the F1 sector of mitochondrial F1F0 ATP synthase		-1.9	Iron-sulfur cluster assembly factor for Biotin synthase and Aconitase-like mitochondrial proteins
138474..138687_I	I	138474..138687	138,537	214	1.70	0.04	ERP2	128	antisense	TRN1	464	sense		-1.6	Emp24p/Erv25p Related Protein	3.0		TRNAGene
519350..519476_X	X	519350..519476	519,421	127	4.69	0.00	VPS55	164	antisense	SSC1	161	antisense		-1.7	Vacuolar Protein Sorting		-1.8	Hsp70 family ATPase SSC1
684061..684224_X	X	684061..684224	684,160	164	2.36	0.01	MET5	775	antisense	**IML1**	342	sense		-1.7	METhionine requiring; Sulfite reductase beta subunit		-2.2	GTPase-activating protein IML1
364769..364909_X	X	364769..364909	364,838	141	1.90	0.03	MHP1	0	sense	NSP1	993	sense		-1.7	MAP-Homologous Protein		-1.5	FG-nucleoporin NSP1
307689..307833_XIV	XIV	307689..307833	307,754	145	2.11	0.02	TDA7	708	antisense	NOP13	0	antisense		-1.7	Topoisomerase I Damage Affected		1.6	NucleOlar Protein; preribosomal complexes
32134..32277_V	V	32134..32277	32,222	144	1.66	0.05	AVT2	894	antisense	CAN1	0	antisense		-1.7	Amino acid Vacuolar Transport			CANavanine resistance
581695..581916_X	X	581695..581916	581,844	222	1.70	0.04	AIM24	78	antisense	EAF6	0	antisense		-1.7	Altered Inheritance rate of Mitochondria			Subunit of the NuA4 acetyltransferase complex
333809..334073_X	X	333809..334073	334,013	265	1.80	0.04	YJL055W	0	sense	TIM54	191	sense		-1.7	LOnely Guy; mediates resistance to 5-FU		-2.0	Translocase of the Inner Mitochondrial membrane
70723..70967_X	X	70723..70967	70,859	245	2.50	0.01	CDC6	0	sense	YJL193W	398	sense		-1.7	Cell Division Cycle		-1.6	Putative protein of unknown function
226677..226866_X	X	226677..226866	226,790	190	2.21	0.01	SET4	0	sense	PAM16	460	sense		-1.7	SET domain-containing;regulates response to oxidative stress		-1.5	import motor complex subunit PAM16
336873..337110_VII	VII	336873..337110	337,044	238	1.93	0.03	SPC105	0	sense	NUP145	795	sense		-1.8	Spindle Pole Component	-1.7		nucleocytoplasmic transporter NUP145
91413..91628_XII	XII	91413..91628	91,493	216	2.71	0.00	HSP104	63	sense	tP(UGG)L	919	sense		-1.8	chaperone ATPase HSP104	2.0		TRNAGene
319267..319454_X	X	319267..319454	319,363	188	2.58	0.01	LAS21	0	sense	NUP82	561	sense		-1.8	Mannose-ethanolamine phosphotransferase			Linker nucleoporin component of the nuclear pore complex (NPC)
238877..239019_X	X	238877..239019	238,941	143	2.21	0.01	LSB6	0	sense	CHS6	394	sense		-1.8	1-phosphatidylinositol 4-kinase LSB6		-1.8	CHitin Synthase-related
106286..106644_XV	XV	106286..106644	106,559	359	2.16	0.02	SKM1	0	sense	MSB4	65	sense		-1.8	STE20/PAK homologous Kinase related to Morphogenesis			GTPase-activating protein of the Ras superfamily
74164..74366_III	III	74164..74366	74,293	203	3.12	0.00	HBN1	177	antisense	FRM2	337	antisense		-1.8	Homologous to Bacterial Nitroreductases		-2.3	Type II nitroreductase, using NADH as reductant
594645..594909_X	X	594645..594909	594,797	265	2.07	0.02	GRR1	317	antisense	JSN1	151	antisense		-1.8	Glucose Repression-Resistant		-2.6	RNA-binding protein Just Say No
604763..604884_X	X	604763..604884	604,825	122	2.06	0.02	FIP1	559	antisense	IME1	0	antisense		-1.9	Factor Interacting with Poly(A) polymerase		-2.9	Inducer of MEiosis; master regulator of early meiotic genes
83126..83355_X	X	83126..83355	83,215	230	1.76	0.04	ATG36	149	antisense	**GON7**	92	sense		-1.9	AuTophaGy related		-1.7	chromatin DNA-binding EKC/KEOPS complex subunit GON7|LDB6|PCC2
105996..106176_I	I	105996..106176	106,072	181	3.22	0.00	LTE1	123	antisense	PMT2	95	antisense		-1.9	Low Temperature Essential		-2.0	Protein O-MannosylTransferase
94483..94674_I	I	94483..94674	94,563	192	2.01	0.02	FRT2	0	sense	SAW1	12	sense		-2.0	Functionally Related to TCP1		-1.7	DNA-binding protein SAW1
68399..68661_I	I	68399..68661	68,500	263	2.30	0.01	CLN3	878	antisense	CYC3	54	antisense		-2.0	CycLiN 3		-2.2	Cytochrome c heme lyase (holocytochrome c synthase)
130810..131032_X	X	130810..131032	130,925	223	2.42	0.01	FBP26	166	antisense	VPS35	68	antisense		-2.1	Fructose-2,6-bisphosphatase, required for glucose metabolism		-2.1	Vacuolar Protein Sorting 35
75422..75546_III	III	75422..75546	75,491	125	1.75	0.04	FRM2	136	antisense	AGP1	471	antisense		-2.3	Type II nitroreductase, using NADH as reductant		4.6	high-Affinity Glutamine Permease
691560..691911_XIV	XIV	691560..691911	691,647	352	1.92	0.03	SOL1	273	sense	EGO4	649	sense		-2.4	Suppressor Of Los1-1	1.6		Exit from rapamycin-induced GrOwth arrest
759725..759938_XIV	XIV	759725..759938	759,803	214	2.44	0.01	DSE4	625	antisense	YNR068C	128	antisense		-2.5	endo-1,3(4)-beta-glucanase			Putative protein of unknown function
48332..48517_I	I	48332..48517	48,405	186	2.55	0.01	FLC2	81	sense	OAF1	46	sense		-2.6	flavin adenine dinucleotide transporter FLC2		-3.0	Oleate-Activated transcription Factor
77015..77226_IV	IV	77015..77226	77,126	212	3.94	0.00	PRR2	469	antisense	NOP6	62	antisense		-2.6	Pheromone Response Regulator			rRNA-binding protein required for 40S ribosomal subunit biogenesis
157610..158016_III	III	157610..158016	157,748	407	2.01	0.02	HSP30	502	antisense	YCR022C	0	antisense		-3.3	Heat Shock Protein			Putative protein of unknown function
**DM-Specific ChIP-Seq Binding SIte and DM-Specific Expression (3' Gene)**																		
1058813..1059203_XV	XV	1058813..1059203	1,059,091	391	3.94	0.00	YOR381W-A	222	sense	FIT2	327	sense			Putative protein of unknown function		3.3	Facilitator of Iron Transport
856115..856277_XII	XII	856115..856277	856,223	163	1.73	0.04	YLR366W	271	sense	RPS22B	164	sense			Dubious open reading frame		2.7	ribosomal 40S subunit protein S22B
472534..472698_VIII	VIII	472534..472698	472,620	165	4.83	0.00	GND1	104	sense	SSP1	45	sense			6-phosphoGlucoNateDehydrogenase		2.3	SPO3; involved in the control of meiotic nuclear division
322281..322490_V	V	322281..322490	322,382	210	3.29	0.00	SRG1	0	sense	SER3	195	sense			ncRNA gene		2.0	phosphoglycerate dehydrogenase SER3
152075..152198_II	II	152075..152198	152,133	124	2.43	0.01	YBL036C	854	antisense	POL12	0	antisense			Putative non-specific single-domain racemase		1.9	DNA-directed DNA polymerase alpha subunit POL12
336278..336504_XIII	XIII	336278..336504	336,370	227	1.91	0.03	HOF1	0	sense	YMR030W-A	808	sense			F-BAR protein that regulates actin cytoskeleton organization		1.9	Putative protein of unknown function
658340..658642_XV	XV	658340..658642	658,553	303	2.95	0.00	DCS2	14	sense	MED4	104	sense		-1.5	5'-(N(7)-methyl 5'-triphosphoguanosine)-(mRNA) diphosphatase		1.9	Mediator of RNA polymerase II transcription subunit 4
71473..71696_III	III	71473..71696	71,620	224	1.97	0.02	RNQ1	105	sense	FUS1	106	sense			[PIN(+)] prion		1.9	cell FUSion
721032..721166_XII	XII	721032..721166	721,094	135	2.04	0.02	SEC72	661	antisense	GSP1	0	antisense			Sec63 complex subunit SEC72		1.9	Genetic Suppressor of Prp20-1
203368..203573_IX	IX	203368..203573	203,464	206	3.44	0.00	SDS3	108	antisense	CAB2	0	antisense			Suppressor of Defective Silencing; Component of the Rpd3L histone deacetylase complex		1.8	Phosphopantothenoylcysteine synthetase (PPCS)
355643..355822_VIII	VIII	355643..355822	355,720	180	1.75	0.04	EPT1	0	sense	NDT80	738	sense			EthanolaminePhosphoTransferase		1.8	Meiosis-specific transcription factor NDT80
70577..70816_XV	XV	70577..70816	70,629	240	1.88	0.03	HRT1	0	sense	GAS4	483	sense			High level expression Reduces Ty3 transposition		1.7	1,3-beta-glucanosyltransferase
71198..71403_II	II	71198..71403	71,258	206	1.82	0.03	ALG3	70	antisense	YBL081W	462	sense			Dolichol-P-Man dependent alpha(1-3) mannosyltransferase		1.7	Protein of unknown function; non-essential
141720..141923_III	III	141720..141923	141,830	204	1.95	0.03	POL4	786	antisense	CTO1	0	antisense			DNA polymerase IV		1.6	Cold TOlerance
534839..535103_XIII	XIII	534839..535103	534,923	265	2.06	0.02	RRB1	140	antisense	JLP2	0	antisense	-2.1		Regulator of Ribosome Biogenesis		1.6	dnaJ-Like Protein
98967..99094_XV	XV	98967..99094	99,039	128	1.77	0.04	RRI2	0	sense	MSN1	714	sense		1.9	Subunit of the COP9 signalosome (CSN) complex		1.6	Multicopy suppressor of SNF1 mutation
853240..853455_XII	XII	853240..853455	853,357	216	2.44	0.01	NMD4	88	antisense	YLR363W-A	5	sense			Nonsense-Mediated mRNA Decay		1.6	Protein of unknown function
412940..413185_XIII	XIII	412940..413185	412,998	246	1.83	0.03	IRC21	66	antisense	SDD2	0	antisense			Increased Recombination Centers		1.6	Suppressor of Degenerative Death
848490..848669_VII	VII	848490..848669	848,580	180	6.00	0.00	ERG1	66	antisense	YGR176W	50	sense			ERGosterol biosynthesis; Squalene epoxidase		1.6	Dubious open reading frame
112224..112404_XV	XV	112224..112404	112,285	181	2.07	0.02	YOL107W	0	sense	YOL106W	821	sense	1.5		Putative protein of unknown function		1.6	Dubious open reading frame
567926..568107_XII	XII	567926..568107	568,032	182	4.12	0.00	CRR1	3	antisense	FRE1	459	sense			Putative glycoside hydrolase of the spore wall envelope		1.6	Ferric REductase
268755..268936_V	V	268755..268936	268,863	182	9.03	0.00	FCY2	641	antisense	RPL34A	486	antisense	-1.7	-1.9	FluoroCYtosine resistance		1.6	Ribosomal 60S subunit protein L34A
307689..307833_XIV	XIV	307689..307833	307,754	145	2.11	0.02	TDA7	708	antisense	NOP13	0	antisense		-1.7	Topoisomerase I Damage Affected		1.6	NucleOlar Protein; preribosomal complexes
183585..183848_IX	IX	183585..183848	183,713	264	3.67	0.00	tI(AAU)I1	71	antisense	PRK1	88	sense			TRNAGene		1.5	p53 Regulatory Kinase
937821..937956_VII	VII	937821..937956	937,898	136	1.68	0.05	MRPL9	937	antisense	TOS2	0	antisense			mitochondrial 54S ribosomal protein YmL9		1.5	Target Of Sbf
210656..210899_XV	XV	210656..210899	210,821	244	2.37	0.01	CRT10	390	antisense	APM4	0	antisense			Constitutive RNR Transcription regulators		1.5	clathrin Adaptor Protein complex Medium chain
220328..220516_VI	VI	220328..220516	220,438	189	1.80	0.04	SMC2	221	antisense	RPL2A	0	antisense			condensin subunit SMC2		1.5	ribosomal 60S subunit protein L2A
574732..575000_XV	XV	574732..575000	574,904	269	4.00	0.00	VPS17	0	sense	EFT1	97	sense			Vacuolar Protein Sorting; Subunit of the membrane-associated retromer complex		1.5	Elongation Factor Two
279728..279868_V	V	279728..279868	279,802	141	3.01	0.00	CEM1	101	antisense	YER062C	61	antisense	-1.5		Mitochondrial beta-keto-acyl synthase		1.5	Glycerol-3-Phosphate Phosphatase
172742..172894_XIII	XIII	172742..172894	172,816	153	1.93	0.03	GAL80	0	sense	AIM32	244	sense			Transcriptional repression of GAL genes		1.5	Altered Inheritance rate of Mitochondria
634417..634564_II	II	634417..634564	634,482	148	1.78	0.04	KTR3	0	sense	YBR206W	36	sense	-1.5		mannosyltransferase KTR3		1.5	Dubious open reading frame
1359174..1359420_IV	IV	1359174..1359420	1,359,314	247	2.71	0.00	UTP6	271	antisense	RPS18A	502	sense			component of the small subunit (SSU) processome involved in processing of pre-18S rRNA		1.5	ribosomal 40S subunit protein S18A
301268..301426_II	II	301268..301426	301,328	159	1.85	0.03	RPL4A	13	sense	YBR032W	92	sense		1.8	Ribosomal 60S subunit protein L4A		1.5	Putative protein of unknown function
842405..842578_XV	XV	842405..842578	842,450	174	1.97	0.02	snR5	0	sense	HEM4	238	sense	1.5	2.3	SnoRNAGene		1.5	Uroporphyrinogen III synthase; HEMe biosynthesis
463515..463752_XI	XI	463515..463752	463,639	238	3.75	0.00	YKR011C	818	antisense	YKR012C	0	antisense	1.5		Protein of unknown function		1.5	Dubious open reading frame
773017..773256_II	II	773017..773256	773,188	240	1.68	0.05	YBR284W	0	sense	YBR285W	665	sense	1.8		metallo-dependent hydrolase superfamily protein		-1.5	Putative protein of unknown function
46456..46594_V	V	46456..46594	46,527	139	3.13	0.00	**SDD1**	734	antisense	HAT2	573	sense		1.5	Suppressor of Degenerative Death		-1.5	Histone AcetylTransferase
187854..188197_XII	XII	187854..188197	188,058	344	2.58	0.00	IZH3	724	antisense	UBR2	0	antisense			Implicated in Zinc Homeostasis		-1.5	putative ubiquitin-protein ligase UBR2
1153680..1153868_IV	IV	1153680..1153868	1,153,774	189	2.48	0.01	YDR341C	53	antisense	HXT7	347	antisense			Arginyl-tRNA synthetase		-1.5	hexose transporter HXT7
466957..467085_X	X	466957..467085	467,007	129	2.69	0.00	ILV3	748	antisense	ESS1	0	antisense	-2.1	-1.6	Dihydroxyacid dehydratase		-1.5	Peptidylprolyl-cis/trans-isomerase (PPIase)
5112..5226_XV	XV	5112..5226	5,152	115	1.72	0.04	YOL164W-A	799	sense	BDS1	948	sense			Putative protein of unknown function		-1.5	Bacterially Derived Sulfatase
226677..226866_X	X	226677..226866	226,790	190	2.21	0.01	SET4	0	sense	PAM16	460	sense		-1.7	SET domain-containing;regulates response to oxidative stress		-1.5	import motor complex subunit PAM16
790698..790861_XIII	XIII	790698..790861	790,795	164	1.71	0.04	TIF11	858	antisense	TPS3	0	antisense	-1.6		Translation initiation factor eIF1A		-1.5	Trehalose Phosphate Synthase
656705..656876_XV	XV	656705..656876	656,788	172	3.40	0.00	YRM1	134	sense	DCS2	387	sense			Zinc finger transcription factor involved in multidrug resistance		-1.5	5'-(N(7)-methyl 5'-triphosphoguanosine)-(mRNA) diphosphatase
405824..406004_XIII	XIII	405824..406004	405,894	181	2.32	0.01	UBX4	250	antisense	AVO2	299	sense			UBiquitin regulatory X		-1.5	Adheres VOraciously (to TOR2)
364769..364909_X	X	364769..364909	364,838	141	1.90	0.03	MHP1	0	sense	NSP1	993	sense		-1.7	MAP-Homologous Protein		-1.5	FG-nucleoporin NSP1
266042..266188_IX	IX	266042..266188	266,107	147	2.11	0.02	YIL047C-A	574	antisense	SYG1	0	antisense	-2.0		Dubious open reading frame		-1.5	Suppressor of Yeast GPA1 (G Protein Alpha subunit)
145429..145615_X	X	145429..145615	145,524	187	2.47	0.01	SFH5	0	sense	YJL144W	743	sense	-1.5	-1.6	Unusual phosphatidylinositol transfer protein (PITP) that binds heme		-1.5	Ub-ligase substrate-specificity factor
43304..43472_XV	XV	43304..43472	43,402	169	1.81	0.04	FRE7	693	sense	GRE2	221	sense			putative ferric-chelate reductase		-1.5	Genes de Respuesta a Estres (stress responsive genes)

565939..566175_XI	XI	565939..566175	566,006	237	2.91	0.00	PAM17	46	antisense	CCP1	0	antisense			Presequence translocase-Associated Motor		-1.5	Cytochrome c Peroxidase
471109..471319_XIV	XIV	471109..471319	471,246	211	2.82	0.00	END3	6	antisense	SAL1	57	sense			EH domain-containing protein involved in endocytosis		-1.6	Ca(2+)-binding ATP:ADP antiporter
493527..493791_XII	XII	493527..493791	493,660	265	2.39	0.01	YLR163W-A	601	sense	SHH4	91	sense			Dubious open reading frame		-1.6	succinate dehydrogenase SDH4 Homolog
568319..568542_XIII	XIII	568319..568542	568,442	224	1.85	0.03	RIM13	136	antisense	YMR155W	8	sense			Calpain-like cysteine protease		-1.6	Putative protein of unknown function
853839..854010_XII	XII	853839..854010	853,933	172	2.26	0.01	YLR363W-A	120	sense	GRX8	51	sense		1.6	Protein of unknown function		-1.6	glutathione-disulfide reductase
70723..70967_X	X	70723..70967	70,859	245	2.50	0.01	CDC6	0	sense	YJL193W	398	sense		-1.7	Cell Division Cycle		-1.6	Putative protein of unknown function
374980..375192_IX	IX	374980..375192	375,136	213	1.77	0.04	MSL1	119	sense	DSN1	238	sense			U2B component of U2 snRNP		-1.6	Dosage Suppressor of NNF1
633006..633359_X	X	633006..633359	633,124	354	3.22	0.00	CPA2	64	antisense	YMR1	255	sense			Carbamyl Phosphate synthetase A		-1.6	phosphatidylinositol-3-phosphatase YMR1
273578..273725_V	V	273578..273725	273,639	148	1.99	0.02	PCL6	0	sense	FCY21	841	sense			Pho85 CycLin 6		-1.6	purine-cytosine permease
261780..262115_V	V	261780..262115	262,016	336	2.89	0.00	YER053C-A	733	antisense	GIP2	0	antisense			Protein of unknown function		-1.6	Putative regulatory subunit of protein phosphatase Glc7p
327946..328153_X	X	327946..328153	328,049	208	1.76	0.04	BIT61	72	antisense	IKS1	0	antisense			Binding partner of Tor2p		-1.6	protein kinase IKS1
328671..328840_X	X	328671..328840	328,755	170	2.32	0.01	BIT61	797	antisense	IKS1	0	antisense			Binding partner of Tor2p		-1.6	protein kinase IKS1
792251..792450_II	II	792251..792450	792,369	200	2.70	0.00	SUL1	436	sense	PCA1	398	sense	-2.9		High affinity sulfate permease		-1.6	Cadmium transporting P-type ATPase
246073..246223_X	X	246073..246223	246,162	151	2.01	0.02	PHS1	0	sense	MRPL49	266	sense			Essential 3-hydroxyacyl-CoA dehydratase of the ER membrane; Sphingolipid biosynthesis 1		-1.7	Mitochondrial Ribosomal Protein, Large subunit
540303..540488_XIII	XIII	540303..540488	540,398	186	3.97	0.00	YMR135W-A	0	sense	GAT2	710	sense			Dubious open reading frame		-1.7	Protein containing GATA family zinc finger motifs; similar to Gln3p and Dal80p
678980..679186_X	X	678980..679186	679,085	207	2.83	0.00	TTI2	273	antisense	MET5	0	antisense			Two Tel2-Interacting protein; chromatin remodeling		-1.7	METhionine requiring; Sulfite reductase beta subunit
330549..330684_XV	XV	330549..330684	330,620	136	1.88	0.03	ALG6	0	sense	YSP3	770	sense			ER-luminal alpha 1,3 glucosyltransferase		-1.7	Yeast Subtilisin-like Protease III
83126..83355_X	X	83126..83355	83,215	230	1.76	0.04	**ATG36**	149	antisense	GON7	92	sense		-1.9	AuTophaGy related		-1.7	chromatin DNA-binding EKC/KEOPS complex subunit GON7|LDB6|PCC2
94483..94674_I	I	94483..94674	94,563	192	2.01	0.02	FRT2	0	sense	SAW1	12	sense		-2.0	Functionally Related to TCP1		-1.7	DNA-binding protein SAW1
136307..136553_I	I	136307..136553	136,392	247	1.76	0.04	SPO7	0	sense	FUN14	360	sense	-1.5	-1.8	Nem1-Spo7 phosphatase regulatory subunit SPO7		-1.7	Integral mitochondrial outer membrane (MOM) protein
473349..473518_XIII	XIII	473349..473518	473,427	170	2.33	0.01	YMR103C	84	antisense	YPK2	0	antisense	-1.6	-2.0	Putative protein of unknown function		-1.8	putative protein kinase YPK2
317007..317208_X	X	317007..317208	317,063	202	1.80	0.04	COA3	26	sense	LAS21	75	sense			Cytochrome Oxidase Assembly		-1.8	Mannose-ethanolamine phosphotransferase
349456..349720_V	V	349456..349720	349,537	265	3.04	0.00	PUP3	109	antisense	RAD51	259	sense			proteasome core particle subunit beta 3		-1.8	recombinase RAD51
396868..397057_X	X	396868..397057	396,969	190	5.05	0.00	tG(GCC)J1	71	antisense	PET130	297	antisense			TRNAGene		-1.8	PETite colonies
397381..397561_X	X	397381..397561	397,476	181	1.94	0.03	tG(GCC)J1	584	antisense	PET130	0	antisense			TRNAGene		-1.8	PETite colonies
238877..239019_X	X	238877..239019	238,941	143	2.21	0.01	LSB6	0	sense	CHS6	394	sense		-1.8	1-phosphatidylinositol 4-kinase LSB6		-1.8	CHitin Synthase-related
519350..519476_X	X	519350..519476	519,421	127	4.69	0.00	VPS55	164	antisense	SSC1	161	antisense		-1.7	Vacuolar Protein Sorting		-1.8	Hsp70 family ATPase SSC1
190560..190701_XIV	XIV	190560..190701	190,635	142	2.14	0.02	SLA2	0	sense	ATG2	622	sense			Adaptor protein that links actin to clathrin and endocytosis		-1.9	Peripheral membrane protein required for autophagic vesicle formation
582457..582616_X	X	582457..582616	582,534	160	2.16	0.02	EAF6	201	antisense	ACF4	0	antisense			Subunit of the NuA4 acetyltransferase complex		-1.9	Assembly Complementing Factor
656382..656591_X	X	656382..656591	656,468	210	2.09	0.02	ENT3	418	antisense	VPS70	0	antisense			Epsin N-Terminal homology		-1.9	Vacuolar Protein Sorting 70
1065886..1066020_XV	XV	1065886..1066020	1,065,945	135	1.89	0.03	YOR385W	0	sense	PHR1	818	sense			Putative protein of unknown function		-1.9	deoxyribodipyrimidine photo-lyase PHR1
649160..649317_X	X	649160..649317	649,226	158	2.52	0.01	ATP2	17	sense	IBA57	458	sense		-1.6	Beta subunit of the F1 sector of mitochondrial F1F0 ATP synthase		-1.9	Iron-sulfur cluster assembly factor for Biotin synthase and Aconitase-like mitochondrial proteins
128938..129064_VII	VII	128938..129064	128,988	127	1.82	0.03	MDS3	0	sense	DSD1	818	sense			Mck1 Dosage Suppressor; Putative component of the TOR regulatory pathway		-1.9	D-serine ammonia-lyase DSD1
228710..229054_X	X	228710..229054	228,804	345	2.86	0.00	snR37	230	antisense	GSM1	0	antisense			SnoRNAGene		-2.0	Glucose Starvation Modulator
105996..106176_I	I	105996..106176	106,072	181	3.22	0.00	LTE1	123	antisense	PMT2	95	antisense		-1.9	Low Temperature Essential		-2.0	Protein O-MannosylTransferase
333809..334073_X	X	333809..334073	334,013	265	1.80	0.04	YJL055W	0	sense	TIM54	191	sense		-1.7	LOnely Guy; mediates resistance to 5-FU		-2.0	Translocase of the Inner Mitochondrial membrane
486628..486804_X	X	486628..486804	486,698	177	2.60	0.00	RBH2	431	antisense	GEA1	0	antisense			Ran Binding domain Homolog		-2.0	Guanine nucleotide Exchange on ARF
62918..63050_X	X	62918..63050	62,993	133	1.88	0.03	PHO90	0	sense	UBP12	754	sense	-2.4	-2.1	SPX domain-containing inorganic phosphate transporter		-2.0	UBiquitin-specific Protease UBP12
130810..131032_X	X	130810..131032	130,925	223	2.42	0.01	FBP26	166	antisense	VPS35	68	antisense		-2.1	Fructose-2,6-bisphosphatase, required for glucose metabolism		-2.1	Vacuolar Protein Sorting 35
458764..458938_X	X	458764..458938	458,854	175	2.03	0.02	SPC1	402	antisense	YJR011C	0	antisense	2.2		Signal Peptidase Complex; Subunit of spindle-assembly checkpoint complex		-2.2	Putative protein of unknown function
684061..684224_X	X	684061..684224	684,160	164	2.36	0.01	**MET5**	775	antisense	IML1	342	sense		-1.7	METhionine requiring; Sulfite reductase beta subunit		-2.2	GTPase-activating protein IML1
68399..68661_I	I	68399..68661	68,500	263	2.30	0.01	CLN3	878	antisense	CYC3	54	antisense		-2.0	CycLiN 3		-2.2	Cytochrome c heme lyase (holocytochrome c synthase)
74164..74366_III	III	74164..74366	74,293	203	3.12	0.00	HBN1	177	antisense	FRM2	337	antisense		-1.8	Homologous to Bacterial Nitroreductases		-2.3	Type II nitroreductase, using NADH as reductant
274151..274337_II	II	274151..274337	274,227	187	11.02	0.00	KAP104	447	antisense	GAL7	89	antisense	-1.6		Transportin or cytosolic karyopherin beta 2		-2.3	Galactose-1-phosphate uridyl transferase
274632..274819_II	II	274632..274819	274,767	188	2.26	0.01	KAP104	928	antisense	GAL7	0	antisense	-1.6		Transportin or cytosolic karyopherin beta 2		-2.3	Galactose-1-phosphate uridyl transferase
298881..299131_X	X	298881..299131	298,972	251	3.11	0.00	APQ13	4	antisense	SMC3	25	antisense			Putative protein of unknown function		-2.3	Stability of MiniChromosomes
57664..57867_I	I	57664..57867	57,810	204	1.77	0.04	BOL3	278	antisense	YAL045C	0	antisense		-1.6	BolA-like protein		-2.3	Dubious open reading frame
755340..755569_XIV	XIV	755340..755569	755,415	230	1.89	0.03	YNR066C	304	antisense	DSE4	176	antisense			Putative membrane-localized protein of unknown function		-2.5	endo-1,3(4)-beta-glucanase
594645..594909_X	X	594645..594909	594,797	265	2.07	0.02	GRR1	317	antisense	JSN1	151	antisense		-1.8	Glucose Repression-Resistant		-2.6	RNA-binding protein Just Say No
604763..604884_X	X	604763..604884	604,825	122	2.06	0.02	FIP1	559	antisense	IME1	0	antisense		-1.9	Factor Interacting with Poly(A) polymerase		-2.9	Inducer of MEiosis; master regulator of early meiotic genes
48332..48517_I	I	48332..48517	48,405	186	2.55	0.01	FLC2	81	sense	OAF1	46	sense		-2.6	flavin adenine dinucleotide transporter FLC2		-3.0	Oleate-Activated transcription Factor
28953..29155_XII	XII	28953..29155	29,081	203	1.92	0.03	YLL056C	647	antisense	YCT1	953	sense			NADH-dependent aldehyde reductase		-3.8	Yeast Cysteine Transporter

Supplementary Table S11. Yeast TF motif enrichment in PS-specific, DM-specific and PS+DM shared promoter binding sites of potential target genes. Significantly enriched motifs were identified using the MEME Suite v 5.5.5 SEA module. The number of binding site sequences matching a threshold score for each motif is indicated, with supporting P-values and E-values (= P-value * 732 motifs in the YEASTRACT_20130918 database). Enrichment values are relative to frequencies for each motif in randomized primary sequence controls.

motif_ID	motif_consensus	PS-Specific	DM-Specific	PS+DM-Shared	Enrichment Ratio	P-Value	E-Value
Sko1p	TGACGTTT	270			1.3	1.32E-02	9.69E+00
Zap1p	ACCGTCACTGC	254			1.5	4.96E-04	3.63E-01
Dot6p	GCGATGAG	215			1.7	3.17E-03	2.32E+00
Abf1p	RTCAYTNTNTACGR	179			1.5	1.74E-03	1.28E+00
Stp3p	GCTAGCGCA	141			1.6	1.18E-03	8.66E-01
Gln3p	TATTGCTGATAAGGCCNHAA	138			1.7	5.32E-04	3.89E-01
Leu3p	CCGTTAACGG	134			1.4	6.33E-03	4.63E+00
Mcm1p	ATTTCCGAWWTGGGAAANA	82			1.9	2.93E-03	2.15E+00
Fkh1p, Fkh2p	GTMAACAA		393		1.3	1.30E-02	9.49E+00
Crz1p	GGGTGGCTG		74		1.6	1.05E-02	7.68E+00
Reb1p	MGGGTAAB		43		2.5	8.84E-04	6.47E-01
Abf1p	RTCaYtxxxxACGD		34		2.3	5.75E-03	4.21E+00
Matalpha2p	ATTGTT			30	3.1	1.74E-03	1.27E+00
	Total Binding Site Sequences:	545	475	74			

Supplementary Table S12 - Motif discovery in PS and DM binding sites in promoters of yeast DEG using the XSTREME module of MEME 5.5.5 suite (https://meme-suite.org/meme/tools/xstreme). Consensus sequences for enriched motifs are shown. Comparison with yeast TF motifs was facilitated using the Tomtom search tool.

Alt_ID	Logo	Consensus	Width (bp)	Sites	Total Sites	Percent Total Sites	*P*-Value	*E*-Value	Yeast Motif Comparison (Tomtom)	
									Motif ID	Motif Consensus
**PS-Specific Binding Sites**										
MEME-1	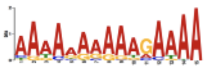	AAAAAAAAAAGAAAA	15	87	542	16.05%	9.55E-05	6.80E-26	Azf1p	AAAAGAAA
MEME-2	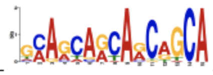	GMARCARCARCAGCA	15	29	458	6.33%	2.02E-03	4.10E-07	Rfx1p	CTATTGCTGCAAC
SEA-1	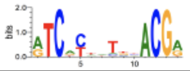	RTCDYWWTBHACGR	14	22	429	5.13%	4.40E-05	3.22E-02	Abf1p	RTCAYTNTNTACGR
SEA-2	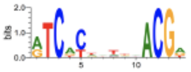	RTCDCDWNDNACGD	14	25	429	5.83%	3.30E-05	2.42E-02	Abf1p	RTCAYTNNNNACGD
**DM-Specific Binding Sites**										
MEME-1	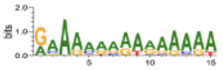	GAAAAAAAARAAAAA	15	170	475	35.79%	5.51E-07	8.60E-65	Azf1p	AAGAAAAA
STREME-1	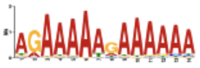	AGAAAAAGAAAAAA	14	61	475	12.84%	4.30E-02	1.70E-01	Azf1p	AAGAAAAA
**PS+DM Shared Binding Sites**										
MEME-1	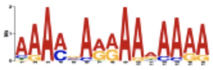	AAAMSARRAAAAAAA	15	26	74	35.14%	2.10E-04	3.00E-06	Azf1p	AAGAAAAA
