# Supplementary material for: Modified pea apyrase has altered nuclear functions and enhances the growth of yeast and Arabidopsis
Source: Front Plant Sci. 2025 May 26;16:1584871. doi: 10.3389/fpls.2025.1584871 (PMC12146327; doi:10.3389/fpls.2025.1584871)
Supplement: Supplementary file 2 [file DataSheet2.pdf]

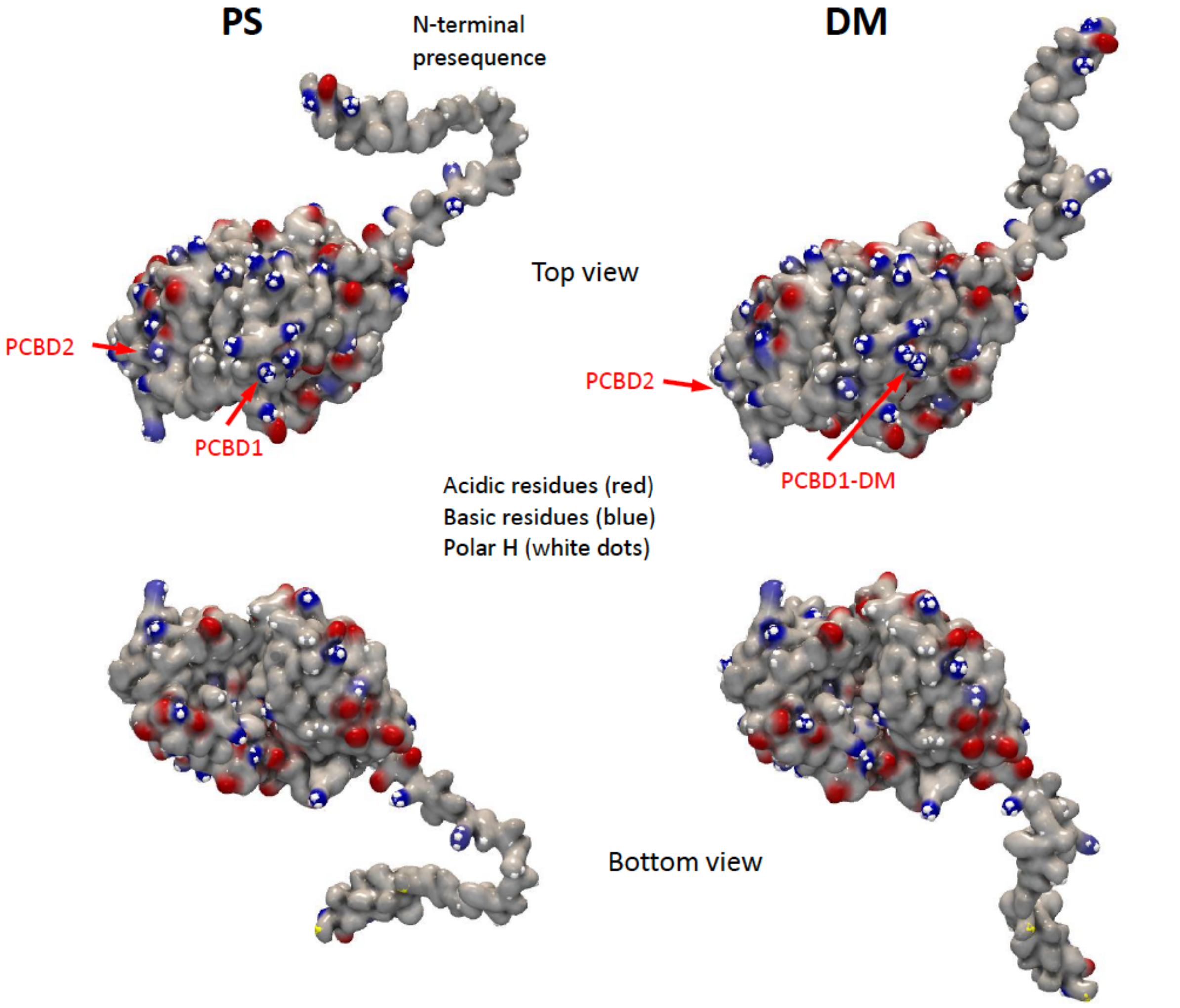

Supplementary Figure S2. Space-filling models of precursor PS and DM proteins showing the clustering of surface basic residues near the putative calmodulin-binding sites (PCBD1, PCBD2).

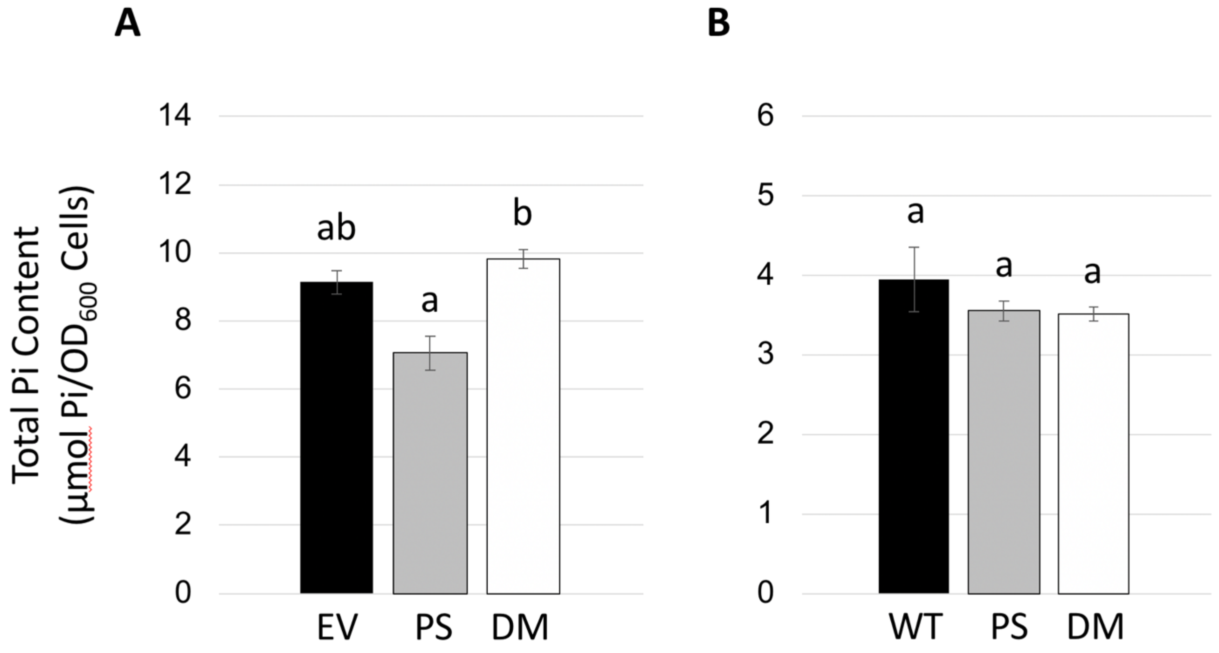

Supplementary Figure S2. Total Pi contents of yeast or Arabidopsis seedlings grown at low Pi. A) Yeast cells grown for 72 h in synthetic medium containing 0.1 mM Pi. Yeast cells were transformed with pYES2 empty vector (EV), pYES2+psNTP9 (PS) or pYES2+psNTP9-DM (DM). B) Day 12 Arabidopsis wild-type (WT), PS or DM seedlings were grown on 1/2x MS medium (0.6 mM Pi). Data are means ± S.E., n = 5 biological replicates. Letters indicate significant differences, as determined by one-way analysis of variance (ANOVA) with *post-hoc* Tukey honest significant difference (HSD) testing ($p < 0.05$).

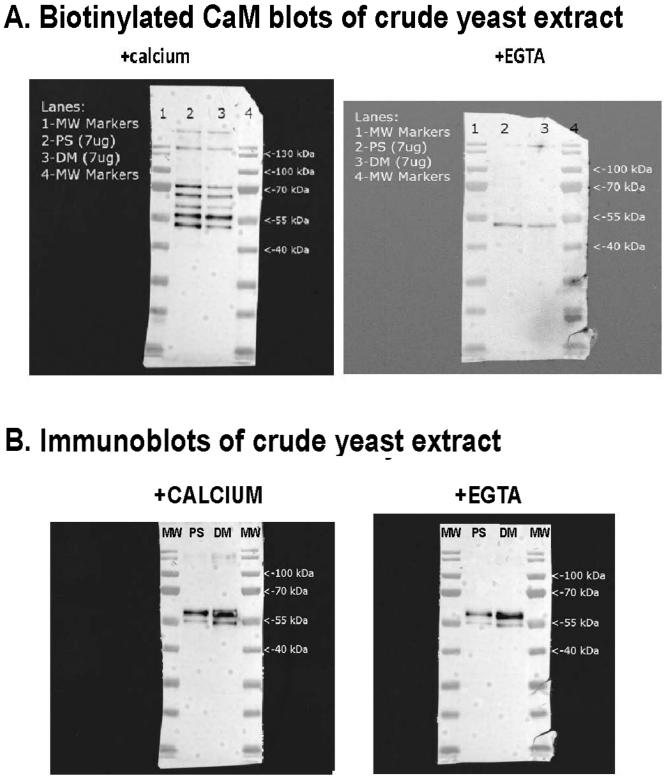

Supplementary Figure S3. (A) Biotinylated CaM assay of crude extract of yeast cells expressing PS or DM, in the presence (left panel) or absence (right panel) of Ca^{2+}. **(B)** Immunoblots of same crude extract used for **(A)**, using monoclonal antibody 8B6. PS and DM were both immunostained in the band just above the 55 kDa marker. Relative stain intensities of DM compared to that of PS, taken as 1.0: **(A)**, left panel, DM = 0.99; **(B)**, left panel, DM = 1.31; right panel, DM = 1.36.

A

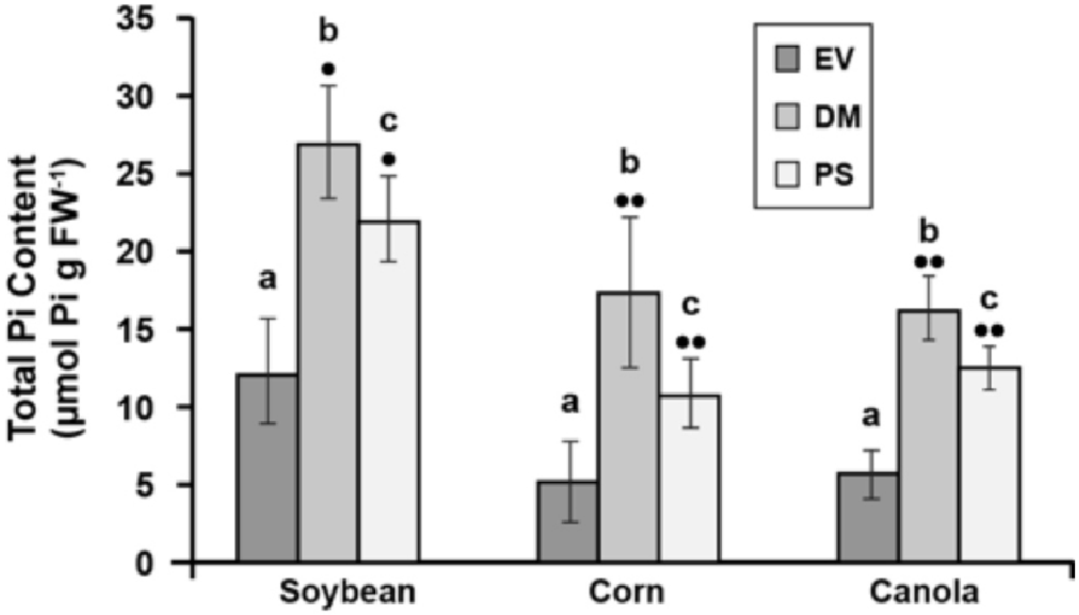

Supplementary Figure S4, panel A. *PS* and *DM* overexpression significantly enhances the phosphate (Pi) content of soybean, corn, and canola hairy root cultures transformed with *EV*, *PS* or *DM*. The Pi content of the individual hairy root culture lines was analyzed separately and then the average Pi content of the separately transformed lines was calculated ± SD (n=4-9 for EV lines; n = 8-10 for *PS* and *DM* lines). Different letters above the bars indicate significantly different values (*p < 0.005; **p < 0.0005).

B

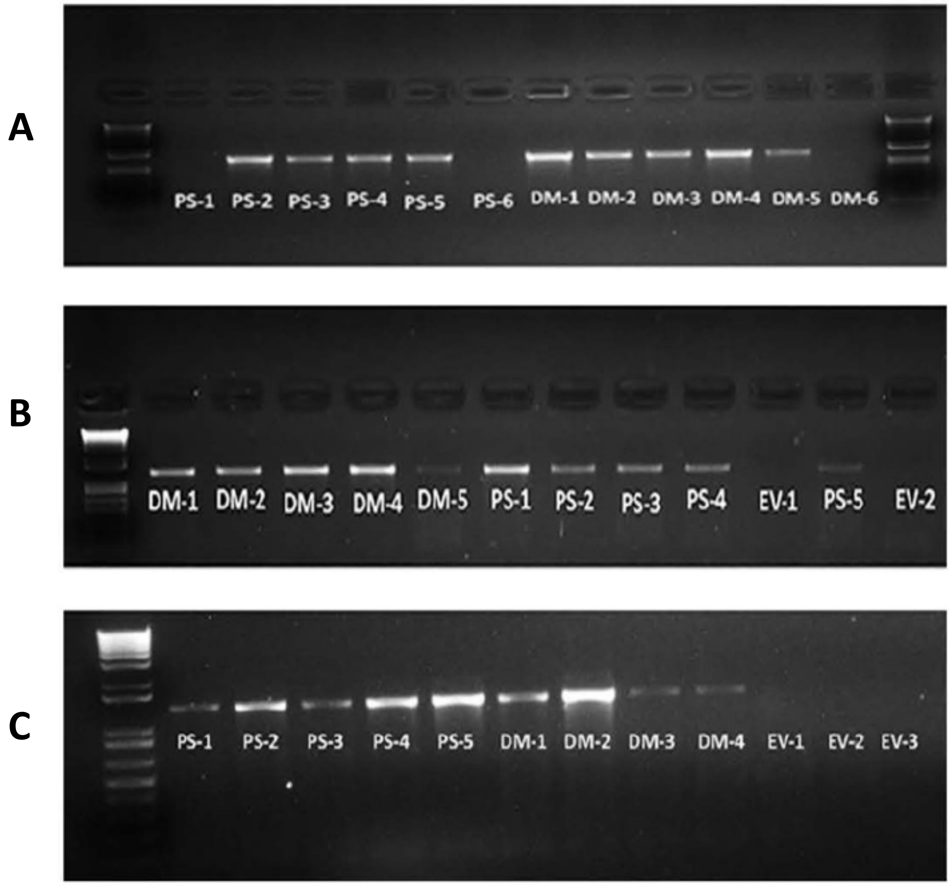

Supplementary Figure S4, panel B. RT-PCR verification of *PS* and *DM* transcripts in hairy roots of corn (A), canola (B) and soybean (C). EV – empty vector controls.

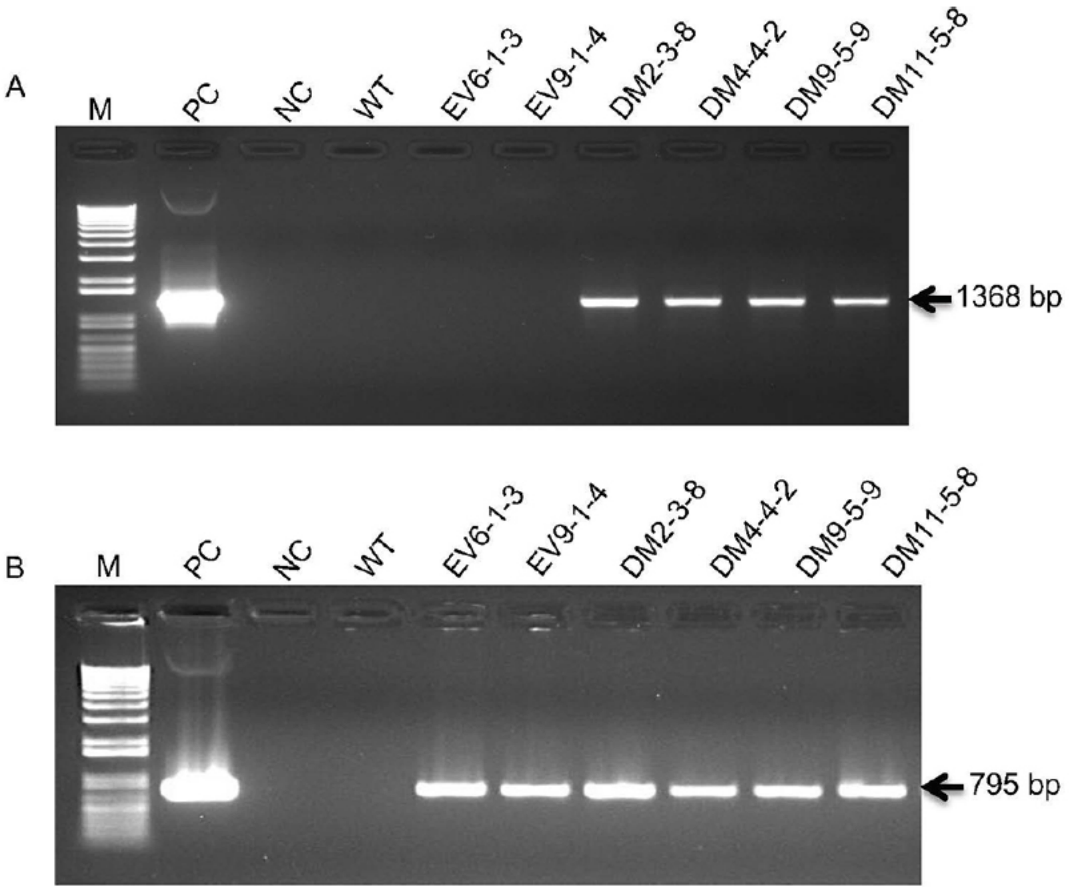

Supplementary Figure S5. RT-PCR confirmation of the full-length *DM* transcript (A) and *nptII* transcript (B) in transgenic Arabidopsis lines using gene-specific primers (**Supplemental Table S10**). EV – empty vector control, PC – positive control (purified pK7WG2:psNTP9-DM recombinant plasmid), NC – negative control (no template), WT – untransformed wild type.

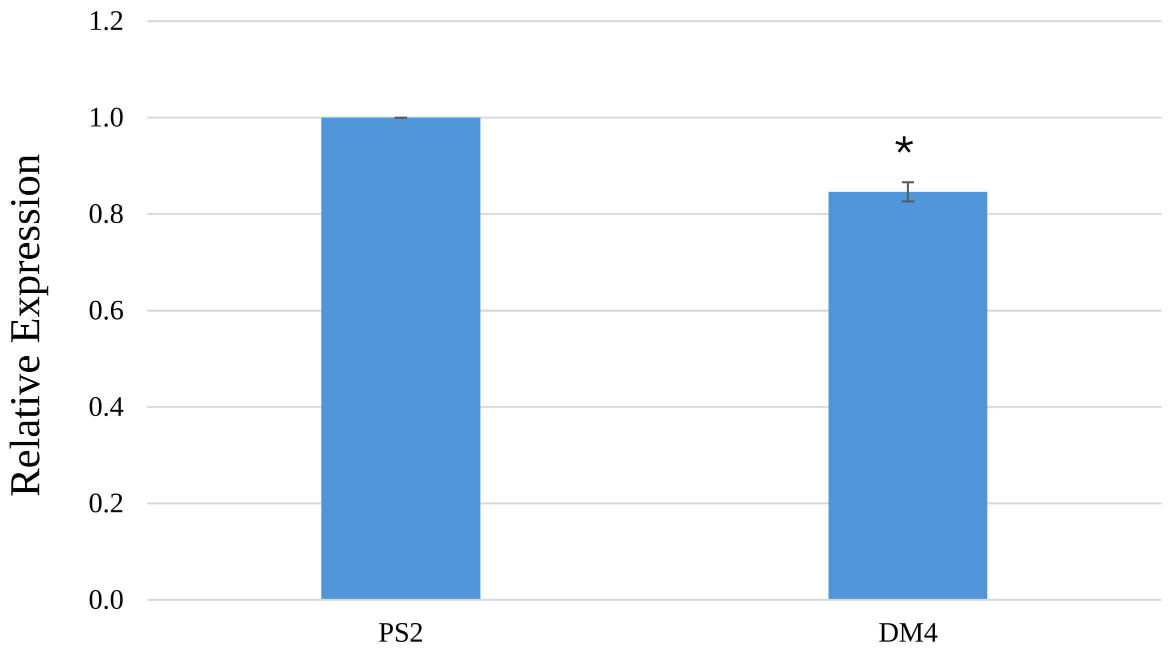

Supplementary Figure S6. Relative levels of *PS* and *DM* transcripts in Arabidopsis *PS*- and *DM*-expressing lines (PS2 and DM4), assayed by qRT-PCR. Total RNA was isolated from 7-d-old light-grown seedlings, and *PP2A* was used as the reference gene. The relative expression levels were normalized to the expression level in PS2, taken as 1.0. Col-0 WT was used as a negative control and no expression was detected. Data shown are the average of 3 biological replicates + s.d. * $p<0.05$, Student's *t*-test.

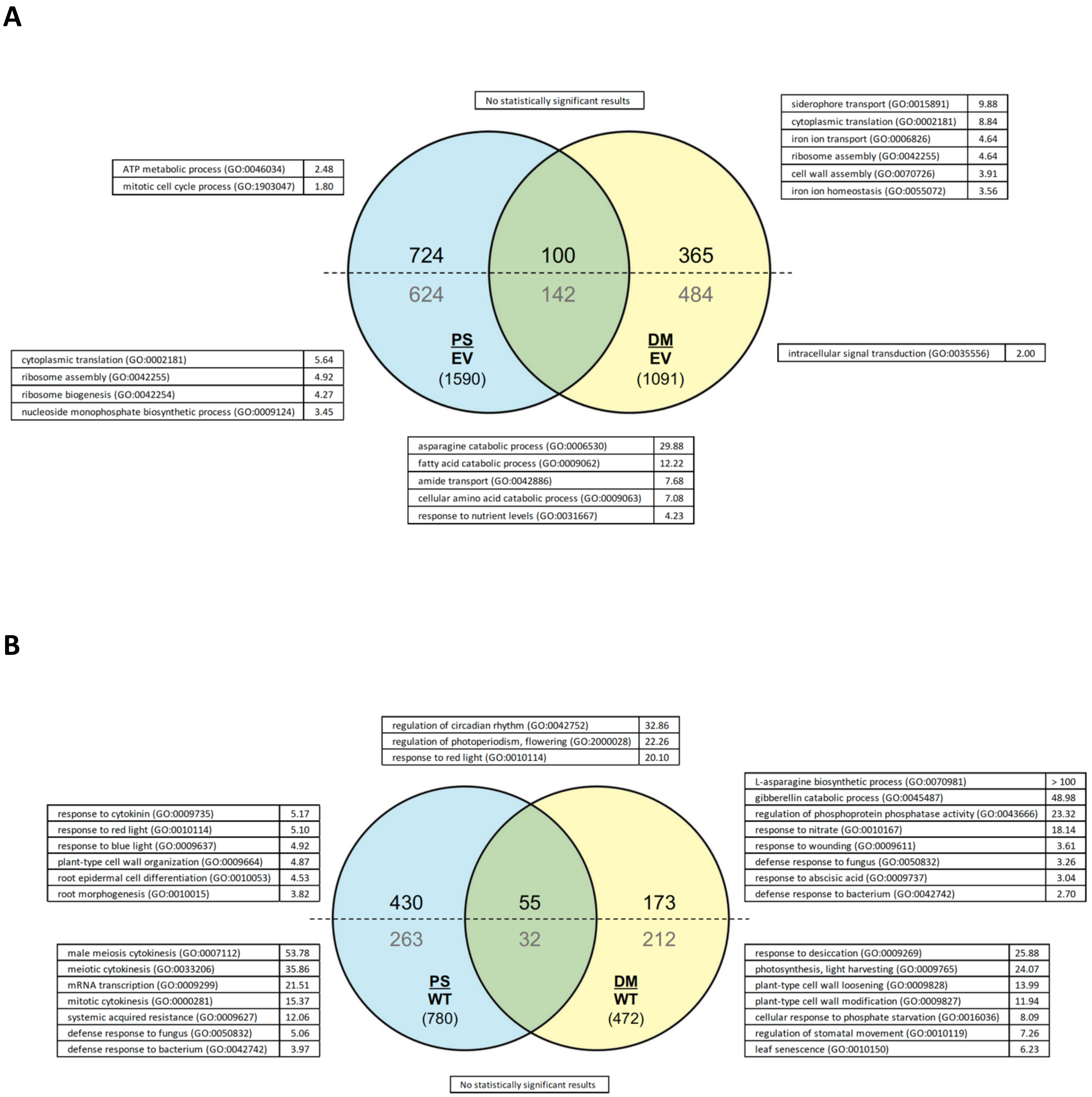

Supplementary Figure S7. Comparison of DEG in yeast (A) and Arabidopsis seedling (B) *PS* and *DM* lines reveals relatively little overlap between sets of induced and repressed genes. Numbers of DEG in each dataset are indicated in parentheses. Numbers of induced or repressed genes are indicated in black and gray, respectively. Overrepresented GO Bio Process categories and fold-enrichment values for genes in each category are shown.

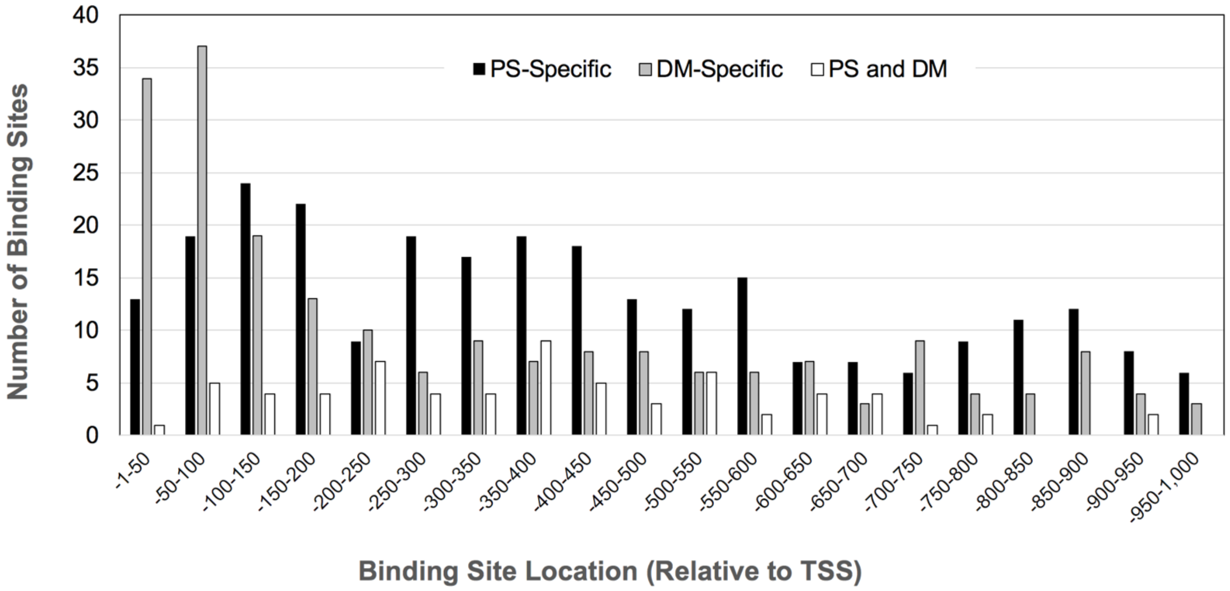

Supplementary Figure S8. Location of PS-specific, DM-specific and PS+DM shared binding sites in promoters of DE potentially-regulated target genes.
